# Communication strategies in the prevention of type 2 diabetes and gestational diabetes in vulnerable groups: a scoping review

**DOI:** 10.1186/s13643-021-01846-8

**Published:** 2021-11-24

**Authors:** Jessica Breuing, Christine Joisten, Annika Lena Neuhaus, Simone Heß, Lena Kusche, Fabiola Haas, Mark Spiller, Dawid Pieper

**Affiliations:** 1 Institute for Research in Operative Medicine (IFOM), Department for Evidence Based Health Service Research, Faculty of Health, Department of Medicine, Witten/Herdecke University, Ostmerheimer Strasse 200, Building 38, 51109 Cologne, Germany; 2 Institute of Movement and Neurosciences, German Sport University Cologne, Am Sportpark Müngersdorf 6, 50933 Cologne, Germany

**Keywords:** Type 2 diabetes mellitus, Prevention, Vulnerable groups, Communication strategies

## Abstract

**Background:**

The global prevalence of diabetes is nearly 9%, with an upward trend in type 2 diabetes mellitus (T2DM) and gestational diabetes (GDM). Although evidence shows that vulnerable groups are affected disproportionally, these groups are difficult to reach in terms of preventive measures. Currently, there is no gold standard regarding communication strategies and/or public awareness campaigns.

**Methods:**

We conducted a scoping review in September 2019. Two reviewers independently screened the results of the electronic literature search in several databases, including Medline, EMBASE, and PsycINFO. Extracted data were charted, categorized, and summarized.

**Results:**

All of the included articles (*n*=24) targeted T2DM; none targeted GDM. We identified the following five different vulnerable groups within the identified studies: migrants (*n*=9), ethnic groups such as African Americans (*n*=8), people with low socioeconomic status (*n*=3), older people (*n*=1), and people in need of care (*n*=1). Three categories of communication strategies were identified as follows: adapted diabetes prevention programs (*n*=21), community health workers (*n*=5), and technical approaches (*n*=9).

**Conclusion:**

We found different approaches for preventive interventions for T2DM. Some of these approaches were already adapted to known barriers. Communication strategies should be adapted to barriers and facilitating factors to increase participation and motivation.

**Supplementary Information:**

The online version contains supplementary material available at 10.1186/s13643-021-01846-8.

## Background

The global prevalence of diabetes mellitus is nearly 9% [1], with 90% of patients having type 2 diabetes mellitus (T2DM). Additionally, the prevalence of gestational diabetes mellitus (GDM) is increasing with approximately 16% of all live births being affected by hyperglycemia [2]. Because of its health consequences, the global health-related costs are expected to nearly double from the US $1.3 trillion in 2015 to $2.5 trillion by 2030, taking past trends into account [3]. This equals an increase in costs as a share of global gross domestic product from 1.8% in 2015 to a maximum of 2.2% in 2030 [3].

Many cases of T2DM/GDM could be prevented with lifestyle changes, including maintaining a healthy body weight, consuming a healthy diet, and staying physically active [4]. Therefore, there is an increasing need to implement effective preventive policies and to promote healthy lifestyles.

Ethnicity and/or lower socioeconomic status are important considerations in individuals affected by diabetes. For example, people in the lowest socioeconomic groups are 2.5 times as likely, and black and minority ethnic groups are up to six times as likely, to develop diabetes compared with the general population [5]. This could partly be attributed to lifestyle factors such as obesity, which more severely affect deprived communities and those living in vulnerable circumstances [6]. Yet, these vulnerable populations are difficult to reach in terms of preventive measures [6].

Numerous studies demonstrated that T2DM can be prevented or delayed by intensive lifestyle changes in individuals with prediabetes [7]. However, little is known regarding effective communication or awareness strategies for primary prevention of T2DM/GDM, in particular, regarding accessibility to those who are hardest to reach and most at risk. Identifying barriers and facilitators is necessary to increase the number of participants in preventive interventions that address vulnerable groups. Just as importantly, we must determine communication strategies to access participants, especially those in vulnerable groups. Existing programs like the diabetes prevention program (DPP) by the National Institute of Diabetes and Digestive and Kidney Diseases focus on lifestyle changes (e.g., weight loss, physical activity) as a key element in preventing diabetes mellitus exclusively in the general population [8, 9]. Therefore, we aimed to identify translations or modifications of existing programs or new communication strategies for vulnerable groups. Our target audiences are primary care providers (e.g., general practitioners, nutritionists, and midwives) as well as diabetologists and public health experts actively involved in diabetes prevention. Previously published articles demonstrate a need for a systematic approach to scope the current literature [10, 11]. This study is a part of a large project commissioned by the Federal Center for Health Education to develop a national education and communication strategy in preventing diabetes mellitus.

The aim of this study was to systematically review the literature in order to identify and describe communication strategies for preventing T2DM/GDM in vulnerable groups.

## Methods

This project was commissioned by the Federal Center for Health Education in Germany as part of the national education and communication strategy on diabetes mellitus. The project consists of two scoping reviews, one aimed to identify barriers and facilitating factors [12] and the other aimed to identify communication strategies for diabetes prevention in vulnerable groups. This report focuses on communication strategies. We used the same literature search for both scoping reviews. The methods were in accordance with those stated in a previously published protocol [13]. Deviations are outlined below.

The scoping review was conducted following Arksey and O’Malley’s framework [14] and the Joanna Briggs Institute Reviewers’ Manual 2015 [15]. Furthermore, the Preferred Reporting Items for Systematic Reviews and Meta-Analyses Extension for Scoping Reviews (PRISMA-ScR) Checklist [16] was used. Since the International Prospective Register of Systematic Reviews (PROSPERO) does not register scoping reviews, this scoping review was not registered.

### Eligibility criteria

#### Inclusion criteria

- Vulnerable patients with, or at risk of, T2DM/GDM
- Studies presenting barriers and facilitating factors for implementing a preventive or health-promoting intervention (primary or secondary prevention)
- World Health Organization (WHO) mortality stratum A countries
- Publication date no earlier than 2008

#### Exclusion criteria

- Indigenous people, children, or people with mental disorders
- No full text available
- General prevention with no context of T2DM/GDM
- Studies including patients on oral antidiabetic or insulin medications

Eligibility criteria are additionally shown in the PPC (Population, Concept, Context) mnemonic, which is presented in the protocol [13]. We included studies presenting communication strategies for the prevention of T2DM/GDM in vulnerable groups. Publications were restricted to studies published from January 2008 onward. External factors such as accessibility of care and information possibly affect communication strategies. We assume that, over the past 10 years, there has been a change in accessibility due to the volume of digital and virtual goods, services, and processes in healthcare. Therefore, we chose a 10 year search period, because communication strategies might have changed, so that there would be a lack of comparability if we chose a longer period. No language restrictions were made. If necessary, full texts were translated. Furthermore, we included only those studies with a low mortality stratum (A) according to the WHO [17], thus ensuring that our findings would be applicable to western industrialized countries. We defined vulnerable groups as a group of people in a disadvantaged position due to factors usually considered outside their control (e.g., race). To operationalize this, we used a list presented by Lewis et al. [18], except we excluded indigenous people, children, and people with mental disorders. These exclusion criteria were added after the protocol was published. This project was commissioned by Federal Centre for Health Education in Germany as part of the “National education and communication strategy on diabetes mellitus.” The results will be used to build a national education strategy in Germany. Since Germany does not have a representative proportion of the indigenous population and due to the narrow time frame of the project, we excluded this subgroup. Because it was necessary to define and separate primary and secondary from tertiary prevention or therapy, we excluded studies with patients treated with any antidiabetic medication (e.g., metformin or insulin).

### Information sources

The following electronic databases were searched: MEDLINE via PubMed, EMBASE via Elsevier, PsycINFO via Ebsco PSYNDEX via EbscoCINAHL via Ebsco, and Social Science Citation Index via Clarivate Analytics. Gray literature was searched in greylit.org and through the homepages of the WHO and international, healthcare, or public health departments (e.g., Department of Health and Social Care, UK; Agency for Healthcare Research and Quality; and the U.S. Preventive Services Task Force). We searched manually for additional studies by cross-checking the reference lists of all included studies.

### Search

The search strategy was developed by the research team in collaboration with an experienced librarian and checked by a referee according to the Peer Review of Electronic Search Strategies (PRESS) guideline [19]. For all databases, the initial search was conducted in May 2018, but the gray literature was searched in July 2018. An update of the initial database searches was conducted in September 2019. An update of the initial gray literature search was not performed because the initial search was very time-consuming and not worthwhile. The search strategy is presented as Supplement 1.

### Data management

The search results were uploaded and managed using Microsoft Excel. A PRISMA flow diagram was used to summarize and visualize the study selection.

### Study selection

Two reviewers independently screened titles and abstracts of the search results against the inclusion criteria. Subsequently, two reviewers independently screened full-text reports for potential eligibility. Any disagreement was resolved through discussion and consensus. The reasons for the exclusion of full texts were documented. A list of included studies in addition to study characterization is shown in Table 1, and a list of excluded studies is provided as Supplements 2.

**Table 1:** Study characteristics of all included studies

Study	Study design	Participants [*n*]	Gender [m/f]*n*(%)	Age[years] mean(%)//*n*(%)//range	Vulnerable group category
Bender MS, Cooper BA, Flowers E, Ma R, Arai S. Filipinos Fit and Trim - a feasible and efficacious DPP-based intervention trial. Contemporary Clinical Trials Communications. 2018;12:76-84	Intervention	67	32(48)/35(52)	41.7	Migrants
Blanks SH, Treadwell H, Bazzell A, Graves W, Osaji O, Dean J, McLawhorn JT, Stroud JL: Community engaged lifestyle modification research: engaging diabetic and prediabetic african american women in community-based interventions. J Obes 2016, 2016:3609289	Intervention	79	0(0)/79(100)	29	Ethnic group
Bolin JN, Ory MG, Wilson AD, Salge L. Diabetes education kiosks in a latino community. The Diabetes educator. 2013;39(2):204-12.	Mixed Methods	Use: 5372 Exit-Survey: 179	55(30.8)/115(63.7)	<18 18 (9.9) 19–35 28 (15.4) 36–49 71 (39.0) 50–64 44 (24.2) 65+ 10 (5.5) n.r. 11 (6.5)	Ethnic group
Borelli MR, Riden HE, Bang H, Schenker MB. Protocol for a cluster randomized controlled trial to study the effectiveness of an obesity and diabetes intervention (PASOS) in an immigrant farmworker population. BMC Public Health. 2018;18(1):849.	Protocol	n.a.	n.a.	n.a.	Migrants
Castro-Rivas E, Boutin-Foster C, Milan M, Kanna B. “Es como uno bomba de tiempo [It's like a time bomb]”: a qualitative analysis of perceptions of diabetes among first-degree relatives of latino patients with diabetes. Diabetes Spectrum. 2014;27(1):50-7.	Focus groups	23	15(65)/8(35)	46.4	Migrants
Chang MW, Nitzke S, Brown R, Resnicow K. A community based prevention of weight gain intervention (Mothers In Motion) among young low-income overweight and obese mothers: design and rationale. BMC public health. 2014;14:280.	RCT	IG/CG 410/202	0(0)/612(100)	28.1	LSES
Fischer HHF, I.P.; Pereira, R.I.; Furniss, A.L.; Rozwadowski, J.M.; Moore, S.L.; Durfee, M.J.; Raghunath, S.G.;Tsai, A.G;, and Edward P. Havranek1: Text message support for weight loss in patients with prediabetes: a randomized clinical trial. diabetes care 2016, 39:1364–1370.	RCT	IG/CG 51/34	IG: 13(25.5)/38(74.5) CG: 6(17.6)/28(82.4)	IG: 47.9 CG: 41.6	Migrants
Fontil V, McDermott K, Tieu L, Rios C, Gibson E, Sweet CC, et al. Adaptation and feasibility study of a digital health program to prevent diabetes among low-income patients: results from a partnership between a Digital Health Company and an Academic Research Team. Journal of diabetes research. 2016;2016:8472391.	Feasibility Study	18	8(47.0)/10(53.0)	53.0	LSES
Ford AF, Reddick K, Browne MC, Robins A, Thomas SB, Crouse Quinn S. Beyond the cathedral: building trust to engage the African American community in health promotion and disease prevention. Health promotion practice. 2009;10(4):485-9.	Review	n.a.	n.a.	n.a.	Ethnic group
Fukuoka Y, Vittinghoff E, Hooper J. A weight loss intervention using a commercial mobile application in Latino Americans-Adelgaza Trial. Translational Behavioral Medicine. 2018;8(5):714-23.	Intervention	54	17(31.0)/37(69.0)	45.3	Migrants
Gary-Webb TL, Walker EA, Realmuto L, Kamler A, Lukin J, Tyson W, et al. Translation of the National Diabetes Prevention Program to Engage Men in Disadvantaged Neighborhoods in New York City: a description of power up for health. American journal of men's health. 2018:1557988318758788.	Focus groups	29	29(100)/0(0)	n.r.	LSES
Gutierrez J, Devia C, Weiss L, Chantarat T, Ruddock C, Linnell J, et al. Health, community, and spirituality: evaluation of a multicultural faith-based diabetes prevention program. The Diabetes educator. 2014;40(2):214-22.	Intervention	183	21(11.7)/159(88.3)	18–44: 25(15.6) 45–64: 83(51.9) 65–74: 40(25.9) 75+: 12(7.5)	Ethnic group
Hall D, Lattie E, McCalla J, Saab P. Translation of the diabetes prevention program to ethnic communities in the United States. Journal of Immigrant & Minority Health. 2016;18(2):479-89.	Review	n.a.	n.a.	n.a.	Ethnic group
Handley MA, Harleman E, Gonzalez-Mendez E, Stotland NE, Althavale P, Fisher L, et al. Applying the COM-B model to creation of an IT-enabled health coaching and resource linkage program for low-income Latina moms with recent gestational diabetes: the STAR MAMA program. Implementation science : IS. 2016;11(1):73.	Focus groups	22	0(0)/22(100)	31.5	Migrants
Harvey I, Schulz A, Israel B, Sand S, Myrie D, Lockett M, et al. The Healthy Connections project: a community-based participatory research project involving women at risk for diabetes and hypertension. Progress in community health partnerships: research, education, and action. 2009;3(4):287-300.	Intervention	1428	164 (11.5)/1264 (88.5)	18–29: 93(10.0) 30–44: 223(24.0) 45-59: 287(31.0) 60+: 325(35.0) N.R. 500	Ethnic group
Kato S, Ando M, Kondo T, Yoshida Y, Honda H, Maruyama S. Lifestyle intervention using Internet of Things (IoT) for the elderly: a study protocol for a randomized control trial (the BEST-LIFE study). Nagoya J Med Sci. 2018;80(2):175-82.	Protocol	n.a.	n.a.	n.a.	Older people
Kim SE, Castro Sweet CM, Gibson E, Madero EN, Rubino B, Morrison J, et al. Evaluation of a digital diabetes prevention program adapted for the Medicaid population: study design and methods for a non-randomized, controlled trial. Contemp Clin Trials Commun. 2018;10:161-8.	Intervention	230	44(19.1)/186(80.9)	48	LSES
Newton RL, Jr., Johnson WD, Larrivee S, Hendrick C, Harris M, Johannsen NM, et al. A randomized community-based exercise training trial in African American men: ARTIIS. Med Sci Sports Exerc. 2019.	RCT	IG/CG 54/49	103(100)/0(0)	51.8	Ethnic group
Nicolaou M, Vlaar E, van Valkengoed I, Middelkoop B, Stronks K, Nierkens V. Development of a diabetes prevention program for Surinamese South Asians in the Netherlands. Health promotion international. 2013;29(4):680-91.	Review	n.a.	n.a.	n.a.	Migrants
Ruggiero LO, S; Choi, J.K: Community-based translation of the diabetes prevention program’s lifestyle intervention in an underserved Latino population. The Diabetes EDUCATOR 2011, 37(4):564-672.	Intervention	69	0(0)/69(100)	37.9	Migrants
Siddiqui F, Koivula RW, Kurbasic A, Lindblad U, Nilsson PM, Bennet L. Physical activity in a randomized culturally adapted lifestyle intervention. Am J Prev Med. 2018;55(2):187-96.	RCT	IG/CG 50/46	IG//CG 14 (43.7)/24(56.3)//18 (48.6)/28(51.4)	IG/CG 50.2/47.1	Migrants
Vincent D, McEwen MM, Hepworth JT, Stump CS. The effects of a community-based, culturally tailored diabetes prevention intervention for high-risk adults of Mexican descent. The Diabetes educator. 2014;40(2):202-13.	RCT	IG/CG 38/20	IG 9(23.7)/29(76.3) CG 4(20.0)/16(80.0)	IG/CG 50.0/52.8	Ethnic group
Walker EA, Weiss L, Gary-Webb TL, Realmuto L, Kamler A, Ravenell J, et al. Power Up for Health: Pilot study outcomes of a Diabetes Prevention Program for men from disadvantaged neighborhoods. American Journal of Men's Health. 2018;12(4):989-97.	Intervention	29	29(100)/0(0)	49.9	LSES
Whittemore R, Rosenberg A, Gilmore L, Withey M, Breault A. Implementation of a DIABETES PREVENTION PROGRAM IN PUBLIC HOUSING COMMUNITIES. Public Health Nursing. 2014;31(4):317-26.	Mixed methods	67	13(19.4)/54(80.6)	Diabetes prevention: 41.9 Standard care: 39.0	People in need of care

*LSES* low socioeconomic status, *n.a*. not applicable, *n.r*. not reported

### Data extraction

A standardized extraction form was developed for this review. A pilot test of the data extraction form was conducted on a sample of five articles by the reviewers involved in the scoping review to assess its completeness and applicability. Based on the pilot testing, modifications to the standardized data extraction form were needed and undertaken to ensure the data necessary to address the research questions were obtained. Data were extracted by one reviewer and checked by another. Disagreements were resolved through discussion and consensus. The data extraction form consisted of the following items:

- Study (name, year)
- Study design
- Participants (*n*)
- Gender [m/f] *n*(%)
- Age[years] mean(%)//*n*(%)//range
- Vulnerable group description from primary study *n*(%)
- Vulnerable group category (migrants, ethnic group, older people, disabled people, people in need of care, unemployed people, homeless people, drug abusers, low socio-economic status)
- Diabetes risk (prediabetic, diabetic)
- Inclusion criteria
- Exclusion criteria
- Setting (country, city, healthcare facility)
- Recruitment, prevention (primary, secondary, tertiary)
- Communication strategies/access
- Results: communication strategies (authors conclusion)

## Conclusion

### Data items

The preliminary data extraction categories were derived from our overarching research question. The following data were collected:

- Study characteristics (e.g., country, setting, publication date, number of participants, target disease, and study design/method)
- Patient characteristics (e.g., age, gender, and affiliation to the vulnerable group)
- Inclusion/exclusion criteria
- Communication strategies

### Risk of bias

As this was a scoping review, there was no risk of bias assessment. This is consistent with guidance on the conduct of scoping reviews [14].

### Data synthesis

We used Arksey and O’Malley’s methods [14] of reporting and provide a descriptive analysis of the extent, nature, and distribution of the studies included in the review as well as a narrative, thematic summary of the data collected. This was achieved by summarizing the literature according to the types of vulnerable groups, communication strategies, comparators, implementation factors, and outcomes identified. We aimed to map the research landscape in this area. This was facilitated by some form of visual representation of the data to map the extent, range, and nature of research in this area. Data were charted, categorized, and summarized. We reported quantitative (e.g., frequency) and qualitative results. Furthermore, we sought to explore similarities and differences, both within and between studies, to identify patterns and themes and to postulate explanations for findings. By doing so, we also considered the robustness of the included studies themselves by reporting on the overall strength of and confidence in the findings. If possible, we stratified our results by vulnerable groups.

### Data analysis

Assignment of subjects into the two vulnerable group migrants and ethnic group was made in the context of their labeling within the studies. For example, Mexican Americans were assigned to the ethnic group because we defined them as Americans with a Mexican background. If a group was labeled as Latinos/Latinas, we assigned them to the vulnerable group migrants. Categorization of the communication strategies was made in two steps. First, we tried to identify which communication strategies were used, and second, we tried to identify the main communication strategy focused on, if more than one strategy was used.

## Results

**Fig. 1:**
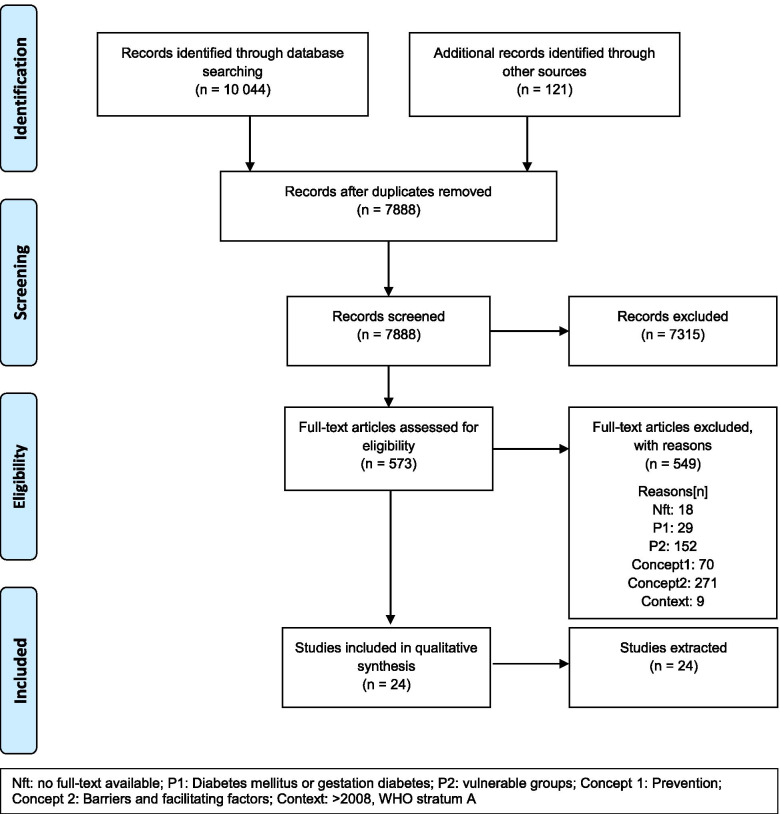
Flow chart

In our initial search, we identified 8584 articles, 1460 through the update search and 121 through gray literature search apart from the manual search for additional studies by crosschecking which sums up to 10,044 articles. After removing all duplicates, we screened 7888 articles. In total, 572 articles were assessed in full text for eligibility (see Fig. 1). Of these, 549 articles were excluded mostly because of the absence of communication strategies (*n*=271) or missing vulnerable groups (*n*=152). A list of excluded full-text studies is available as Supplement 2.

### Characteristics of included studies

The study design of the identified studies (*n*=24) ranged from interventional trials (randomized controlled trials (RCTs) (*n*=5), protocols for RCTs (*n*=2), and other study designs (*n*=8)) to qualitative studies (focus group (*n*=3) or mixed methods (*n*=2)). Additionally, we identified three narrative reviews and one feasibility study. All identified studies focused on T2DM; none focused on GDM. Most studies were conducted in the USA (*n*=15), one in the Netherlands, and one in Japan. We identified five different vulnerable groups within the identified studies: migrants (*n*=9), ethnic groups such as African Americans (*n*=8), people with a low socioeconomic status (*n*=3), older people (*n*=1), and people in need of care (*n*=1). The identified communication strategies were stratified into the following three categories: adapted diabetes prevention programs (ADPPs), community health workers (CHWs), and technical approaches (TAs). Table 2 shows the allocations to each category in addition to descriptions of the communication strategies in Table 3. All study characteristics are listed in Table 1. However, some of the included studies used more than one strategy, for example, some kind of DPP and a TA [20], in which case we pointed out the study’s main focus.

**Table 2:** Communication strategies of the included studies

Study	Setting	Methods/vulnerable group	Communication strategy	Category^a^
Bender et al., 2018 [20]	USA, California, San Francisco	Intervention/migrants	Fit&Trim DPP-based intervention with mobile technology	ADPP + **TA**
Blanks et al., 2016 [35]	USA, South Carolina, Columbia	Intervention/ethnic group	“I am Women” DPP (based on the National Diabetes Education Program) via CHW	ADPP + **CHW**
Bolin et al., 2013 [39]	USA, South-Texas	Mixed methods/ethnic group	Bilingual, computerized touchscreen diabetes education kiosk (Diosk) designed to provide “on-demand” education	**TA**
Borelli et al., 2018 [21]	USA, California, Oxnard	Protocol/migrants	Five-step lifestyle intervention in workplace environment with additional, voluntary activities	**ADPP**
Castro-Rivas et al., 2014 [22]	USA, New, York, South Bronx	Focus groups /migrants	Focus group was asked about possible interventions and strategies on how to prevent diabetes	**ADPP**
Chang et al., 2014. [40]	USA, Michigan	RCT/LSES	Intervention with culturally tailored video lessons	**TA**
Fischer et al., 2016 [36]	USA, Texas, Denver	RCT/migrants	Bilingual text messages relating to nutrition, physical activity, and motivation	ADPP + CHW + **TA**
Fontil et al., 2016 [41]	USA, California, San Francisco	Feasibility study/LSES	Digital health diabetes prevention and management program	ADPP + **TA**
Ford et al., 2009 [23]	USA, Pennsylvania, Pittsburgh	Review/ethnic group	Review on health promotion and disease prevention in African American community	**ADPP**
Fukuoka et al., 2018 [42]	USA, California, San Francisco	Intervention/migrants	Weight loss intervention using a commercial mobile application	ADPP + **TA**
Gary-Webb et al., 2018 [24]	USA, New York	Focus groups/LSES	Adaptation of the NDPP for men with a low socioeconomic status.	**ADPP**
Gutierrez et al., 2014 [25]	USA, New York, Harlem and Bronx	Intervention/ethnic group	Fine, Fit, and Fabulous: faith-based DPP	**ADPP**
Hall et al., 2016 [26]	USA, n.a.	Review/ethnic group	Culturally tailored DPP	**ADPP**
Handley et al., 2016 [43]	USA, California, San Francisco and Sonoma County	Focus groups/migrants	Health literacy-tailored health IT tool delivering DPP content	ADPP + **TA**
Harvey et al., 2009 [37]	USA, Michigan, Detroit	Intervention/ethnic group	CHW preventive information through house parties	**CHW**
Kato et al., 2018 [44]	Japan, Nagoya	Protocol/older people	Lifestyle intervention using Internet of Things	**TA**
Kim et al., 2018 [45]	USA, California, Los Angeles	Intervention/LSES	Language-and-literacy adapted digital DPP	ADPP + **TA**
Newton et al., 2019 [27]	USA, California, Los Angeles	RCT/ethnic group	Aerobic plus resistance training	**ADPP**
Nicolaou et al., 2013 [28]	Netherlands, The Hague	Review/migrants	The “DH!AAN” intervention consisted of culturally targeted lifestyle counseling using motivational interviewing targeting physical activity and diet, conducted by dieticians	**ADPP**
Ruggiero et al., 2011 [29]	USA, Illinois	Intervention/migrants	Community-based translation of the DDP	**ADPP +** CWH
Siddiqui et al., 2018 [30]	Sweden, Malmö	RCT/migrants	Adapted lifestyle intervention to increased physical activity and improved food habits	**ADPP**
Vincent et al., 2014 [31]	USA, Arizona, Tucson metropolitan area	RCT/ethnic group	Culturally tailored, community-based DPP for Spanish speaking adults of Mexican descent	**ADPP**
Walker et al., 2018 [32]	USA, New York	Intervention/LSES	ADPP (based on NDPP) for men from disadvantaged, urban neighborhoods (Power Up for Health)	**ADPP**
Whittemore et al., 2014 [38]	USA, Connecticut	Mixed methods/people in need of care	ADPP in public housing communities.	ADPP + **CHW**

*ADPP *adapted Diabetes Prevention Program, *CHW* community health worker, *DPP* Diabetes Prevention Program, *LSES* low socioeconomic status, *n.a.* not applicable, *NDPP* National Diabetes Prevention Program, *TA* technical approach, *RCT* randomized controlled trial

^a^The main focus of the study is shown in bold

**Table 3:** Study description of all included studies

Study	Study description
Bender MS, Cooper BA, Flowers E, Ma R, Arai S. Filipinos Fit and Trim - A feasible and efficacious DPP-based intervention trial. Contemporary Clinical Trials Communications. 2018;12:76-84	*Attended 5 in-person intervention office visits *Tracked real-time steps by wearing a fitbit zip on their torso at least 10 h/day *Logged daily food/drink intake and weekly home weights on a mobile app/diary *Received weekly postings of discussion topics (related to weight loss, pa and healthy eating) on the study’s private facebook group site to reinforce healthy behaviors learned during in-person intervention sessions *Prior to receiving the intervention, participants were trained on how to (1) use the fitbit zip; download the mobile app/diary and (2) access and join the private facebook group. Participants’ fitbit data was monitored by research staff and uploaded in real-time to the study’s online account and secure data servers. Participants experiencing technological problems received remote assistance via phone.
Blanks SH, Treadwell H, Bazzell A, Graves W, Osaji O, Dean J, McLawhorn JT, Stroud JL: Community engaged lifestyle modification research: engaging diabetic and prediabetic African American women in community-based interventions. J Obes 2016, 2016:3609289	The curriculum includes the following sessions: *Introduction to the healthy lifestyle program *Nutrition and chronic disease *Nutritional literacy/building nutritional competence *Combating stress and emotional eating *Strategies for health eating and exercise *Partnering with your healthcare provider *Celebrating your healthier family other resources and activities: *Physical activity planning section *Healthy eating recipe *Separate youth focused curriculum which supplements the adult sessions *Community Resource Guide *Field trip/activity planner
Bolin JN, Ory MG, Wilson AD, Salge L. Diabetes education kiosks in a latino community. The Diabetes educator. 2013;39(2):204-12.	Bilingual diosk modules: *What is diabetes? *Are you at risk for diabetes? *Preventing diabetes *High and low blood sugar *Complications related to diabetes *Healthy recipes *Medications and daily management *Meal planning *Kids’ corner *Signs and symptoms of diabetes *Exercise *Sick days, disasters, and special events
Borelli MR, Riden HE, Bang H, Schenker MB. Protocol for a cluster randomized controlled trial to study the effectiveness of an obesity and diabetes intervention (PASOS) in an immigrant farmworker population. BMC Public Health. 2018;18(1):849.	The five steps intervention *Move *Drink water *Eat fruits and vegetables *Measure (food portions and weight) *Share (information learned and healthy habits) Examples of additionally voluntary activities: *Cooking demonstrations *Education on specific health topics *Cultural celebrations with modified recipes *Walking challenges, and Zumba dance sessions *Use of the “Salud para su Corazon” curriculum ( bilingual program for promotors developed specifically for Latino communities)
Castro-Rivas E, Boutin-Foster C, Milan M, Kanna B. “Es como uno bomba de tiempo [It’s like a time bomb]”: a qualitative analysis of perceptions of diabetes among first-degree relatives of Latino patients with diabetes. Diabetes Spectrum. 2014;27(1):50-7.	Proposal for intervention *As involving community stakeholders and community engagement *Identifying culturally relevant media To deliver health messages is crucial *Nontraditional venues such as schools, social clubs, and churches *Modifying and enhancing the physical environment and investigating the excess density of unhealthy options must be addressed
Chang MW, Nitzke S, Brown R, Resnicow K. A community based prevention of weight gain intervention (Mothers In Motion) among young low-income overweight and obese mothers: design and rationale. BMC public health. 2014;14:280.	Ig: *Program for 16 weeks *Culturally tailored dvds (~20 min, weekly from weeks 1–4, every second week from week 5–16) with 11 chapters for stress management, healthy nutrition, physical activity, and group teleconferences) *Additionally weekly peer support group teleconferences for group discussions about the video’s content Cg: *Printed materials from standard reliable sources for stress management, healthy eating and physical activity and a dvd about food and home safety (~10 min)
Fischer HHF, I.P.; Pereira, R.I.; Furniss, A.L.; Rozwadowski, J.M.; Moore, S.L.; Durfee, M.J.; Raghunath, S.G.;Tsai, A.G;, and Edward P. Havranek1: Text message support for weight loss in patients with prediabetes: a randomized clinical trial. Diabetes Care 2016, 39:1364–1370.	Intervention *6 text messages per week (English or Spanish) relating to nutrition, physical activity, and motivation * once-weekly text message asking participants to report their most recent weight. *Motivational interviewing with a community health worker *Possibility to join the control groups DPP
Fontil V, McDermott K, Tieu L, Rios C, Gibson E, Sweet CC, et al. Adaptation and feasibility study of a digital health program to prevent diabetes among low-income patients: results from a partnership between a Digital Health Company and an Academic Research Team. Journal of diabetes research. 2016;2016:8472391.	*Previous examination of barriers via focus groups *It support for the first use of the program, especially for people with low computer capabilities *Informational event adapted to people with a low reading level *Bilingual program (english/spanish) *Telephone interviews during the whole program to find out further barriers
Ford AF, Reddick K, Browne MC, Robins A, Thomas SB, Crouse Quinn S. Beyond the cathedral: building trust to engage the African American community in health promotion and disease prevention. Health promotion practice. 2009;10(4):485-9.	*Possible interventions: Health-coaches, examination of genetic family history, smoking cessation, fitness-courses, yoga, dancing, nutrition counseling, prevention of depression, aerobics, management of chronic diseases
Fukuoka Y, Vittinghoff E, Hooper J. A weight loss intervention using a commercial mobile application in Latino Americans-Adelgaza Trial. Translational Behavioral Medicine. 2018;8(5):714-23.	*Two brief in-person counseling sessions, daily use of Fitbit Zip (3-axis accelerometer) and Fitbit app, and social media (Facebook) *During the 8-week intervention period, participants were asked to log all food/drinks and calories every day and their weight into the Fitbit app, sync daily steps data that were stored in a Fitbit Zip with the Fitbit app, and interact on Facebook
Gary-Webb TL, Walker EA, Realmuto L, Kamler A, Lukin J, Tyson W, et al. Translation of the National Diabetes Prevention Program to Engage Men in Disadvantaged Neighborhoods in New York City: a description of power up for health. American journal of men's health. 2018:1557988318758788.	*Provided incentive of 6-month parks membership and small incentives throughout to increase motivation, including t-shirts, water bottle, pedometer *Provided $15 for completing baseline and follow-up surveys *Coaches were role-models (black and/or latino, only men) for participants: either through modeling weight loss efforts or professional expertise in health education or fitness training *Statistics on erectile dysfunction and diabetes incorporated in “quick facts” section *Intervention sessions conducted within park and recreations sites that were accessible to participants’ neighborhood and had exercise resources that could help men adhere to physical activity component of the program *Revised curriculum to incorporate photos, examples, and quotes that would appeal to men
Gutierrez J, Devia C, Weiss L, Chantarat T, Ruddock C, Linnell J, et al. Health, community, and spirituality: evaluation of a multicultural faith-based diabetes prevention program. The Diabetes educator. 2014;40(2):214-22.	*FFF includes nutrition education and fitness activities while incorporating Bible-based teachings that encourage healthy lifestyles. *Participants reported statistically significant improvements in knowledge and healthy behaviors from baseline. Statistically significant numbers reported that they ate less fast food and were less likely to overeat at follow-up *FFF demonstrates the potential of faith-based health interventions to address obesity and diabetes risk in high-need communities of color.
Hall D, Lattie E, McCalla J, Saab P. Translation of the diabetes prevention program to ethnic communities in the United States. Journal of Immigrant & Minority Health. 2016;18(2):479-89.	Afro-Americans *Offered sessions in baptist churches *Material for the intervention was tailored to group discussions, some questions were complemented to evoke the discussion *Praying at the beginning of every session Latinos *Bilingual personnel (trainers, diet assistants, educators) *Realization of the program took place in the environment of the latino participants *Information materials were tailored to a low level of health literacy and focused on visualizations (“nutritional traffic light”) Arab Americans *Bilingual personnel (trainers, diet assistants, educators) *Offered pre-interventional education program *Previous focus groups to identify relevant points for the adaption of the program (pa and educational sessions separated in gender, motivating men to participate in meal preparation, including religion) *Every session starts with an Arab wisdom *Cooking lessons are tailored to Arab food *Groups (*n*=10–12) instead of single sessions *Bilingual information
Handley MA, Harleman E, Gonzalez-Mendez E, Stotland NE, Althavale P, Fisher L, et al. Applying the COM-B model to creation of an IT-enabled health coaching and resource linkage program for low-income Latina moms with recent gestational diabetes: the STAR MAMA program. Implementation science : IS. 2016;11(1):73.	Combination of a health literacy-tailored health IT tool for reaching ethnic minority patients with diabetes and DPP content 5 DPP topics in Spanish and English *Physical Activity *Nutrition *Mental Health and Stress *Weight Loss *Glucose Screening Postpartum
Harvey I, Schulz A, Israel B, Sand S, Myrie D, Lockett M, et al. The Healthy Connections project: a community-based participatory research project involving women at risk for diabetes and hypertension. Progress in community health partnerships : research, education, and action. 2009;3(4):287-300.	*“Healthy Connections Advocates” provided screening through House Parties and shared health information and practical support with members of their social networks
Kato S, Ando M, Kondo T, Yoshida Y, Honda H, Maruyama S. Lifestyle intervention using Internet of Things (IoT) for the elderly: a study protocol for a randomized control trial (the BEST-LIFE study). Nagoya J Med Sci. 2018;80(2):175-82.	*Devices loading Internet of things (activity meter, blood pressure (BP) monitor, body weight (BW) scale), Public health nurse guidance by phone, exercise movie *Patients were provided with smartphones programmed with the study-specific application, Bluetooth-enabled activity trackers, Bluetooth-enabled BP monitors, and Bluetooth-enabled BW scales. The measurements recorded in the activity meters, BW scales, and sphygmomanometer will be automatically collected and stored via the Internet *A movie filming a model of optimal exercise will be weekly delivered. It will be changed to step-up/step-down depending on the amount of physical activity in the previous week *Lifestyle guidance was provided by a public health nurse (call center) via a telephone call once a month, and concomitantly, each participant’s behavior modification stage was surveyed
Kim SE, Castro Sweet CM, Gibson E, Madero EN, Rubino B, Morrison J, et al. Evaluation of a digital diabetes prevention program adapted for the Medicaid population: study design and methods for a non-randomized, controlled trial. Contemp Clin Trials Commun. 2018;10:161-8.	*Digital intensive lifestyle intervention based on DPP that includes virtual group support, personalized health coaching, weekly lessons, and digital tracking tools *Participants are placed into small virtual groups with peers *Each group has a private online social network where they can discuss goals, challenges, progress, and provide social support to one another at any time, similar to a private chat board or discussion board. *Users asynchronously complete weekly health education lessons each week. The lessons are available on the digital platform and can be accessed through internet or smartphone. *User communicates with their health coach and receives individualized counseling through private messaging on the platform; coaches also facilitate discussions on the group chat board *Users track meals using digital online tracking tools, track weight loss and physical activity using a wireless weight scale and pedometer, and monitor their engagement and weight loss progress.
Newton RL, Jr., Johnson WD, Larrivee S, Hendrick C, Harris M, Johannsen NM, et al. A randomized community-based exercise training trial in African American men: ARTIIS. Med Sci Sports Exerc. 2019.	*Exercise training intervention for 5 months consisting of 150 min of moderate intensity aerobic activity and two days of resistance training per week, consistent with the current federal physical activity guidelines
Nicolaou M, Vlaar E, van Valkengoed I, Middelkoop B, Stronks K, Nierkens V. Development of a diabetes prevention program for Surinamese South Asians in the Netherlands. Health promotion international. 2013;29(4):680-91.	*Components of DH!AAN intervention focus on improving diet and physical activity, risk perception of the disease, social contexts regarding to support by family and friends, the generation of positive attitudes towards healthy versions of traditional behavior and food and the development of skills like cooking. *Use of an information folder using a “Bollywood” theme: Photos of South Asian individuals, examples and tips on healthy eating using traditional foods, items on yoga, testimonials on healthy eating from a local man and stories about weight loss practices from Bollywood stars. *Integration of basic and specific cultural elements (eating/cooking habits, influence of social environment)
Ruggiero LO, S; Choi, J.K: Community-based translation of the diabetes prevention program’s lifestyle intervention in an underserved Latino population. The Diabetes EDUCATOR 2011, 37(4):564-672.	*Regular community advisory board meetings, inclusion of community members as project staff and interventionists (i.e., chws), and hosting community forums *Program was further tailored and enhanced for this latino community by providing culturally specific information on diabetes risk and providing culturally relevant and language-appropriate program and supplemental educational material *Implementation of the groups was designed to minimize the impact of barriers to participation, such as education and literacy levels, language, income, transportation, and lack of medical coverage *To address language and literacy barriers, the group sessions were conducted in spanish, and all participant program materials were provided in spanish *In addition to receiving program session materials, participants were provided with supplemental culturally appropriate educational materials (e.g., recipe book, national diabetes education program materials), self-monitoring tools (e.g., personalized weight chart), a pedometer, a body weight scale, and measuring cups
Siddiqui F, Koivula RW, Kurbasic A, Lindblad U, Nilsson PM, Bennet L. Physical activity in a randomized culturally adapted lifestyle intervention. Am J Prev Med. 2018;55(2):187-96.	*In addition to the verbal instructions by an Arabic-speaking nurse, participants were also provided with written instructions. Participants were instructed to wear the device continuously for a total of 10 complete days *The intervention was composed of seven group sessions, including one cooking class, and addressed self-empowerment, cultural and social barriers to lifestyle change, diet, and PA habits *Health coaches organized and led the sessions, but participants were encouraged to actively participate in the discussions *Participants were asked to identify barriers to being physically active and to develop an action plan for overcoming such obstacles. They were motivated to incorporate PA in daily life, such as walking or using stairs instead of riding a bus or taking the elevator *Gender differences and cultural barriers to PA were discussed *Participants received information on available PA centers, including a “women only” swimming hall, and gyms in the area *Participants were offered financial assistance to buy shoes and clothing for PA or admission to a PA center
Vincent D, McEwen MM, Hepworth JT, Stump CS. The effects of a community-based, culturally tailored diabetes prevention intervention for high-risk adults of Mexican descent. The Diabetes educator. 2014;40(2):202-13.	IG Culturally tailored messages for promoting healthful eating and for increasing physical activity. Cultural tailoring included using an educational fotonovela (story with photographs and small dialogue bubbles), offering the intervention and all materials in Spanish and English, using culturally acceptable exercise strategies (e.g., walking, dancing), providing cooking demonstrations of low-fat traditional Mexican American foods, and facilitating group meal sharing. CG Educational sessions giving general information on health promotion and disease prevention.
Walker EA, Weiss L, Gary-Webb TL, Realmuto L, Kamler A, Ravenell J, et al. Power up for health: pilot study outcomes of a diabetes prevention program for men from disadvantaged neighborhoods. American Journal of men’s health. 2018;12(4):989-97.	*Curriculum adapted to better engage men from disadvantaged, urban neighborhoods. Modifications to the ndpp curriculum were made by the research team in an iterative manner under the guidance of an advisory panel of experts in men’s health promotion, male community leaders, and the power up for health coaches *Curriculum was facilitated by male lifestyle coaches who had received training as an ndpp coach and also trained in the delivery of the power up for health curriculum *If participating men missed a session, however, by protocol the coaches proactively offered a telephone make-up of that session to each participant to be done after they received the session materials by email or by mail from study staff. These telephone make-up sessions were delivered as individual topic sessions (i.e., not multiple missed sessions grouped in one phone call) and independent of the in-person sessions
Whittemore R, Rosenberg A, Gilmore L, Withey M, Breault A. implementation of a diabetes prevention program in public housing communities. Public Health Nursing. 2014;31(4):317-26.	*Homecare nurses delivered an ADPP at public housing communities to adults at-risk for T2DM *DPP was adapted after focus groups with stakeholders and residents of the community

*ADPP* adapted Diabetes Prevention Program, *BP* blood pressure, *BW* body weight, *CHW* community health worker, *CG* control group, *DPP* Diabetes Prevention Program, *IG* intervention group, *LSES* low socioeconomic status, *NDPP* National Diabetes Prevention Program, *PA* physical activity, *TA* technical approach, *RCT* randomized controlled trial

### Adapted diabetes prevention programs

We identified 21 studies that used any kind of ADPP (in 12 studies, it was the main focus [21–32]). Most studies adapted the DPP [9], which is a lifestyle intervention designed in 2002 by the DPP Research Group. It is based on a 16-session core curriculum with sessions such as Healthy Eating, Being Active, and Problem Solving. Other programs such as the National Diabetes Education Program (NDEP) [33] or the National Diabetes Prevention Program (NDPP) [34] were also used as a curriculum base for some of the included studies (e.g., NDEP [35] and NDPP [32]). The included studies (*n*=21) targeted mostly migrants (*n*= 9) and ethnic groups (*n*=6). Mostly, the adaptation of the DPPs was based on barriers or facilitating factors to participate in a preventive program, e.g., language barriers, economic factors, or religious/cultural background. Some studies (for example [22, 25, 31]) were adapted for cultural/religious backgrounds, including language adaptation. Other studies focused on community translation and financial aspects.

One example of an ADPP is the study conducted by Nicolaou et al. [28]. They described a lifestyle intervention based on a DPP targeting Surinamese men and women from South Asia living in the Netherlands. The program was culturally adapted; e.g., they had dietitians who were familiar with Surinamese South Asian dietary habits and provided cooking classes to adjust traditional dishes. Furthermore, they provide family sessions in home settings to integrate family in achieving dietary goals. Additionally, they used role models from favorite Bollywood movies in an information folder with examples and tips on healthy eating using traditional foods, yoga, and stories about weight loss practices from Bollywood stars. Cultural adaptation was also achieved with respect to physical activity by recommending 30 min of daily yoga and Bollywood-like dancing using motivational interviewing [28].

### Community health workers

We identified five studies, which used CHWs, three of which focused on CHWs [35–38]. Blanks et al. [35] and Harvey et al. [37] both used CHWs in African American settings for preventive interventions. In general, CHWs were trained in T2DM prevention and had the same ethnic and social background as the participants. Blanks et al. focused on prediabetic African American women. The aim was to increase participant knowledge related to the complex of nutrition, physical activity, and health literacy and therefore have a positive impact on increasing awareness of diabetes risks and improved access to healthcare [35]. Harvey et al. targeted African Americans and Latinos (88.5% women) at risk of diabetes and hypertension in house parties where health workers or health connections advocates provided screening and shared health information and practical support with members of their social networks [37].

### Technological approaches

Another communication strategy was an approach using any kind of technical device. Overall, nine studies focused on TA in combination with an ADPP [20, 36, 39–45], but two used a TA alone [39, 40].

Fischer et al. [36] sent bilingual (English/Spanish) text messages relating to nutrition, physical activity, and motivation. The intervention group could additionally join the DPP classes. Handley et al. [43] also sent text messages and used weekly phone calls plus live, tailored callback health coaching.

The kiosk system used by Bolin et al. [39] is a bilingual (English/Spanish) diabetes education kiosk system (Diosk) that provides information on different topics such as what “diabetic” means, preventing diabetes, meal planning, and exercise. Kiosk systems use touch-screen displays at waist level. They were placed in low-income pharmacies, federally qualified healthcare centers, and community arts centers. The Diosks not only provide on-demand information to the user but also collect general user frequencies and the frequencies of the topics accessed.

Fontil et al. [41] used a bilingual (English/Spanish) digital health program for low-income prediabetic participants. IT support for the first use of the program, especially for people with low computer capabilities as well as an informational event adapted to people with a low reading level were provided prior to the program use.

## Discussion

We identified different communication strategies for the prevention of T2DM in vulnerable groups. All but two studies were conducted in the USA and therefore targeted mostly ethnic groups (African Americans) or migrants. Most communication strategies used TAs or adapted existing programs such as the DPP for language and cultural background. TAs differed most within the categories, because every identified study used a different approach (videos, SMS, kiosk systems, and digital health program). The other categories were more similar among the studies, but they may have, for example, adapted for different barriers for different vulnerable groups.

It seems necessary to identify common barriers and facilitating factors in T2DM prevention to develop communication strategies for vulnerable groups. Some of the identified studies initially searched for barriers and facilitating factors or gained this information through focus groups. Therefore, some of the communication strategies had already been adapted to known barriers [30, 39] such as language (e.g., adaptation to bilingualism) or cultural factors.

The communication strategies often apply to just one vulnerable group, and there are just a few that overlap, such as low-income migrants. Especially, religious and cultural factors seem to require different approaches for each vulnerable group [46]. Furthermore, some approaches focused on only one gender [24, 35]. Therefore, communication strategies should be tailored for one vulnerable group and/or gender and adapted to all known barriers.

One important known facilitating factor is family and friends [47]. None of the identified studies adapted their approaches with respect to this factor. For example, it is conceivable to include family members in cooking or exercise classes.

Most studies used more than one communication strategy; just a few used only one. There are no data on the effectiveness of one strategy compared with mixed strategies regarding the willingness to participate or the outcome itself. It might be necessary to use a mixed strategy to adapt a preventive program for some of the known barriers, for example, to use a combination of a CHW strategy (a female CHW) and an ADPP strategy (language and cultural adaptation for migrant women). Perhaps, it is required to analyze whether all approaches could be used for each vulnerable group. For example, TAs may need a special adaptation for older people. Yet, recent studies show a high adherence to TA in vulnerable groups [48].

There are systematic reviews focusing on single preventive or therapeutic approaches like CHW or community health center-based interventions [49] on diabetes in the general population. This scoping review adds a detailed list of communication strategies in the prevention of type 2 diabetes and gestational diabetes in vulnerable groups to the literature. In conjunction with recently described barriers and facilitating factors in diabetes prevention in vulnerable groups [50]; therefore, future preventive programs can benefit from these findings or already existing programs could be adapted.

### Limitations

As mentioned before, this review is part of the national awareness and prevention strategy on diabetes in Germany conducted by the Federal Center for Health Education and the Federal Ministry of Health. For this reason, we had to limit the publication date of included studies because we had a narrow time frame for completing this scoping review. However, there might be too many differences in communication strategies arising from increasing digitization. Sometimes, it was difficult to assign groups to either ethnic groups or migrants. Therefore, there may have been incorrect assignments to the vulnerable groups. Since these two groups have a lot of overlap in barriers and facilitating factors to join a preventive program, we do not expect these potentially incorrect assignments to affect the quality of this study. This overlap arises from the fact that some ethnic groups may have originally been migrants in another generation. Due to this overlap, it would be conceivable to address both groups together in preventive measures. Furthermore, although we stated the main focus regarding the communication strategy of each study, we could not know whether this was the intended focus of the studies. Due to the aim of this scoping review, we assume that this has no impact on the quality of this study.

### Further directions

Since digitalization has become increasingly important over the last few years, mixed approaches including TAs versus TA only should be analyzed regarding effectiveness. Furthermore, the effectiveness of different TAs should be analyzed.

## Conclusion

We identified three different categories of communication strategies for the prevention of T2DM in vulnerable groups. Some communication strategies are already adapted to barriers and facilitating factors to increase participation and motivation. Since most studies report communication strategies only for the vulnerable group, it seems necessary to address each vulnerable group separately.

Since this project was commissioned by the Federal Center for Health Education in Germany as part of the national education and communication strategy on diabetes mellitus, the results of our and other projects were summarized and are currently used to generate a national education and communication strategy. The implementation of this strategy is the responsibility of the Federal Center for Health Education in Germany.

## Supplementary Information

**Additional file 1 **: **Supplement 1**. Search strategies.

**Additional file 2 **: **Supplement 2**. List of studies excluded in full-text screening.

**Additional file 3: Supplement 3.** Preferred Reporting Items for Systematic reviews and Meta-Analyses extension for Scoping Reviews (PRISMA-ScR) Checklist.

**Additional file 4 **: **Supplement 4**. List of WHO stratum A countries.

## Data Availability

Not applicable.

## Abbreviations

ADPP: Adapted Diabetes Prevention Program
CHW: Community health worker
DPP: Diabetes Prevention Program
GDM: Gestational diabetes
NDEP: National Diabetes Education Program
NDPP: National Diabetes Prevention Program
PPC: Population, Concept, Context
RCT: Randomized controlled trial
T2DM: Type 2 diabetes mellitus
TA: Technical approach
WHO: World Health Organization

## References

[1] Ogurtsova K (2017). dRFJ, Huang Y, Linnenkamp U, Guariguata L, Cho NH, Cavan D, Shaw JE, Makaroff LE: IDF diabetes atlas: global estimates for the prevalence of diabetes for 2015 and 2040. Diabetes Res Clin Pract.

[2] Gabbe SG, Persson B, Buchanan TA, Catalano PA, Damm P, Dyer AR, Ad L, Hod M, Kitzmiler JL, Lowe LP, HD MI, Oats JJ, Omori Y, Schmidt MI, International Association of Diabetes and Pregnancy Study Groups Consensus Panel MB (2010). International association of diabetes and pregnancy study groups recommendations on the diagnosis and classification of hyperglycemia in pregnancy. Diabetes Care.

[3] Bommer CSV, Heesemann E, Manne-Goehler J, Atun R, Bärnighausen T, Davies J, Vollmer S (2018). Global economic burden of diabetes in adults: projections from 2015 to 2030. Diabetes Care.

[4] Association AD (2018). 5. Prevention or delay of type 2 diabetes: standards of medical care in diabetes-2018. Diabetes Care.

[5] All Parliamentary Group for Diabetes and Diabetes UK (2006). Diabetes and the disadvantaged: reducing health inequalities in the UK.

[6] Meeks KAC, Adeyemo A, Beune EJ, Modesti PA, Stronks K, Zafarmand MH (2016). Disparities in type 2 diabetes prevalence among ethnic minority groups resident in Europe: a systematic review and meta-analysis. Intern Emerg Med..

[7] Cefalu WTBJ, Tuomilehto J, Fleming GA, Ferrannini E, Gerstein HC, Bennett PH, Ramachandran A, Raz I, Rosenstock J, Kahn SE (2016). Update and next steps for real-world translation of interventions for type 2 diabetes prevention: reflections from a diabetes care editors’ expert forum. Diabetes Care.

[8] Diabetes Prevention Program (DPP) [https://www.niddk.nih.gov/about-niddk/research-areas/diabetes/diabetes-prevention-program-dpp]. Accessed 03 Sep 2021.

[9] Diabetes Prevention Program (DPP) Research Group, The Diabetes Prevention Program (DPP): description of lifestyle intervention. Diabetes Care. 2002;25(12):2165–71. 10.2337/diacare.25.12.2165 PMC1282458 12453955

[10] Kyrou I, Tsigos C, Mavrogianni C, Cardon G, Van Stappen V, Latomme J, Kivelä J, Wikström K, Tsochev K, Nanasi A (2020). Sociodemographic and lifestyle-related risk factors for identifying vulnerable groups for type 2 diabetes: a narrative review with emphasis on data from Europe. BMC Endocr Disord.

[11] Kivelä J, Wikström K, Virtanen E, Georgoulis M, Cardon G, Civeira F, Iotova V, Karuranga E, Ko W, Liatis S (2020). Obtaining evidence base for the development of Feel4Diabetes intervention to prevent type 2 diabetes - a narrative literature review. BMC Endocr Disord.

[12] Breuing J, Pieper D, Neuhaus AL, Heß S, Lütkemeier L, Haas F, Spiller M, Graf C (2018). Barriers and facilitating factors in the prevention of diabetes type II and gestational diabetes in vulnerable groups: protocol for a scoping review. Syst Rev.

[13] Breuing J, Graf C, Neuhaus AL, Heß S, Lütkemeier L, Haas F, Spiller M, Pieper D (2019). Communication strategies in the prevention of type 2 and gestational diabetes in vulnerable groups: protocol for a scoping review. Syst Rev.

[14] Arksey HaOM L (2005). Scoping studies: towards a methodological framework. Int J Soc Res Methodol.

[15] The Joanna Briggs Institute, Joanna Briggs Institute Reviewers’ Manual: 2015 edition / Supplement: Methodology for JBI Scoping Reviews, The Joanna Briggs Institute. https://nursing.lsuhsc.edu/JBI/docs/ReviewersManuals/Scoping-.pdf.

[16] Tricco AC, Lillie E, Zarin W, O'Brien KK, Colquhoun H, Levac D, Moher D, Peters MDJ, Horsley T, Weeks L, Hempel S, Akl EA, Chang C, McGowan J, Stewart L, Hartling L, Aldcroft A, Wilson MG, Garritty C, Lewin S, Godfrey CM, Macdonald MT, Langlois EV, Soares-Weiser K, Moriarty J, Clifford T, Tunçalp Ö, Straus SE (2018). PRISMA Extension for Scoping Reviews (PRISMA-ScR): checklist and explanation. Ann Intern Med.

[17] List of Member States by WHO region and mortality stratum [http://www.who.int/whr/2003/en/member_states_182-184_en.pdf]. Accessed 03 Sep 2021.

[18] Lewis V, Larson BK, McClurg A, Goldman Boswell R, Fisher ES (2012). The promise and peril of accountable care for vulnerable populations: a framework for overcoming obstacles. Health Aff.

[19] McGowan J, Sampson M, Salzwedel DM, Cogo E, Foerster V, Lefebvre C (2016). PRESS Peer Review of Electronic Search Strategies: 2015 Guideline Explanation and Elaboration (PRESS E&E). J Clin Epidemiol.

[20] Bender MS, Cooper BA, Flowersc E, Mae R, Araia S (2018). Filipinos fit and trim - a feasible and efficacious DPP-based intervention trial. Contemp Clin Trials Commun.

[21] Borelli MR, Riden HE, Bang H, Schenker MB (2018). Protocol for a cluster randomized controlled trial to study the effectiveness of an obesity and diabetes intervention (PASOS) in an immigrant farmworker population. BMC Public Health.

[22] Castro-Rivas E, Boutin-Foster C, Milan M, Kanna B (2014). “Es como uno bomba de tiempo [It’s like a time bomb]”: a qualitative analysis of perceptions of diabetes among first-degree relatives of latino patients with diabetes. Diabetes Spectr.

[23] Ford AF, Reddick KB, Browne MC, Robins A, Thomas SB, Quinn SC (2009). Beyond the Cathedral: building trust to engage the African American community in health promotion and disease prevention. Health Promot Pract.

[24] Gary-Webb TL, Walker EA, Realmuto L, Kamler A, Lukin J, Tyson W, Carrasquillo O, Weiss L (2018). Translation of the national diabetes prevention program to engage men in disadvantaged neighborhoods in New York City: a description of power up for health. Am J Mens Health.

[25] Gutierrez J, Devia C, Weiss L, Chantarat T, Ruddock C, Linnell J, Golub M, Godfrey L, Rosen R, Calman N (2014). Health, community, and spirituality - evaluation of a multicultural faith-based diabetes prevention program. Diabetes Educ.

[26] Hall DL, Lattie EG, McCalla JR, Saab PG (2016). Translation of the diabetes prevention program to ethnic communities in the United States. J Immigr Minor Health.

[27] Newton RLJ, WD Johnson,; Larrivee, S.; Hendrick, C.; Harris, M.; Johannsen, N.M.; Swift, D.L.; Hsia, D.S.; Church, T.S.: A randomized community-based exercise training trial in African American men: ARTIIS. Med Sci Sports Exerc 2019, N.R.(N.R.):1-44. 10.1249/MSS.0000000000002149 PMC6986197 31939911

[28] Nicolaou M, Vlaar E, van Valkengoed I, Middelkoop B, Stronks K, Nierkens V (2013). Development of a diabetes prevention program for Surinamese South Asians in the Netherlands. Health Promot Int.

[29] Ruggiero L, Oros S, Choi JK (2011). Community-based translation of the diabetes prevention program’s lifestyle intervention in an underserved latino population. Diabetes Educ.

[30] Siddiqui F, Koivula RW, Kurbasic A, Lindblad U, Nilsson PM, Bennet L (2018). Physical activity in a randomized culturally adapted lifestyle intervention. Am J Prev Med.

[31] Vincent D, McEwen MM, Hepworth JT, Stump CS (2014). The effects of a community-based, culturally tailored diabetes prevention intervention for high-risk adults of mexican descent. Diabetes Educ.

[32] Walker EA, Weiss L, Gary-Webb TL, Realmuto L, Kamler A, Ravenell J, Tejeda C, Lukin J, Schechter CB (2018). Power up for health: pilot study outcomes of a diabetes prevention program for men from disadvantaged neighborhoods. Am J Mens Health.

[33] National Diabetes Education Program [https://www.niddk.nih.gov/health-information/communication-programs/ndep/about-national-diabetes-education-program]. Accessed 23 Oct 2019.

[34] Centers for Disease Control and Prevention, National Diabetes Prevention Program. https://www.cdc.gov/diabetes/prevention/index.html. Accessed 23 Oct 2019.

[35] Blanks SH, Treadwell H, Bazzell A, Graves W, Osaji O, Dean J, McLawhorn JT, Stroud JL (2016). Community engaged lifestyle modification research: engaging diabetic and prediabetic African American women in community-based interventions. J Obes.

[36] Fischer HH, Fischer IP, Pereira RI, Furniss AL, Rozwadowski JM, Moore SL, Durfee MJ, Raghunath SG, Tsai AG, Edward P (2016). Havranek1: Text message support for weight loss in patients with prediabetes: a randomized clinical trial. Diabetes Care.

[37] Harvey I, Schulz A, Israel B, Sand S, Myrie D, Lockett M, Weir S, Hill Y (2009). The healthy connections project: a community-based participatory research project involving women at risk for diabetes and hypertension. Prog Community Health Partnersh.

[38] Whittemore R, Rosenberg A, Gilmore L, Withey M, Breault A (2013). Implementation of a diabetes prevention program in public housing communities. Public Health Nurs.

[39] Bolin JN, Ory MG, Wilson AD, Salge L (2013). Diabetes education kiosks in a latino community. Diabetes Educ.

[40] Chang M-W, Brown R, Nitzke S (2017). Results and lessons learned from a prevention of weight gain program for low-income overweight and obese young mothers: mothers in motion. BMC Public Health.

[41] Fontil V, McDermott K, Tieu L, Rios C, Gibson E, Sweet CC, Payne M, Lyles CR (2016). Adaptation and feasibility study of a digital health program to prevent diabetes among low-income patients: results from a partnership between a digital health company and an Academic Research Team. J Diabetes Res.

[42] Fukuoka Y, Vittinghoff E, Hooper J (2018). A weight loss intervention using a commercial mobile application in Latino Americans—Adelgaza Trial. Transl Behav Med.

[43] Handley MA, Harleman E, Gonzalez-Mendez E, Stotland NE, Althavale P, Fisher L, Martinez D, Ko J, Sausjord I, Rios C (2016). Applying the COM-B model to creation of an IT-enabled health coaching and resource linkage program for low-income Latina moms with recent gestational diabetes: the STAR MAMA program. Implement Sci.

[44] Kato S, Ando M, Kondo T, Yoshida Y, Honda H, Maruyama S (2018). Lifestyle intervention using Internet of Things (IoT) for the elderly: a study protocol for a randomized control trial (the BEST-LIFE study). Nagoya J Med Sci.

[45] Kim SE, Sweet CMC, Gibsonb E, Maderob EN, Rubinoc B, Morrisonc J (2018). Evaluation of a digital diabetes prevention program adapted for the Medicaid population: study design and methods for a non-randomized, controlled trial. Contemp Clin Trials Commun..

[46] Islam NS, Tandon D, Mukherji R, Tanner M, Ghosh K, Alam G, Haq M, Rey MJ, Trinh-Shevrin C (2012). Understanding barriers to and facilitators of diabetes control and prevention in the New York City Bangladeshi community: a mixed-methods approach. Dermatol Res Pract.

[47] Hempler NF, Nicic S, Ewers B, Willaing B (2015). Dietary education must fit into everyday life: a qualitative study of people with a Pakistani background and type 2 diabetes. Patient Prefer Adherence.

[48] Arsenijevic J, Tummers L, Bosma N (2020). Adherence to electronic health tools among vulnerable groups: systematic literature review and meta-analysis. J Med Internet Res.

[49] Han HR, McKenna S, Nkimbeng M, Wilson P, Rives S, Ajomagberin O, Alkawaldeh M, Grunstra K, Maruthur N, Sharps P (2019). A systematic review of community health center based interventions for people with diabetes. J Community Health.

[50] Breuing J, Pieper D, Neuhaus AL, Heß S, Lütkemeier L, Haas F, Spiller M, Graf C (2020). Barriers and facilitating factors in the prevention of diabetes type 2 and gestational diabetes in vulnerable groups: a scoping review. PLoS One.

