# Supplementary material for: Communication strategies in the prevention of type 2 diabetes and gestational diabetes in vulnerable groups: a scoping review
Source: Syst Rev. 2021 Nov 24;10:301. doi: 10.1186/s13643-021-01846-8 (PMC8611985; doi:10.1186/s13643-021-01846-8)
Supplement: Supplementary file 2 — Additional file 2 : Supplement 2. List of studies excluded in full-text screening. [file 13643_2021_1846_MOESM2_ESM.docx]

Supplement 2 Table 7: Excluded studies

| **Initial full text screening** | **nft** | **P1** | **P2** | **Concept 1** | **Concept 2** | **Context** |
| --- | --- | --- | --- | --- | --- | --- |
| Allen NA, Fain JA, Braun B, Chipkin SR. Continuous glucose monitoring in non-insulin-using individuals with type 2 diabetes: acceptability, feasibility, and teaching opportunities. Diabetes technology & therapeutics. 2009;11(3):151-8. |  |  | x |  |  |  |
| Allen NA, Melkus GD, Chyun DA. Physiological and behavioral factors related to physical activity in black women with type 2 diabetes mellitus. Journal of transcultural nursing: official journal of the Transcultural Nursing Society. 2011;22(4):376-85. |  |  |  | x |  |  |
| Alva ML, Samuel-Hodge CD, Porterfield D, Thomas T, Leeman J. A Feasibility Study of Supply and Demand for Diabetes Prevention Programs in North Carolina. Preventing chronic disease. 2017;14. |  |  | x |  |  |  |
| Alzubaidi H, Mc Namara K, Browning C. Time to question diabetes self-management support for Arabic-speaking migrants: exploring a new model of care. Diabetic medicine : a journal of the British Diabetic Association. 2017;34(3):348-55. |  |  |  |  | x |  |
| Alzubaidi H, Mc Namara K, Browning C, Marriott J. Barriers and enablers to healthcare access and use among Arabic-speaking and Caucasian English-speaking patients with type 2 diabetes mellitus: a qualitative comparative study. BMJ open. 2015;5(11):e008687. |  |  |  |  | x |  |
| Alzubaidi H, Mc Narmara K, Kilmartin GM, Kilmartin JF, Marriott J. The relationships between illness and treatment perceptions with adherence to diabetes self-care: A comparison between Arabic-speaking migrants and Caucasian English-speaking patients. Diabetes research and clinical practice. 2015;110(2):208-17. |  |  |  |  | x |  |
| Amendezo E, Walker Timothy D, Karamuka V, Robinson B, Kavabushi P, Ntirenganya C, et al. Effects of a lifestyle education program on glycemic control among patients with diabetes at Kigali University Hospital, Rwanda: A randomized controlled trial. Diabetes research and clinical practice. 2017;126:129-37. |  |  |  |  |  | x |
| Amirehsani KA. Mexican Americans with type 2 diabetes in an emerging Latino community: Evaluation of health disparity factors and interventions. Home Health Care Management & Practice. 2010;22(7):470-8. |  |  |  |  | x |  |
| Andersen E, Høstmark AT, Holme I, Anderssen SA. Intervention effects on physical activity and insulin levels in men of Pakistani origin living in Oslo: A randomised controlled trial. Journal of immigrant and minority health. 2013;15(1):101-10. |  |  |  |  | x |  |
| Anderson BJ. Can we break down barriers to excellent diabetes healthcare for teenagers with diabetes? Diabetes Management. 2012;2(6):471-3. | x |  |  |  |  |  |
| Anderson DR, Christison-Lagay J, Procter-Gray E. Self-management goal setting in a Community Health Center: The impact of goal attainment on diabetes outcomes. Diabetes Spectrum. 2010;23(2):97-105. |  |  |  |  |  |  |
| Anwar A, Salih A, Masson E, Allen B, Wilkinson L, Lindow SW. The effect of pre-pregnancy counselling for women with pre-gestational diabetes on maternal health status. European journal of obstetrics, gynecology, and reproductive biology. 2011;155(2):137-9. |  |  | x |  |  |  |
| Aponte J, Nokes KM. Electronic health literacy of older Hispanics with diabetes. Health promotion international. 2017;32(3):482-9. |  |  |  |  |  |  |
| Attridge M, Creamer J, Ramsden M, Cannings-John R, Hawthorne K. Culturally appropriate health education for people in ethnic minority groups with type 2 diabetes mellitus. The Cochrane database of systematic reviews. 2014(9):Cd006424. |  |  |  |  |  |  |
| Aujla N, Stone MA, Taub N, Davies MJ, Khunti K. Identifying people with type 2 diabetes and those at risk: Lessons from the Measure Your Waist (MY-WAIST) mixed-methods study in UK primary care. Primary care diabetes. 2013;7(4):261-7. |  |  |  |  |  |  |
| Avdal EU, Kizilci S, Demirel N. The Effects of Web-Based Diabetes Education on Diabetes Care Results A Randomized Control Study. Cin-Computers Informatics Nursing. 2011;29(2):101-6. | x |  |  |  |  |  |
| Badrick E, Hull S, Mathur R, Shajahan S, Boomla K, Bremner S, et al. Health equity audits in general practice: A strategy to reduce health inequalities. Primary Health Care Research and Development. 2014;15(1):80-95. |  |  |  |  |  |  |
| Baig AA, Benitez A, Locklin CA, Gao Y, Lee SM, Quinn MT, et al. Picture Good Health: A Church-Based Self-Management Intervention Among Latino Adults with Diabetes. Journal of general internal medicine. 2015;30(10):1481-90. |  |  |  |  | x |  |
| Baig AA, Locklin CA, Foley E, Ewigman B, Meltzer DO, Huang ES. THE ASSOCIATION OF ENGLISH ABILITY AND GLYCEMIC CONTROL AMONG LATINOS WITH DIABETES. Ethnicity & disease. 2014;24(1):28-34. |  |  |  |  | x |  |
| Baig AA, Wilkes AE, Davis AM, Peek ME, Huang ES, Bell DS, et al. The use of quality improvement and health information technology approaches to improve diabetes outcomes in African American and Hispanic patients. Medical care research and review: MCRR. 2010;67(5 Suppl):163s-97s. |  |  |  |  | x |  |
| Balasanthiran A, O'Shea T, Moodambail A, Woodcock T, Poots AJ, Stacy M, et al. Type 2 diabetes in children and young adults in East London: an alarmingly high prevalence. Practical Diabetes. 2012;29(5):193-8. |  |  | x |  |  |  |
| Baldoni NR, Aquino JA, Sanches-Giraud C, Di Lorenzo Oliveira C, de Figueiredo RC, Cardoso CS, et al. Collective empowerment strategies for patients with Diabetes Mellitus: A systematic review and meta-analysis. Primary care diabetes. 2017;11(2):201-11. |  |  | x |  |  |  |
| Baptiste-Roberts K, Bronner Y, Nicholson WK. Adoption of a Healthy Lifestyle Following Gestational Diabetes Mellitus. Current Nutrition Reports. 2015;4(3):259-64. |  |  | x |  |  |  |
| Barko R, Corbett CF, Allen CB, Shultz JA. Perceptions of diabetes symptoms and self-management strategies: a cross-cultural comparison. Journal of transcultural nursing: official journal of the Transcultural Nursing Society. 2011;22(3):274-81. |  |  |  |  | x |  |
| Barnard KD, Holt RIG, Dyson PA, Cummings MH, Kanumilli N, O'Neill S, et al. Could the discrepancy in perceived emotional care received and provided be a barrier to active diabetes selfmanagement? Insights from the second diabetes attitudes, wishes and needs (DAWN2) study. Diabetes care. 2016;39(2):e20-e1. |  |  | x |  |  |  |
| Barnard LS, Wexler DJ, DeWalt D, Berkowitz SA. Material need support interventions for diabetes prevention and control: a systematic review. Current diabetes reports. 2015;15(2):574. |  |  | x |  |  |  |
| Barron E, Clark R, Hewings R, Smith J, Valabhji J. Progress of the Healthier You: NHS Diabetes Prevention Programme: referrals, uptake and participant characteristics. Diabetic medicine : a journal of the British Diabetic Association. 2018;35(4):513-8. |  |  |  |  | x |  |
| Barry Hultquist T, Brown SG, Geske J, Kaiser KL, Waibel-Rycek D. Partnering With Community-Dwelling Individuals With Diabetes for Health Behavior Change Using Action Plans: An Innovation in Health Professionals Education and Practice. Health promotion practice. 2015;16(6):906-15. |  |  | x |  |  |  |
| Bartlam B, Rathod T, Rowlands G, Protheroe J. Lay Health Trainers Supporting Self-Management amongst Those with Low Heath Literacy and Diabetes: Lessons from a Mixed Methods Pilot, Feasibility Study. Journal of diabetes research. 2016;2016:4723636. |  |  | x |  |  |  |
| Basilio CD, Kwan VSY, Towers MJ. Culture and risk assessments: Why Latino Americans perceive greater risk for diabetes. Cultural Diversity and Ethnic Minority Psychology. 2016;22(1):104-13. |  |  |  | x |  |  |
| Bauer C, Graf C, Platschek AM, Struder HK, Ferrari N. Reasons, Motivational Factors, and Perceived Personal Barriers to Engagement in Physical Activity During Pregnancy Vary Within the BMI Classes: The Prenatal Prevention Project Germany. Journal of physical activity & health. 2018;15(3):204-11. |  |  |  |  |  |  |
| Benitez TJ, Cherrington AL, Joseph RP, Keller C, Marcus B, Meneses K, et al. Using Web-Based Technology to Promote Physical Activity in Latinas: Results of the Muevete Alabama Pilot Study. Computers, informatics, nursing : CIN. 2015;33(7):315-24. |  |  |  |  |  |  |
| Bennet L. Ethnic and cultural aspects of type 2 diabetes. Lakartidningen. 2018;115. |  |  |  |  | x |  |
| Bernstein AM, Rudd N, Gendy G, Moffett K, Adams J, Steele S, et al. Beliefs About Preventive Care, Individual Health, and Lifestyle Change Among Low-Income African American Women at Risk for Diabetes. Holistic Nursing Practice. 2014;28(1):24-30. |  |  |  |  | x |  |
| Berry D, Colindres M, Sanchez-Lugo L, Sanchez M, Neal M, Smith-Miller C. Adapting, Feasibility Testing, and Pilot Testing a Weight Management Intervention for Recently Immigrated Spanish-Speaking Women and Their 2- to 4-Year-Old Children. Hispanic Health Care International. 2011;9(4):186-93. |  |  | x |  |  |  |
| Bertero C, Hjelm K. Social support as described by foreign-born persons diagnosed with type 2 diabetes mellitus and living in Sweden. Nursing & health sciences. 2010;12(4):507-14. |  |  |  |  | x |  |
| Betancourt JR, Duong JV, Bondaryk MR. Strategies to reduce diabetes disparities: an update. Current diabetes reports. 2012;12(6):762-8. |  |  |  | x |  |  |
| Betancourt RM, Degnan KO, Long JA. Racial differences in glucose control among patients with type 2 diabetes: a survey on dietary temptations, coping, and trust in physicians. Ethnicity & disease. 2013;23(4):409-14. |  |  | x |  |  |  |
| Betzlbacher AF, Grady K, Savas L, Cotterill S, Boaden R, Summers L, et al. Behaviour change among people with impaired glucose tolerance: Comparison of telephone-based and face-to-face advice. Journal of health services research & policy. 2013;18(1 Suppl):2-6. |  |  | x |  |  |  |
| Beverly EA, Ganda OP, Ritholz MD, Lee Y, Brooks KM, Lewis-Schroeder NF, et al. Look who's (not) talking: diabetic patients' willingness to discuss self-care with physicians. Diabetes care. 2012;35(7):1466-72. |  |  | x |  |  |  |
| Beverly EA, Wray LA, LaCoe CL, Gabbay RA. Listening to older adults' values and preferences for type 2 diabetes care: A qualitative study. Diabetes Spectrum. 2014;27(1):44-9. |  |  |  |  | x |  |
| Beyerlein A, Koller D, Ziegler AG, Lack N, Maier W. Does charge-free screening improve detection of gestational diabetes in women from deprived areas: a cross-sectional study. BMC pregnancy and childbirth. 2016;16:266. |  |  | x |  |  |  |
| Bhattacharya G. Self-management of type 2 diabetes among African Americans in the Arkansas Delta: a strengths perspective in social-cultural context. Journal of health care for the poor and underserved. 2012;23(1):161-78. |  |  |  |  | x |  |
| Bhattacharya G. Spirituality and Type 2 Diabetes Self-Management Among African Americans in the Arkansas Delta. Journal of Social Service Research. 2013;39(4):469-82. |  |  |  |  | x |  |
| Biddle SJ, Edwardson CL, Wilmot EG, Yates T, Gorely T, Bodicoat DH, et al. A Randomised Controlled Trial to Reduce Sedentary Time in Young Adults at Risk of Type 2 Diabetes Mellitus: Project STAND (Sedentary Time ANd Diabetes). PloS one. 2015;10(12):e0143398. |  |  | x |  |  |  |
| Biyikli Gultekin E. Difficulties in health care for female Turkish immigrants with type 2 diabetes: a qualitative study in Vienna. Wiener klinische Wochenschrift. 2017;129(9-10):337-44. |  |  |  | x |  |  |
| Blanks SH, Treadwell H, Bazzell A, Graves W, Osaji O, Dean J, et al. Community Engaged Lifestyle Modification Research: Engaging Diabetic and Prediabetic African American Women in Community-Based Interventions. Journal of obesity. 2016;2016:3609289. |  |  |  |  | x |  |
| Bleich SN, Barry CL, Gary-Webb TL, Herring BJ. Reducing sugar-sweetened beverage consumption by providing caloric information: how Black adolescents alter their purchases and whether the effects persist. American journal of public health. 2014;104(12):2417-24. |  |  | x |  |  |  |
| Block G, Azar KMJ, Romanelli RJ, Block TJ, Hopkins D, Carpenter HA, et al. Diabetes Prevention and Weight Loss with a Fully Automated Behavioral Intervention by Email, Web, and Mobile Phone: A Randomized Controlled Trial Among Persons with Prediabetes. Journal of medical Internet research. 2015;17(10). |  |  | x |  |  |  |
| Bobo N, Shantz S, Kaufman FR, Kollipara S. Lowering risk for type 2 diabetes in high-risk youth. American Journal of Health Education. 2009;40(5):282-4. |  |  | x |  |  |  |
| Boltri JM, Davis-Smith M, Okosun IS, Seale JP, Foster B. Translation of the National Institutes of Health Diabetes Prevention Program in African American churches. Journal of the National Medical Association. 2011;103(3):194-202. |  |  |  |  | x |  |
| Booth AO, Lowis C, Dean M, Hunter SJ, McKinley MC. Diet and physical activity in the self-management of type 2 diabetes: barriers and facilitators identified by patients and health professionals. Primary health care research & development. 2013;14(3):293-306. |  |  | x |  |  |  |
| Booth AO, Lowis C, Hunter SJ, Dean M, Cardwell CR, McKinley MC. Development and Evaluation of a Computer-Based, Self-Management Tool for People Recently Diagnosed with Type 2 Diabetes. Journal of diabetes research. 2016;2016:3192673. |  |  | x |  |  |  |
| Brathwaite AC, Lemonde M. Health Beliefs and Practices of African Immigrants in Canada. Clinical nursing research. 2016;25(6):626-45. |  |  |  |  | x |  |
| Brathwaite AC, Lemonde M. Exploring Health Beliefs and Practices of Caribbean Immigrants in Ontario to Prevent Type 2 Diabetes. Journal of transcultural nursing: official journal of the Transcultural Nursing Society. 2017;28(1):15-23. |  |  |  |  | x |  |
| Bray P, Cummings DM, Morrissey S, Thompson D, Holbert D, Wilson K, et al. Improved outcomes in diabetes care for rural African Americans. Annals of family medicine. 2013;11(2):145-50. |  |  |  |  | x |  |
| Brinkhues S, Dukers-Muijrers N, Hoebe C, van der Kallen CJH, Dagnelie PC, Koster A, et al. Socially isolated individuals are more prone to have newly diagnosed and prevalent type 2 diabetes mellitus - the Maastricht study. BMC public health. 2017;17. |  |  | x |  |  |  |
| Brokaw SM, Carpenedo D, Campbell P, Butcher MK, Helgerson SD, Harwell TS, et al. Does a History of Gestational Diabetes Mellitus Affect Key Outcomes in Women Participating in a Diabetes Prevention Program? Maternal and child health journal. 2018;22(4):529-37. |  |  | x |  |  |  |
| Brown AF. Patient, system and clinician level interventions to address disparities in diabetes care. Current diabetes reviews. 2007;3(4):244-8. | x |  |  |  |  |  |
| Brown P. Type 2 diabetes risk identification and prevention: NICE public health guidance 38 in practice. Diabetes & Primary Care. 2012;14(5):266-74. | x |  |  |  |  |  |
| Brown SA, García AA, Steinhardt MA, Guevara H, Moore C, Brown A, et al. Culturally Tailored Diabetes Prevention in the Workplace: Focus Group Interviews With Hispanic Employees. Diabetes Educator. 2015;41(2):175-83. |  |  |  |  | x |  |
| Brown SA, Garcia AA, Winter M, Silva L, Brown A, Hanis CL. Integrating education, group support, and case management for diabetic Hispanics. Ethnicity & disease. 2011;21(1):20-6. |  |  |  |  |  |  |
| Brown SA, Hanis CL. Lessons Learned from 20 Years of Diabetes Self-Management Research With Mexican Americans in Starr County, Texas. The Diabetes educator. 2014;40(4):476-87. |  |  |  |  | x |  |
| Brown SD, Ehrlich SF, Kubo A, Tsai AL, Hedderson MM, Quesenberry CP, et al. Lifestyle behaviors and ethnic identity among diverse women at high risk for type 2 diabetes. Social Science & Medicine. 2016;160:87-93. |  |  |  |  | x |  |
| Brown SD, Grijalva CS, Ferrara A. Leveraging EHRs for patient engagement: Perspectives on tailored program outreach. American Journal of Managed Care. 2017;23(7):e223-e30. |  |  | x |  |  |  |
| Brown SD, Partee PN, Feng JR, Quesenberry CP, Hedderson MM, Ehrlich SF, et al. Outreach to diversify clinical trial participation: A randomized recruitment study. Clinical Trials. 2015;12(3):205-11. |  |  |  |  | x |  |
| Brown-Guion SY, Youngerman SM, Hernandez-Tejada MA, Dismuke CE, Egede LE. Racial/ethnic, regional, and rural/urban differences in receipt of diabetes education. The Diabetes educator. 2013;39(3):327-34. |  |  |  |  | x |  |
| Brunk DR, Taylor AG, Williams IC, Cox DJ, Clark ML. A Culturally Appropriate Self-Management Program for Hispanic Adults With Type 2 Diabetes and Low Health Literacy Skills. Journal of Transcultural Nursing. 2017;28(2):187-94. |  |  |  |  | x |  |
| Buchanan LR, Rooks-Peck CR, Finnie RKC, Wethington HR, Jacob V, Fulton JE, et al. Reducing Recreational Sedentary Screen Time A Community Guide Systematic Review. American journal of preventive medicine. 2016;50(3):402-15. |  |  | x |  |  |  |
| Buckley J, Yekta S, Joseph V, Johnson H, Oliverio S, De Groot AS. Vida Sana: a lifestyle intervention for uninsured, predominantly Spanish-speaking immigrants improves metabolic syndrome indicators. Journal of community health. 2015;40(1):116-23. |  |  |  | x |  |  |
| Buis LR, Hirzel L, Turske SA, Des Jardins TR, Yarandi H, Bondurant P. Use of a text message program to raise type 2 diabetes risk awareness and promote health behavior change (part I): assessment of participant reach and adoption. Journal of medical Internet research. 2013;15(12):e281. |  |  | x |  |  |  |
| Bukman AJ, Duijzer G, Haveman-Nies A, Jansen SC, Ter Beek J, Hiddink GJ, et al. Is the success of the SLIMMER diabetes prevention intervention modified by socioeconomic status? A randomised controlled trial. Diabetes research and clinical practice. 2017;129:160-8. |  |  | x |  |  |  |
| Burns D, Soward ACM, Skelly AH, Leeman J, Carlson J. Effective recruitment and retention strategies for older members of rural minorities. Diabetes Educator. 2008;34(6):1045-52. |  |  |  | x |  |  |
| Buysse HE, de Moor GJ, de Maeseneer J. Introducing a telemonitoring platform for diabetic patients in primary care: will it increase the socio-digital divide? Primary care diabetes. 2013;7(2):119-27. |  |  |  | x |  |  |
| Cadzow RB, Vest BM, Craig M, Rowe JS, Kahn LS. “Living Well with Diabetes”: Evaluation of a Pilot Program to Promote Diabetes Prevention and Self-Management in a Medically Underserved Community. Diabetes Spectrum. 2014;27(4):246-55. |  |  | x |  |  |  |
| Calman NS, Hauser D, Schussler L, Crump C. A risk-based intervention approach to eliminate diabetes health disparities. Primary health care research & development. 2018:1-5. |  |  |  |  | x |  |
| Campinha-Bacote J. Culture and diversity issues. A culturally competent model of care for African Americans. Urologic Nursing. 2009;29(1):49-54. |  |  |  | x |  |  |
| Carolan M. Diabetes nurse educators' experiences of providing care for women, with gestational diabetes mellitus, from disadvantaged backgrounds. Journal of clinical nursing. 2014;23(9-10):1374-84. |  | x |  |  |  |  |
| Carolan M, Holman J, Ferrari M. Experiences of diabetes self-management: a focus group study among Australians with type 2 diabetes. Journal of clinical nursing. 2015;24(7-8):1011-23. |  |  | x |  |  |  |
| Carolan M, Steele C, Margetts H. Knowledge of gestational diabetes among a multi-ethnic cohort in Australia. Midwifery. 2010;26(6):579-88. |  |  |  | x |  |  |
| Carolan-Olah M, Cassar A. The Experiences of Older Italian Migrants With Type 2 Diabetes: A Qualitative Study. Journal of Transcultural Nursing. 2018;29(2):172-9. |  |  |  |  | x |  |
| Carolan-Olah MC, Cassar A, Quiazon R, Lynch S. Diabetes care and service access among elderly Vietnamese with type 2 diabetes. BMC health services research. 2013;13:447. |  |  |  |  | x |  |
| Carter EL, Nunlee-Bland G, Callender C. A patient-centric, provider-assisted diabetes telehealth self-management intervention for urban minorities. Perspectives in health information management. 2011;8:1b. |  |  |  |  | x |  |
| Case S, Jernigan V, Gardner A, Ritter P, Heaney CA, Lorig KR. Content and frequency of writing on diabetes bulletin boards: does race make a difference? Journal of medical Internet research. 2009;11(2):e22. |  |  |  |  | x |  |
| Cassimatis M, Kavanagh DJ, Smith AC. Perceived needs for supported self‐management of type 2 diabetes: A qualitative investigation of the potential for a web‐based intervention. Australian Psychologist. 2014;49(2):75-85. |  |  |  |  |  |  |
| Cene CW, Haymore LB, Ellis D, Whitaker S, Henderson S, Lin FC, et al. Implementation of the power to prevent diabetes prevention educational curriculum into rural African American communities: a feasibility study. The Diabetes educator. 2013;39(6):776-85. |  |  |  |  | x |  |
| Cha E, Yang K, Lee J, Min J, Kim KH, Dunbar SB, et al. Understanding cultural issues in the diabetes self-management behaviors of Korean immigrants. The Diabetes educator. 2012;38(6):835-44. |  |  |  |  | x |  |
| Chakkalakal RJ, Camp AW, Magenheimer E, Savoye M, Lubsen J, Lucas G, et al. Preventing diabetes among Fair Haven families: a community-based approach to quality improvement. Journal of health care for the poor and underserved. 2012;23(3 Suppl):247-54. |  |  |  | x |  |  |
| Chandler RF, Monnat SM. Racial/Ethnic Differences in Use of Health Care Services for Diabetes Management. Health Education & Behavior. 2015;42(6):783-92. |  | x |  |  |  |  |
| Chao MT, Handley MA, Quan J, Sarkar U, Ratanawongsa N, Schillinger D. Disclosure of complementary health approaches among low income and racially diverse safety net patients with diabetes. Patient education and counseling. 2015;98(11):1360-6. |  |  |  | x |  |  |
| Chasan-Taber L, Marcus BH, Stanek E, III, Ciccolo JT, Marquez DX, Solomon CG, et al. A randomized controlled trial of prenatal physical activity to prevent gestational diabetes: Design and methods. Journal of Women's Health. 2009;18(6):851-9. |  |  | x |  |  |  |
| Chasan-Taber L, Silveira M, Marcus BH, Braun B, Stanek E, Markenson G. Feasibility and efficacy of a physical activity intervention among pregnant women: the behaviors affecting baby and you (B.A.B.Y.) study. Journal of physical activity & health. 2011;8 Suppl 2:S228-38. |  |  |  |  | x |  |
| Chaudhry R, Tulledge-Scheitel SM, Thomas MR, Hunt VL, Liesinger JT, Rahman AS, et al. Clinical informatics to improve quality of care: a population-based system for patients with diabetes mellitus. Informatics in primary care. 2009;17(2):95-102. |  |  | x |  |  |  |
| Chaufan C, Constantino S, Davis M. ‘It's a full time job being poor’: understanding barriers to diabetes prevention in immigrant communities in the USA. Critical Public Health. 2012;22(2):147-58. |  |  |  |  | x |  |
| Chaufan C, Davis M, Constantino S. The twin epidemics of poverty and diabetes: understanding diabetes disparities in a low-income Latino and immigrant neighborhood. Journal of community health. 2011;36(6):1032-43. |  | x |  |  |  |  |
| Cherrington AL, Agne AA, Lampkin Y, Birl A, Shelton TC, Guzman A, et al. Diabetes Connect: Developing a Mobile Health Intervention to Link Diabetes Community Health Workers With Primary Care. The Journal of ambulatory care management. 2015;38(4):333-45. |  | x |  |  |  |  |
| Cherrington AL, Agne AA, Lampkin Y, Birl A, Shelton TC, Guzman A, et al. Diabetes Connect. Journal of Ambulatory Care Management. 2015;38(4):333-45. |  |  |  |  |  |  |
| Chesla CA, Chun KM, Kwan CML, Mullan JT, Kwong Y, Hsu L, et al. Testing the efficacy of culturally adapted coping skills training for Chinese American immigrants with type 2 diabetes using community‐based participatory research. Research in nursing & health. 2013;36(4):359-72. |  |  |  | x |  |  |
| Chin MH, Goddu AP, Ferguson MJ, Peek ME. Expanding and sustaining integrated health care-community efforts to reduce diabetes disparities. Health promotion practice. 2014;15(2 Suppl):29s-39s. |  |  |  | x |  |  |
| Chlebowy DO, Hood S, LaJoie AS. Facilitators and barriers to self-management of type 2 diabetes among urban African American adults: focus group findings. The Diabetes educator. 2010;36(6):897-905. |  |  |  |  | x |  |
| Choi S, Lee JA, Rush E. ETHNIC AND LANGUAGE DISPARITIES IN DIABETES CARE AMONG CALIFORNIA RESIDENTS. Ethnicity & disease. 2011;21(2):183-9. |  |  |  |  | x |  |
| Choi SE. Diet-specific family support and glucose control among Korean immigrants with type 2 diabetes. The Diabetes educator. 2009;35(6):978-85. |  |  |  | x |  |  |
| Choi SE, Lee JJ, Park JJ, Sarkisian CA. Spousal Support in Diabetes Self-Management Among Korean Immigrant Older Adults. Research in gerontological nursing. 2015;8(2):94-104. |  |  |  |  | x |  |
| Choi SE, Rush E, Henry S. Health literacy in Korean immigrants at risk for type 2 diabetes. Journal of immigrant and minority health. 2013;15(3):553-9. |  |  |  |  | x |  |
| Choi SE, Rush EB. Effect of a short-duration, culturally tailored, community-based diabetes self-management intervention for Korean immigrants: A pilot study. The Diabetes educator. 2012;38(3):377-85. |  |  |  |  | x |  |
| Choi TST, Walker KZ, Palermo C. Diabetes management in a foreign land: A case study on Chinese Australians. Health & social care in the community. 2018;26(2):E225-E32. |  |  |  |  | x |  |
| Choi TST, Walker KZ, Ralston RA, Palermo C. Diabetes education needs of Chinese Australians: A qualitative study. Health Education Journal. 2015;74(2):197-208. |  |  |  |  | x |  |
| Christie D, Channon S. The potential for motivational interviewing to improve outcomes in the management of diabetes and obesity in paediatric and adult populations: a clinical review. Diabetes, obesity & metabolism. 2014;16(5):381-7. |  |  | x |  |  |  |
| Clark L, Vincent D, Zimmer L, Sanchez J. Cultural values and political economic contexts of diabetes among low-income Mexican Americans. Journal of Transcultural Nursing. 2009;20(4):382-94. |  |  |  |  | x |  |
| Claydon A, Campbell-Richards D, Hill M. Living with diabetes: A qualitative review of minority ethnic groups in a deprived London borough. Journal of Diabetes Nursing. 2013;17(3):95-100. |  |  |  |  | x |  |
| Coffman MJ, Ferguson BL, Steinman L, Talbot LA, Dunbar-Jacob J. A health education pilot for Latina women with diabetes. Clinical nursing research. 2013;22(1):70-81. |  |  |  |  | x |  |
| Coffman MJ, Norton CK, Beene L. Diabetes symptoms, health literacy, and health care use in adult Latinos with diabetes risk factors. Journal of cultural diversity. 2012;19(1):4-9. |  |  |  |  | x |  |
| Coleman KJ, Ocana LL, Walker C, Araujo RA, Gutierrez V, Shordon M, et al. Outcomes From a Culturally Tailored Diabetes Prevention Program in Hispanic Families From a Low-Income School Horton Hawks Stay Healthy (HHSH). Diabetes Educator. 2010;36(5):784-92. |  |  | x |  |  |  |
| Cullen KW, Buzek BB. Knowledge about type 2 diabetes risk and prevention of African-American and Hispanic adults and adolescents with family history of type 2 diabetes. The Diabetes educator. 2009;35(5):836-42. |  |  | x |  |  |  |
| Cusi K, Ocampo GL. Unmet needs in Hispanic/Latino patients with type 2 diabetes mellitus. The American journal of medicine. 2011;124(10 Suppl):S2-9. |  |  |  |  | x |  |
| Dauvrin M, Lorant V. Culturally competent interventions in Type 2 diabetes mellitus management: an equity-oriented literature review. Ethnicity & health. 2014;19(6):579-600. |  |  |  | x |  |  |
| Dauvrin M, Lorant V, d’Hoore W, d'Hoore W. Is the Chronic Care Model Integrated Into Research Examining Culturally Competent Interventions for Ethnically Diverse Adults With Type 2 Diabetes Mellitus? A Review. Evaluation & the health professions. 2015;38(4):435-63. |  |  |  | x |  |  |
| Davachi S, Ferrari I. Homelessness and diabetes: reducing disparities in diabetes care through innovations and partnerships. Canadian journal of diabetes. 2012;36(2):75-82. |  |  |  |  | x |  |
| Davies MJ. Diabetes myths and legends: the Iliad and the Odyssey: the 2010 Diabetes UK Arnold Bloom Lecture. Practical Diabetes International. 2011;28(1):37-40. |  |  | x |  |  |  |
| Davis BH, Pope C, Mason PR, Magwood G, Jenkins CM. "It's a wild thing, waiting to get me": stance analysis of African Americans with diabetes. The Diabetes educator. 2011;37(3):409-18. |  |  |  | x |  |  |
| Davis RE, Peterson KE, Rothschild SK, Resnicow K. Pushing the envelope for cultural appropriateness: does evidence support cultural tailoring in type 2 diabetes interventions for Mexican American adults? The Diabetes educator. 2011;37(2):227-38. |  |  |  | x |  |  |
| Davis RM, Hitch AD, Nichols M, Rizvi A, Salaam M, Mayer-Davis EJ. A collaborative approach to the recruitment and retention of minority patients with diabetes in rural community health centers. Contemporary clinical trials. 2009;30(1):63-70. |  |  |  |  | x |  |
| De Kleijn A. Health improvement through dietary management of type 2 diabetes. British journal of community nursing. 2008;13(8):378, 80-3. |  |  | x |  |  |  |
| de Melo LP, de Campos EA. "The group facilitates everything": meanings patients with type 2 diabetes mellitus assigned to health education groups. Revista latino-americana de enfermagem. 2014;22(6):980-7. |  |  | x |  |  |  |
| DeBoer MD. Ethnicity, obesity and the metabolic syndrome: Implications on assessing risk and targeting intervention. Expert Review of Endocrinology and Metabolism. 2011;6(2):279-89. |  |  | x |  |  |  |
| Detz A, Mangione CM, Nunez de Jaimes F, Noguera C, Morales LS, Tseng CH, et al. Language concordance, interpersonal care, and diabetes self-care in rural Latino patients. Journal of general internal medicine. 2014;29(12):1650-6. |  |  |  |  | x |  |
| Dobbins M, Husson H, DeCorby K, LaRocca RL. School-based physical activity programs for promoting physical activity and fitness in children and adolescents aged 6 to 18. The Cochrane database of systematic reviews. 2013(2):Cd007651. |  |  | x |  |  |  |
| Dodani S, Kramer MK, Williams L, Crawford S, Kriska A. Fit body and soul: a church-based behavioral lifestyle program for diabetes prevention in African Americans. Ethnicity & disease. 2009;19(2):135-41. |  |  |  |  | x |  |
| Duggan C, Carosso E, Mariscal N, Islas I, Ibarra G, Holte S, et al. Diabetes prevention in Hispanics: report from a randomized controlled trial. Preventing chronic disease. 2014;11:E28. |  |  |  |  | x |  |
| Early KB, Shultz JA, Corbett C. Assessing Diabetes Dietary Goals and Self-Management Based on In-Depth Interviews With Latino and Caucasian Clients With Type 2 Diabetes. Journal of Transcultural Nursing. 2009;20(4):371-81. |  |  |  |  | x |  |
| Early KB, Shultz JA, Evans M, Corbett CF, Butkus SN, Massey L. Dietary Goal Attainment Measures and Psychosocial Factors among Mexican Americans and Non-Hispanic Whites with Type 2 Diabetes. Ecology of food and nutrition. 2012;51(3):227-46. |  |  |  |  | x |  |
| Eh KX, McGill M, Wong J, Krass I. Cultural issues and other factors that affect self-management of Type 2 Diabetes Mellitus (T2D) by Chinese immigrants in Australia. Diabetes research and clinical practice. 2016;119:97-105. |  |  |  |  | x |  |
| Ekong JI, Russell-Mayhew S, Arthur N. Optimizing diabetes literacy: lessons from African Canadians in Calgary about type 2 diabetes diagnosis. Canadian journal of diabetes. 2013;37(4):231-6. |  |  |  |  | x |  |
| Erickson D. Barriers to physical activity in people with type 2 diabetes enrolled in a worksite diabetes disease management program. The Diabetes educator. 2013;39(5):626-34. |  |  | x |  |  |  |
| Eriksen SJ, Manke B. "Because being fat means being sick": children at risk of type 2 diabetes. Sociological inquiry. 2011;81(4):549-69. |  |  | x |  |  |  |
| Espelt A, Arriola L, Borrell C, Larranaga I, Sandin M, Escolar-Pujolar A. Socioeconomic position and type 2 diabetes mellitus in Europe 1999-2009: a panorama of inequalities. Current diabetes reviews. 2011;7(3):148-58. |  |  |  | x |  |  |
| Faridi Z, Shuval K, Njike VY, Katz JA, Jennings G, Williams M, et al. Partners reducing effects of diabetes (PREDICT): a diabetes prevention physical activity and dietary intervention through African-American churches. Health education research. 2010;25(2):306-15. |  |  |  |  | x |  |
| Faul AC, Yankeelov PA, McCord LR. Inequitable access to health services for older adults with diabetes: potential solutions on a state level. Journal of aging & social policy. 2015;27(1):63-86. |  |  |  |  | x |  |
| Fitzner K, Dietz DA, Moy E. How innovative treatment models and data use are improving diabetes care among older African American adults. Population health management. 2011;14(3):143-55. |  |  |  |  | x |  |
| Friedman MA, Niznik CM, Bolden JR, Yee LM. Reciprocal peer support for post-partum patients with diabetes: A needs assessment for the diabetes buddy program. Journal of Community Health: The Publication for Health Promotion and Disease Prevention. 2016;41(2):354-8. |  |  | x |  |  |  |
| Fritz HA. Learning to do Better: The Transactional Model of Diabetes Self-Management Integration. Qualitative health research. 2015;25(7):875-86. |  |  |  |  | x |  |
| Fritz HA. Challenges to developing diabetes self-management skills in a low-income sample in North Carolina, USA. Health & social care in the community. 2017;25(1):26-34. |  |  |  |  | x |  |
| Gebreab SY, Hickson DA, Sims M, Wyatt SB, Davis SK, Correa A, et al. Neighborhood social and physical environments and type 2 diabetes mellitus in African Americans: The Jackson Heart Study. Health & place. 2017;43:128-37. |  |  |  |  | x |  |
| Gele AA, Torheim LE, Pettersen KS, Kumar B. Beyond Culture and Language: Access to Diabetes Preventive Health Services among Somali Women in Norway. Journal of diabetes research. 2015;2015:549795. |  |  |  |  | x |  |
| Genz J, Haastert B, Muller H, Verheyen F, Cole D, Rathmann W, et al. Socioeconomic factors and effect of evidence-based patient information about primary prevention of type 2 diabetes mellitus--are there interactions? BMC research notes. 2014;7:541. |  |  | x |  |  |  |
| Gilstrap LG, Malhotra R, Peltier-Saxe D, Slicas D, Pineda E, Culhane-Hermann C, et al. Community-based primary prevention programs decrease the rate of metabolic syndrome among socioeconomically disadvantaged women. Journal of women's health (2002). 2013;22(4):322-9. |  |  |  | x |  |  |
| Gimpel N, Marcee A, Kennedy K, Walton J, Lee S, DeHaven MJ. Patient perceptions of a community-based care coordination system. Health promotion practice. 2010;11(2):173-81. |  |  | x |  |  |  |
| Gittelsohn J, Trude A. Diabetes and obesity prevention: changing the food environment in low-income settings. Nutrition reviews. 2017;75(suppl 1):62-9. |  |  |  |  | x |  |
| Glauber H, Karnieli E. Preventing type 2 diabetes mellitus: a call for personalized intervention. The Permanente journal. 2013;17(3):74-9. |  |  | x |  |  |  |
| Goldman ML, Ghorob A, Eyre SL, Bodenheimer T. How do peer coaches improve diabetes care for low-income patients?: a qualitative analysis. The Diabetes educator. 2013;39(6):800-10. |  | x |  |  |  |  |
| Goldschmidt VJ, Colletta B. The challenges of providing diabetes education in resource-limited settings to women with diabetes in pregnancy: Perspectives of an educator. Diabetes Spectrum. 2016;29(2):101-4. |  | x |  |  |  |  |
| Gonzalez Castro F, Shaibi GQ, Boehm-Smith E. Ecodevelopmental contexts for preventing type 2 diabetes in Latino and other racial/ethnic minority populations. Journal of behavioral medicine. 2009;32(1):89-105. |  |  |  |  |  |  |
| Görig T, Schneider S, Bock C, Maul H, Kleinwechter H, Diehl K. Screening for gestational diabetes mellitus in Germany: A qualitative study on pregnant women's attitudes, experiences, and suggestions. Midwifery. 2015;31(11):1026-31. |  |  | x |  |  |  |
| Grace C. Nutrition-related health management in a Bangladeshi community. The Proceedings of the Nutrition Society. 2011;70(1):129-34. |  |  |  | x |  |  |
| Grace C. Symposium 3 (Jointly with the British Dietetic Association): Nutrition management in special populations Nutrition-related health management in a Bangladeshi community. Proceedings of the Nutrition Society. 2011;70(1):129-34. |  |  |  |  |  |  |
| Grace C, Begum R, Subhani S, Kopelman P, Greenhalgh T. Prevention of type 2 diabetes in British Bangladeshis: qualitative study of community, religious, and professional perspectives. BMJ (Clinical research ed). 2008;337:a1931. |  |  |  |  | x |  |
| Grant RW, O'Brien KE, Waxler JL, Vassy JL, Delahanty LM, Bissett LG, et al. Personalized genetic risk counseling to motivate diabetes prevention: a randomized trial. Diabetes care. 2013;36(1):13-9. |  |  | x |  |  |  |
| Greenhalgh T, Clinch M, Afsar N, Choudhury Y, Sudra R, Campbell-Richards D, et al. Socio-cultural influences on the behaviour of South Asian women with diabetes in pregnancy: Qualitative study using a multi-level theoretical approach. BMC medicine. 2015;13(1). |  |  |  |  | x |  |
| Gucciardi E, Chan VWS, Lo BKC, Fortugno M, Horodezny S, Swartzack S. Patients' perspectives on their use of diabetes education centres in Peel-Halton region in Southern Ontario. Canadian journal of diabetes. 2012;36(4):214-7. |  |  | x |  |  |  |
| Gucciardi E, Chan VWS, Manuel L, Sidani S. A systematic literature review of diabetes self-management education features to improve diabetes education in women of Black African/Caribbean and Hispanic/Latin American ethnicity. Patient education and counseling. 2013;92(2):235-45. |  |  |  |  | x |  |
| Guell C. Diabetes management as a Turkish family affair: Chronic illness as a social experience. Annals of Human Biology. 2011;38(4):438-44. |  |  |  |  | x |  |
| Guess ND, Caengprasath N, Dornhorst A, Frost GS. Adherence to NICE guidelines on diabetes prevention in the UK: Effect on patient knowledge and perceived risk. Primary care diabetes. 2015;9(6):407-11. |  |  | x |  |  |  |
| Gumbs JM. Relationship between diabetes self-management education and self-care behaviors among African American women with type 2 diabetes. Journal of cultural diversity. 2012;19(1):18-22. |  |  |  | x |  |  |
| Gupta SS, Teede H, Aroni R. Spicing up your advice for South Asian and Anglo-Australians with type 2 diabetes and CVD: Do cultural constructions of diet matter? Appetite. 2018;120:679-97. |  |  |  | x |  |  |
| Haas LB. Special Considerations for Older Adults With Diabetes Residing in Skilled Nursing Facilities. Diabetes Spectrum. 2014;27(1):37-43. |  |  |  |  | x |  |
| Hahn EA, Burns JL, Jacobs EA, Ganschow PS, Garcia SF, Rutsohn JP, et al. Health Literacy and Patient-Reported Outcomes: A Cross-Sectional Study of Underserved English- and Spanish-Speaking Patients With Type 2 Diabetes. Journal of health communication. 2015;20 Suppl 2:4-15. |  |  |  | x |  |  |
| Hait SSE, Chaar B, McLachlan AJ, Overland J, Basheti IA. Clinical pharmacist-led diabetes management and education program effect on the arabic-speaking people in australia: A pilot study. Jordan Journal of Pharmaceutical Sciences. 2015;8(1). |  |  |  |  | x |  |
| Hall WJ, Zeveloff A, Steckler A, Schneider M, Thompson D, Pham T, et al. Process evaluation results from the HEALTHY physical education intervention. Health education research. 2012;27(2):307-18. |  |  | x |  |  |  |
| Halliday TM, Davy BM, Clark AG, Baugh ME, Hedrick VE, Marinik EL, et al. Dietary intake modification in response to a participation in a resistance training program for sedentary older adults with prediabetes: Findings from the Resist Diabetes study. Eating behaviors. 2014;15(3):379-82. |  |  |  |  | x |  |
| Haltiwanger EP. Experience of mexican-American elders with diabetes: A phenomenological study. Occupational Therapy in Health Care. 2012;26(2-3):150-62. |  |  |  |  | x |  |
| Haltiwanger EP. Effect of a Group Adherence Intervention for Mexican-American Older Adults With Type 2 Diabetes. American Journal of Occupational Therapy. 2012;66(4):447-54. |  |  |  |  | x |  |
| Haltiwanger EP, Brutus H. A culturally sensitive diabetes peer support for older Mexican-Americans. Occupational therapy international. 2012;19(2):67-75. |  |  |  | x |  |  |
| Han BH, Blaum CS, Ferris RE, Min LC, Lee PG. Older adults reporting more diabetes mellitus care have greater 9-year survival. Journal of the American Geriatrics Society. 2015;63(12):2455-62. |  |  |  | x |  |  |
| Hansen E, Landstad B, Svebak S. Motivation for life style changes to improve health in people with impaired glucose tolerance...Proceedings of the 10th Congress of the European Federation for Research in Rehabilitation, Riga, Latvia, 09-12 September 2009. International Journal of Rehabilitation Research. 2009;32:S25-S. |  |  | x |  |  |  |
| Hansen E, Landstad BJ, Hellzén O, Svebak S. Motivation for lifestyle changes to improve health in people with impaired glucose tolerance. Scandinavian journal of caring sciences. 2011;25(3):484-90. |  |  | x |  |  |  |
| Hassaballa I, Ebekozien O, Ogungbadero A, Williams F, Schultz J, Hunter-Skidmore J, et al. Evaluation of a diabetes care coordination program for African-American women living in public housing. Journal of Clinical Outcomes Management. 2015;22(8):365-72. |  |  |  |  | x |  |
| Hasseler MK, Von Der Heide M, Indefrey S. Resources for and barriers to effective diabetes care management- experiences and perspectives of people with type 2 diabetes. Journal of Public Health. 2011;19(1):65-71. |  |  | x |  |  |  |
| Hawkins J, Watkins DC, Kieffer E, Spencer M, Espitia N, Anderson M. Psychosocial factors that influence health care use and self-management for African American and Latino men with type 2 diabetes: An exploratory study. The Journal of Men's Studies. 2015;23(2):161-76. | x |  |  |  |  |  |
| Hawkins M, Hosker M, Marcus BH, Rosal MC, Braun B, Stanek EJ, 3rd, et al. A pregnancy lifestyle intervention to prevent gestational diabetes risk factors in overweight Hispanic women: a feasibility randomized controlled trial. Diabetic medicine : a journal of the British Diabetic Association. 2015;32(1):108-15. |  |  |  |  | x |  |
| Hawthorne K, Robles Y, Cannings-John R, Edwards AG. Culturally appropriate health education for type 2 diabetes mellitus in ethnic minority groups. The Cochrane database of systematic reviews. 2008(3):Cd006424. |  |  |  |  | x |  |
| Hawthorne K, Robles Y, Cannings-John R, Edwards AG. Culturally appropriate health education for Type 2 diabetes in ethnic minority groups: a systematic and narrative review of randomized controlled trials. Diabetic medicine : a journal of the British Diabetic Association. 2010;27(6):613-23. |  |  |  |  | x |  |
| Hays LM, Finch EA, Saha C, Marrero DG, Ackermann RT. Effect of Self-Efficacy on Weight Loss: A Psychosocial Analysis of a Community-Based Adaptation of the Diabetes Prevention Program Lifestyle Intervention. Diabetes Spectrum. 2014;27(4):270-5. |  |  | x |  |  |  |
| Hays LM, Hoen HM, Slaven JE, Finch EA, Marrero DG, Saha C, et al. Effects of a Community-based Lifestyle Intervention on Change in Physical Activity Among Economically Disadvantaged Adults With Prediabetes. American Journal of Health Education. 2016;47(5):266-78. |  |  |  |  | x |  |
| Head BJ, Barr KL, Baker SK. Mexican American Parents' Perceptions of Childhood Risk Factors for Type 2 Diabetes. Journal of School Nursing. 2011;27(1):51-60. |  |  | x |  |  |  |
| Heideman WH, de Wit M, Middelkoop BJ, Nierkens V, Stronks K, Verhoeff AP, et al. Diabetes risk reduction in overweight first degree relatives of type 2 diabetes patients: effects of a low-intensive lifestyle education program (DiAlert) A randomized controlled trial. Patient education and counseling. 2015;98(4):476-83. |  |  | x |  |  |  |
| Heisler M, Choi H, Palmisano G, Mase R, Richardson C, Fagerlin A, et al. Comparison of community health worker-led diabetes medication decision-making support for low-income Latino and African American adults with diabetes using e-health tools versus print materials: a randomized, controlled trial. Annals of internal medicine. 2014;161(10 Suppl):S13-22. |  |  |  | x |  |  |
| Heitkemper EM, Mamykina L, Travers J, Smaldone A. Do health information technology self-management interventions improve glycemic control in medically underserved adults with diabetes? A systematic review and meta-analysis. Journal of the American Medical Informatics Association : JAMIA. 2017;24(5):1024-35. |  |  | x |  |  |  |
| Hellyer NE, Haddock-Fraser J. Reporting diet-related health issues through newspapers: portrayal of cardiovascular disease and Type 2 diabetes. Health education research. 2011;26(1):13-25. |  |  | x |  |  |  |
| Helmink JHM, Kremers SPJ, van Brussel-Visser FN, de Vries NK. Sitting Time and Body Mass Index in Diabetics and Pre-Diabetics Willing to Participate in a Lifestyle Intervention. International journal of environmental research and public health. 2011;8(9):3747-58. |  |  | x |  |  |  |
| Hempler NF, Nicic S, Ewers B, Willaing I. Dietary education must fit into everyday life: A qualitative study of people with a pakistani background and type 2 diabetes. Patient preference and adherence. 2015;9:347-54. |  |  |  |  | x |  |
| Herber OR, Gies V, Schwappach D, Thurmann P, Wilm S. Patient information leaflets: informing or frightening? A focus group study exploring patients' emotional reactions and subsequent behavior towards package leaflets of commonly prescribed medications in family practices. BMC family practice. 2014;15:163. |  |  |  |  |  |  |
| Hernandez AE, Marcus MD, Hirst K, Faith MS, Goldberg L, Trevino RP. Impact of implementation and conduct of the HEALTHY primary prevention trial on student performance. American journal of health promotion : AJHP. 2014;29(1):55-8. |  | x |  |  |  |  |
| Heslehurst N. Identifying 'at risk' women and the impact of maternal obesity on National Health Service maternity services. The Proceedings of the Nutrition Society. 2011;70(4):439-49. |  |  | x |  |  |  |
| Heslehurst N. Symposium I: Consequences of obesity and overweight during pregnancy Identifying 'at risk' women and the impact of maternal obesity on National Health Service maternity services. Proceedings of the Nutrition Society. 2011;70(4):439-49. | x |  |  |  |  |  |
| Hesselink AE, Rutten GE, Slootmaker SM, de Weerdt I, Raaijmakers LG, Jonkers R, et al. Effects of a lifestyle program in subjects with Impaired Fasting Glucose, a pragmatic cluster-randomized controlled trial. BMC family practice. 2015;16:183. |  |  | x |  |  |  |
| Heuman AN, Scholl JC, Wilkinson K. Rural Hispanic populations at risk in developing diabetes: sociocultural and familial challenges in promoting a healthy diet. Health communication. 2013;28(3):260-74. |  |  |  |  | x |  |
| Higginbottom GMA, Vallianatos H, Forgeron J, Gibbons D, Mamede F, Barolia R. Food choices and practices during pregnancy of immigrant women with high-risk pregnancies in Canada: a pilot study. BMC pregnancy and childbirth. 2014;14. |  |  |  |  | x |  |
| Hilger J, Loerbroks A, Diehi K. Eating behaviour of university students in Germany: Dietary intake, barriers to healthy eating and changes in eating behaviour since the time of matriculation. Appetite. 2017;109:100-7. |  |  | x |  |  |  |
| Hill J. Preventing type 2 diabetes: a role for every practitioner. Community practitioner: the journal of the Community Practitioners' & Health Visitors' Association. 2012;85(10):34-6. | x |  |  |  |  |  |
| Hill J, Nielsen M, Fox MH. Understanding the social factors that contribute to diabetes: a means to informing health care and social policies for the chronically ill. The Permanente journal. 2013;17(2):67-72. |  |  | x |  |  |  |
| Hill-Briggs F, Lazo M, Peyrot M, Doswell A, Chang YT, Hill MN, et al. Effect of problem-solving-based diabetes self-management training on diabetes control in a low income patient sample. Journal of general internal medicine. 2011;26(9):972-8. |  |  |  |  | x |  |
| Hill-Briggs F, Renosky R, Lazo M, Bone L, Hill M, Levine D, et al. Development and pilot evaluation of literacy-adapted diabetes and CVD education in urban, diabetic African Americans. Journal of general internal medicine. 2008;23(9):1491-4. |  |  |  |  | x |  |
| Hinman SK, Smith KB, Quillen DM, Smith MS. Exercise in Pregnancy: A Clinical Review. Sports health. 2015;7(6):527-31. |  |  | x |  |  |  |
| Hjelm K, Bard K, Apelqvist J. Gestational diabetes: prospective interview-study of the developing beliefs about health, illness and health care in migrant women. Journal of clinical nursing. 2012;21(21/22):3244-56. |  |  |  |  | x |  |
| Hjelm K, Bard K, Apelqvist J. A qualitative study of developing beliefs about health, illness and healthcare in migrant African women with gestational diabetes living in Sweden. BMC Women's Health. 2018;18(1). |  |  |  |  | x |  |
| Hjelm K, Berntorp K, Apelqvist J. Beliefs about health and illness in Swedish and African‐born women with gestational diabetes living in Sweden. Journal of clinical nursing. 2012;21(9-10):1374-86. |  |  |  |  | x |  |
| Ho EY, Chesla CA, Chun KM. Health communication with Chinese Americans about type 2 diabetes. The Diabetes educator. 2012;38(1):67-76. |  |  |  |  | x |  |
| Ho EY, Tran H, Chesla CA. Assessing the cultural in culturally sensitive printed patient-education materials for Chinese Americans with type 2 diabetes. Health communication. 2015;30(1):39-49. |  |  |  |  | x |  |
| Hooks-Anderson DR, Crannage EF, Salas J, Scherrer JF. Race and Referral to Diabetes Education in Primary Care Patients With Prediabetes and Diabetes. Diabetes Educator. 2015;41(3):281-9. |  |  | x |  |  |  |
| Horvat L, Horey D, Romios P, Kis-Rigo J. Cultural competence education for health professionals. Cochrane Database of Systematic Reviews. 2014(5). |  | x |  |  |  |  |
| Hosler AS, Solanki MN, Savadatti S. Assessing Needs and Feasibility of Diabetes Self-management Coaching at Faith-Based Organizations for Indo-Guyanese Immigrants. The Diabetes educator. 2015;41(3):320-7. |  |  |  |  | x |  |
| Hu J, Amirehsani K, Wallace DC, Letvak S. Perceptions of Barriers in Managing Diabetes Perspectives of Hispanic Immigrant Patients and Family Members. Diabetes Educator. 2013;39(4):494-503. |  |  |  |  | x |  |
| Hu J, Amirehsani KA, Wallace DC, McCoy TP, Silva Z. A Family-Based, Culturally Tailored Diabetes Intervention for Hispanics and Their Family Members. The Diabetes educator. 2016;42(3):299-314. |  |  |  |  | x |  |
| Hu J, Wallace DC, Amirehsani KA, McCoy TP, Coley SL, Wiseman KD, et al. Improving Physical Activity in Hispanics with Diabetes and their Families. Public health nursing (Boston, Mass). 2015;32(6):625-33. |  |  |  |  | x |  |
| Hu J, Wallace DC, McCoy TP, Amirehsani KA. A family-based diabetes intervention for Hispanic adults and their family members. The Diabetes educator. 2014;40(1):48-59. |  |  |  |  | x |  |
| Hu SH, Fu MR, Liu S, Lin YK, Chang WY. Physical Activity Among Chinese American Immigrants with Prediabetes or Type 2 Diabetes. American Journal of Nursing. 2018;118(2):24-32. |  |  |  |  | x |  |
| Huckfeldt PJ, Meeker D, Peters A, Guterman JJ, Diaz G, Jr., Goldman DP. Diabetes management for low-income patients in Los Angeles: two strategies improved disease control in the short term. Health affairs (Project Hope). 2012;31(1):168-76. |  |  |  |  | x |  |
| Hughes MM, Yang E, Ramanathan D, Benjamins MR. Community-based diabetes community health worker intervention in an underserved Chicago population. Journal of Community Health: The Publication for Health Promotion and Disease Prevention. 2016;41(6):1249-56. |  |  | x |  |  |  |
| Hunt C, Grant J, Appel S. An Integrative Review of Community Health Advisors in Type 2 Diabetes. Journal of community health. 2011;36(5):883-93. |  |  |  |  |  |  |
| Hyman I, Gucciardi E, Patychuk D, Rummens JA, Shakya Y, Kljujic D, et al. Self-management, health service use and information seeking for diabetes care among Black Caribbean immigrants in Toronto. Canadian journal of diabetes. 2014;38(1):32-7. |  |  |  |  | x |  |
| Hyman I, Patychuk D, Zaidi Q, Kljujic D, Shakya YB, Rummens JA, et al. Self-management, health service use and information seeking for diabetes care among recent immigrants in Toronto. Chronic diseases and injuries in Canada. 2012;33(1):12-8. |  |  |  |  | x |  |
| Hyman I, Shakya Y, Jembere N, Gucciardi E, Vissandjee B. Provider- and patient-related determinants of diabetes self-management among recent immigrants: Implications for systemic change. Canadian family physician Medecin de famille canadien. 2017;63(2):e137-e44. |  |  |  |  | x |  |
| Iljaz R, Brodnik A, Zrimec T, Cukjati I. E-HEALTHCARE FOR DIABETES MELLITUS TYPE 2 PATIENTS - A RANDOMISED CONTROLLED TRIAL IN SLOVENIA. Zdravstveno Varstvo. 2017;56(3):150-7. |  |  | x |  |  |  |
| Infanti JJ, O'Dea A, Gibson I, McGuire BE, Newell J, Glynn LG, et al. Reasons for participation and non-participation in a diabetes prevention trial among women with prior gestational diabetes mellitus (GDM). BMC medical research methodology. 2014;14. |  |  | x |  |  |  |
| Inouye J, Matsuura C, Li D, Castro R, Leake A. Lifestyle intervention for Filipino Americans at risk for diabetes. Journal of community health nursing. 2014;31(4):225-37. |  |  |  |  | x |  |
| Isaacs T, Hunt D, Ward D, Rooshenas L, Edwards L. The Inclusion of Ethnic Minority Patients and the Role of Language in Telehealth Trials for Type 2 Diabetes: A Systematic Review. Journal of medical Internet research. 2016;18(9):e256. |  |  | x |  |  |  |
| Ishikawa H, Takeuchi T, Yano E. Measuring functional, communicative, and critical health literacy among diabetic patients. Diabetes care. 2008;31(5):874-9. |  |  | x |  |  |  |
| Ishikawa H, Yano E. The relationship of patient participation and diabetes outcomes for patients with high vs. low health literacy. Patient education and counseling. 2011;84(3):393-7. |  |  | x |  |  |  |
| Ishikawa H, Yano E, Fujimori S, Kinoshita M, Yamanouchi T, Yoshikawa M, et al. Patient health literacy and patient–physician information exchange during a visit. Family practice. 2009;26(6):517-23. |  |  | x |  |  |  |
| Islam N, Riley L, Wyatt L, Tandon SD, Tanner M, Mukherji-Ratnam R, et al. Protocol for the DREAM Project (Diabetes Research, Education, and Action for Minorities): a randomized trial of a community health worker intervention to improve diabetic management and control among Bangladeshi adults in NYC. BMC public health. 2014;14. |  |  |  |  | x |  |
| Islam NS, Wyatt LC, Patel SD, Shapiro E, Tandon SD, Mukherji BR, et al. Evaluation of a community health worker pilot intervention to improve diabetes management in Bangladeshi immigrants with type 2 diabetes in New York City. The Diabetes educator. 2013;39(4):478-93. |  |  |  |  | x |  |
| Islam NS, Zanowiak JM, Wyatt LC, Chun K, Lee L, Kwon SC, et al. A randomized-controlled, pilot intervention on diabetes prevention and healthy lifestyles in the New York City Korean community. Journal of community health. 2013;38(6):1030-41. |  |  |  |  | x |  |
| Islam NS, Zanowiak JM, Wyatt LC, Kavathe R, Singh H, Kwon SC, et al. Diabetes prevention in the New York City Sikh Asian Indian community: a pilot study. International journal of environmental research and public health. 2014;11(5):5462-86. |  |  |  |  | x |  |
| Ivanov LL, Wallace DC, Hernandez C, Hyde Y. Diabetes risks and health literacy in southern African American and Latino women. Journal of community health nursing. 2015;32(1):12-23. |  |  |  |  | x |  |
| Ivey SL, Tseng W, Kurtovich E, Lui B, Weir RC, Liu J, et al. Evaluating a culturally and linguistically competent health coach intervention for Chinese-American patients with diabetes. Diabetes Spectrum. 2012;25(2):93-102. |  |  |  |  | x |  |
| James-Todd T, Janevic T, Brown FM, Savitz DA. Race/Ethnicity, Educational Attainment, and Pregnancy Complications in New York City Women with Pre-existing Diabetes. Paediatric and perinatal epidemiology. 2014;28(2):157-65. |  |  | x |  |  |  |
| Jansà M, Diaz M, Franch J, Vidal M, Gomis R. Anthropologic study of immigrant patients with T2DM from Morocco to Spain: practical implications for therapeutic patient education. European Diabetes Nursing. 2010;7(1):24-8. |  |  |  |  | x |  |
| Jansink R, Braspenning J, van der Weijden T, Niessen L, Elwyn G, Grol R. Nurse-led motivational interviewing to change the lifestyle of patients with type 2 diabetes (MILD-project): protocol for a cluster, randomized, controlled trial on implementing lifestyle recommendations. BMC health services research. 2009;9:19. |  |  | x |  |  |  |
| Janus ED, Best JD, Davis-Lameloise N, Philpot B, Hernan A, Bennett CM, et al. Scaling-up from an implementation trial to state-wide coverage: results from the preliminary Melbourne Diabetes Prevention Study. Trials. 2012;13:152. |  |  | x |  |  |  |
| Jefferson V, Jaser SS, Lindemann E, Galasso P, Beale A, Holl MG, et al. Coping Skills Training in a Telephone Health Coaching Program for Youth at Risk for Type 2 Diabetes. Journal of Pediatric Health Care. 2011;25(3):153-61. |  |  | x |  |  |  |
| Jirojwong S, Brownhill S, Dahlen HG, Johnson M, Schmied V. Going up, going down: the experience, control and management of gestational diabetes mellitus among Southeast Asian migrant women living in urban Australia. Health promotion journal of Australia: official journal of Australian Association of Health Promotion Professionals. 2017;28(2):123-31. |  |  |  |  | x |  |
| Jiwrajka M, Mahmoud A, Uppal M. A Rohingya refugee's journey in Australia and the barriers to accessing healthcare. BMJ case reports. 2017;2017. |  |  |  |  | x |  |
| Johansen KS, Bjørge B, Hjellset VT, Holmboe-Ottesen G, Råberg M, Wandel M, et al. Changes in food habits and motivation for healthy eating among Pakistani women living in Norway: results from the InnvaDiab-DEPLAN study. Public health nutrition. 2010;13(6):858-67. |  |  |  |  | x |  |
| Johnson M, Everson-Hock E, Jones R, Woods HB, Payne N, Goyder E. What are the barriers to primary prevention of type 2 diabetes in black and minority ethnic groups in the UK? A qualitative evidence synthesis. Diabetes research and clinical practice. 2011;93(2):150-8. |  |  | x |  |  |  |
| Johnson P, Hartig MT, Frazier R, Clayton M, Oliver G, Nelson BW, et al. Engaging faith-based resources to initiate and support diabetes self-management among African Americans: A collaboration of informal and formal systems of care. Health promotion practice. 2014;15(2, Suppl):71S-82S. |  |  |  |  | x |  |
| Joiner KL. Risk perception of developing diabetes among Spanish-speaking foreign-born Latinos: University of California, San Francisco; 2015. | x |  |  |  |  |  |
| Joiner KL, Sternberg RM, Kennedy CM, Fukuoka Y, Chen J-L, Janson SL. Perception of Risk for Developing Diabetes Among Foreign-Born Spanish-Speaking US Latinos. Diabetes Educator. 2016;42(4):418-28. |  |  |  |  | x |  |
| Jones RA, Utz SW, Williams IC, Hinton I, Alexander G, Moore C, et al. Family interactions among African Americans diagnosed with type 2 diabetes. The Diabetes educator. 2008;34(2):318-26. |  |  |  |  | x |  |
| Jones V, Crowe M. How people from ethnic minorities describe their experiences of managing type-2 diabetes mellitus: A qualitative meta-synthesis. International journal of nursing studies. 2017;76:78-91. |  |  |  |  | x |  |
| Joo JY, Lee H. Barriers to and facilitators of diabetes self-management with elderly Korean-American immigrants. International nursing review. 2016;63(2):277-84. | x |  |  |  |  |  |
| Kamimura A, Christensen N, Nourian MM, Myers K, Saunders A, Solis SP, et al. The Relationship Between Diabetes Attitudes and Treatment Among Free Clinic Patients and Volunteers. Journal of community health. 2014;39(6):1186-92. |  |  | x |  |  |  |
| Kandula NR, Nsiah-Kumi PA, Makoul G, Sager J, Zei CP, Glass S, et al. The relationship between health literacy and knowledge improvement after a multimedia type 2 diabetes education program. Patient education and counseling. 2009;75(3):321-7. |  |  | x |  |  |  |
| Kane EP, Collinsworth AW, Schmidt KL, Brown RM, Snead CA, Barnes SA, et al. Improving diabetes care and outcomes with community health workers. Family practice. 2016;33(5):523-8. |  |  |  |  | x |  |
| Kaptein S, Evans M, McTavish S, Banerjee AT, Feig DS, Lowe J, et al. The subjective impact of a diagnosis of gestational diabetes among ethnically diverse pregnant women: a qualitative study. Canadian journal of diabetes. 2015;39(2):117-22. |  |  | x |  |  |  |
| Kapustin JF. Postpartum management for gestational diabetes mellitus: policy and practice implications. Journal of the American Academy of Nurse Practitioners. 2008;20(11):547-54. |  |  | x |  |  |  |
| Katula JA, Vitolins MZ, Morgan TM, Lawlor MS, Blackwell CS, Isom SP, et al. The Healthy Living Partnerships to Prevent Diabetes study: 2-year outcomes of a randomized controlled trial. American journal of preventive medicine. 2013;44(4 Suppl 4):S324-32. |  |  | x |  |  |  |
| Katula JA, Vitolins MZ, Rosenberger EL, Blackwell C, Espeland MA, Lawlor MS, et al. Healthy Living Partnerships to Prevent Diabetes (HELP PD): Design and methods. Contemporary clinical trials. 2010;31(1):71-81. |  |  | x |  |  |  |
| Katula JA, Vitolins MZ, Rosenberger EL, Blackwell CS, Morgan TM, Lawlor MS, et al. One-year results of a community-based translation of the Diabetes Prevention Program: Healthy-Living Partnerships to Prevent Diabetes (HELP PD) Project. Diabetes care. 2011;34(7):1451-7. |  |  | x |  |  |  |
| Kaufman N. Using health information technology to prevent and treat diabetes. International journal of clinical practice. 2012;66(SUPPL. 175):40-8. |  |  | x |  |  |  |
| Kaufman N, Dadashi M. Using Digital Health Technology to Prevent and Treat Diabetes. Diabetes Technology and Therapeutics. 2018;20(S1):S71-S85. |  |  |  |  | x |  |
| Kauhl B, Pieper J, Schweikart J, Keste A, Moskwyn M. [Spatial Distribution of Type 2 Diabetes Mellitus in Berlin: Application of a Geographically Weighted Regression Analysis to Identify Location-Specific Risk Groups]. Gesundheitswesen (Bundesverband der Arzte des Offentlichen Gesundheitsdienstes (Germany)). 2018;80(S 02):S64-s70. |  |  |  |  | x |  |
| Kauhl B, Schweikart J, Krafft T, Keste A, Moskwyn M. Do the risk factors for type 2 diabetes mellitus vary by location? A spatial analysis of health insurance claims in Northeastern Germany using kernel density estimation and geographically weighted regression. International journal of health geographics. 2016;15(1):38. |  |  | x |  |  |  |
| Keely E, Clark H, Karovitch A, Graham I. Screening for type 2 diabetes following gestational diabetes: family physician and patient perspectives. Canadian family physician Medecin de famille canadien. 2010;56(6):558-63. |  |  | x |  |  |  |
| Keene DE, Guo M, Murillo S. “That wasn't really a place to worry about diabetes”: Housing access and diabetes self-management among low-income adults. Social Science and Medicine. 2018;197:71-7. |  |  |  |  | x |  |
| Kek T, Kuželički NK, Raščan IM, Geršak K. Characteristics of health behaviours and health status indicators among pregnant women in Slovenia. Zdravniski Vestnik. 2017;86(7-8):295-317. |  |  | x |  |  |  |
| Khan MA, Shah S, Grudzien A, Onyejekwe N, Banskota P, Karim S, et al. A diabetes education multimedia program in the waiting room setting. Diabetes Therapy. 2011;2(3):178-88. |  |  |  |  | x |  |
| Khanam S, Costarelli V. Attitudes towards health and exercise of overweight women. Journal of the Royal Society for the Promotion of Health. 2008;128(1):26-30. |  |  |  |  | x |  |
| Khavandi K, Amer H, Ibrahim B, Brownrigg J. Strategies for preventing type 2 diabetes: An update for clinicians. Therapeutic Advances in Chronic Disease. 2013;4(5):242-61. |  |  | x |  |  |  |
| Khunti K, Camosso-Stefinovic J, Carey M, Davies MJ, Stone MA. Educational interventions for migrant South Asians with Type 2 diabetes: a systematic review. Diabetic medicine : a journal of the British Diabetic Association. 2008;25(8):985-92. |  |  |  |  | x |  |
| Khunti K, Hanif W, Karet B, Mughal S, Patel K. A practical approach to managing type 2 diabetes in the UK south Asian population. Diabetes & Primary Care. 2011;13(1):47-55. |  |  |  |  | x |  |
| Khunti K, Stone MA, Bankart J, Sinfield P, Pancholi A, Walker S, et al. Primary prevention of type-2 diabetes and heart disease: action research in secondary schools serving an ethnically diverse UK population. Journal of Public Health. 2008;30(1):30-7. |  |  | x |  |  |  |
| Kieffer EC, Welmerink DB, Sinco BR, Welch KB, Rees Clayton EM, Schumann CY, et al. Dietary outcomes in a Spanish-language randomized controlled diabetes prevention trial with pregnant Latinas. American journal of public health. 2014;104(3):526-33. |  |  |  |  | x |  |
| Kim KK, Logan HC, Young E, Sabee CM. Youth-centered design and usage results of the iN Touch mobile self-management program for overweight/obesity. Personal and Ubiquitous Computing. 2015;19(1):59-68. |  |  | x |  |  |  |
| Kim MT, Han HR, Song HJ, Lee JE, Kim J, Ryu JP, et al. A community-based, culturally tailored behavioral intervention for Korean Americans with type 2 diabetes. The Diabetes educator. 2009;35(6):986-94. |  |  |  |  | x |  |
| Kim MT, Kim KB, Huh B, Nguyen T, Han H-R, Bone LR, et al. The effect of a community-based self-help intervention: Korean Americans with type 2 diabetes. American journal of preventive medicine. 2015;49(5):726-37. |  |  |  |  | x |  |
| Kindarara DM, McEwen MM, Crist JD, Loescher LJ. Health-Illness Transition Experiences With Type 2 Diabetes Self-management of Sub-Saharan African Immigrants in the United States. Diabetes Educator. 2017;43(5):506-18. |  |  |  |  | x |  |
| Kline KN, Montealegre JR, Rustveld LO, Glover TL, Chauca G, Reed BC, et al. Incorporating Cultural Sensitivity into Interactive Entertainment-Education for Diabetes Self-Management Designed for Hispanic Audiences. Journal of health communication. 2016;21(6):658-68. |  |  |  |  | x |  |
| Kofahl C, von dem Knesebeck O, Hollmann J, Mnich E. [Diabetes-specific health literacy: what do Turkish immigrants with diabetes mellitus 2 know about their disease?]. Gesundheitswesen (Bundesverband der Arzte des Offentlichen Gesundheitsdienstes (Germany)). 2013;75(12):803-11. |  |  |  |  | x |  |
| Kohinor MJE, Stronks K, Haafkens JA. Factors affecting the disclosure of diabetes by ethnic minority patients: a qualitative study among Surinamese in the Netherlands. BMC public health. 2011;11. |  |  |  |  | x |  |
| Krok-Schoen JL, Shim R, Nagel R, Lehman J, Myers M, Lucey C, et al. Outcomes of a health coaching intervention delivered by medical students for older adults with uncontrolled type 2 diabetes. Gerontology & geriatrics education. 2017;38(3):257-70. |  |  |  |  | x |  |
| Lachance L, Kelly RP, Wilkin M, Burke J, Waddell S. Community-Based Efforts to Prevent and Manage Diabetes in Women Living in Vulnerable Communities. Journal of community health. 2018;43(3):508-17. |  |  |  |  | x |  |
| Le H, Wong S, Iftikar T, Keenan H, King GL, Hsu WC. Characterization of Factors Affecting Attainment of Glycemic Control in Asian Americans With Diabetes in a Culturally Specific Program. Diabetes Educator. 2013;39(4):452-61. |  |  |  | x |  |  |
| Leake A, Bermudo V, Jacob J, Jacob M, Inouye J. Health is Wealth: Methods to Improve Attendance in a Lifestyle Intervention for a Largely Immigrant Filipino-American Sample. Journal of Immigrant & Minority Health. 2012;14(3):475-80. |  |  |  | x |  |  |
| Ledoux T, Van Den Berg P, Leung P, Berens PD. Factors associated with knowledge of personal gestational weight gain recommendations. BMC research notes. 2015;8:349. |  |  |  | x |  |  |
| Lee LT, Willig AL, Agne AA, Locher JL, Cherrington AL. Challenges to Healthy Eating Practices: A Qualitative Study of Non-Hispanic Black Men Living With Diabetes. The Diabetes educator. 2016;42(3):325-35. |  |  |  |  | x |  |
| Lee S, Shin S. Effectiveness of virtual reality using video gaming technology in elderly adults with diabetes mellitus. Diabetes technology & therapeutics. 2013;15(6):489-96. |  |  |  |  | x |  |
| Lehrer HM, Dubois SK, Brown SA, Steinhardt MA. Resilience-based Diabetes Self-management Education: Perspectives From African American Participants, Community Leaders, and Healthcare Providers. The Diabetes educator. 2017;43(4):367-77. |  |  |  |  | x |  |
| Leppanen M, Aittasalo M, Raitanen J, Kinnunen TI, Kujala UM, Luoto R. Physical activity during pregnancy: predictors of change, perceived support and barriers among women at increased risk of gestational diabetes. Maternal and child health journal. 2014;18(9):2158-66. |  |  | x |  |  |  |
| Leung AY, Bo A, Hsiao HY, Wang SS, Chi I. Health literacy issues in the care of Chinese American immigrants with diabetes: a qualitative study. BMJ open. 2014;4(11):e005294. |  |  |  | x |  |  |
| Levin-Zamir D, Badarne S, Najami M, Noy SG, Poraz I, Shapira M, et al. The use of focus groups as a basis for planning and implementing culturally appropriate health promotion among people with diabetes in the Arab community. Global health promotion. 2016;23(1):5-14. |  |  | x |  |  |  |
| Lewis MA, Williams PA, Fitzgerald TM, Heminger CL, Hobbs CL, Moultrie RR, et al. Improving the implementation of diabetes self-management: Findings from the Alliance to Reduce Disparities in Diabetes. Health promotion practice. 2014;15(2, Suppl):83S-91S. |  |  |  |  | x |  |
| Lin J, Marcum CS, Wilkinson AV, Koehly LM. Developing Shared Appraisals of Diabetes Risk Through Family Health History Feedback: The Case of Mexican-Heritage Families. Annals of behavioral medicine: a publication of the Society of Behavioral Medicine. 2018;52(3):262-71. | x |  |  |  |  |  |
| Lindberg SM, DeBoth A, Anderson CK. Effect of a best practice alert on gestational weight gain, health services, and pregnancy outcomes. Maternal and child health journal. 2016;20(10):2169-78. |  |  | x |  |  |  |
| Lipman TH, Schucker MM, Ratcliffe SJ, Holmberg T, Baier S, Deatrick JA. Diabetes risk factors in children: a partnership between nurse practitioner and high school students. MCN The American journal of maternal child nursing. 2011;36(1):56-62. |  |  | x |  |  |  |
| Lipscombe L. In high-risk pregnant women, an individualized lifestyle intervention reduced gestational diabetes mellitus. ACP Journal Club. 2015;163(12):1-. |  |  | x |  |  |  |
| Lipscombe LL, Banerjee AT, McTavish S, Mukerji G, Lowe J, Ray J, et al. Readiness for diabetes prevention and barriers to lifestyle change in women with a history of gestational diabetes mellitus: Rationale and study design. Diabetes research and clinical practice. 2014;106(1):57-66. |  |  | x |  |  |  |
| Lirussi F. The global challenge of type 2 diabetes and the strategies for response in ethnic minority groups. Diabetes/metabolism research and reviews. 2010;26(6):421-32. |  |  |  | x |  |  |
| Livaudais JC, Thompson B, Islas I, Ibarra G, Godina R, Coronado GD. Type 2 diabetes among rural Hispanics in Washington State: perspectives from community stakeholders. Health promotion practice. 2010;11(4):589-99. |  |  | x |  |  |  |
| Lloyd CE, Johnson MR, Mughal S, Sturt JA, Collins GS, Roy T, et al. Securing recruitment and obtaining informed consent in minority ethnic groups in the UK. BMC health services research. 2008;8:68. |  |  |  | x |  |  |
| Long AF, Gambling T. Enhancing health literacy and behavioural change within a tele-care education and support intervention for people with type 2 diabetes. Health expectations : an international journal of public participation in health care and health policy. 2012;15(3):267-82. |  |  |  |  | x |  |
| Long JA, Jahnle EC, Richardson DM, Loewenstein G, Volpp KG. Peer Mentoring and Financial Incentives to Improve Glucose Control in African American Veterans A Randomized Trial. Annals of internal medicine. 2012;156(6):416-U60. |  |  |  |  | x |  |
| Lopez L, Tan-McGrory A, Horner G, Betancourt JR. Eliminating disparities among Latinos with type 2 diabetes: Effective eHealth strategies. Journal of diabetes and its complications. 2016;30(3):554-60. |  |  |  |  | x |  |
| Lucas A, Murray E, Kinra S. Heath beliefs of UK South Asians related to lifestyle diseases: a review of qualitative literature. Journal of obesity. 2013;2013:827674. |  |  |  |  | x |  |
| Lucas C, Charlton KE, Yeatman H. Nutrition advice during pregnancy: Do women receive it and can health professionals provide it? Maternal and child health journal. 2014;18(10):2465-78. |  |  | x |  |  |  |
| Ludwig AF, Cox P, Ellahi B. Social and cultural construction of obesity among Pakistani Muslim women in North West England. Public health nutrition. 2011;14(10):1842-50. |  |  |  |  | x |  |
| Luoto RM, Kinnunen TI, Aittasalo M, Ojala K, Mansikkamaki K, Toropainen E, et al. Prevention of Gestational Diabetes: Design of a Cluster-Randomized Controlled Trial and One-Year Follow-Up. BMC pregnancy and childbirth. 2010;10. |  |  | x |  |  |  |
| Lynch CP, Williams JS, K JR, R GK, Egede LE. Tablet-Aided BehavioraL intervention EffecT on Self-management skills (TABLETS) for Diabetes. Trials. 2016;17:157. |  |  | x |  |  |  |
| Madden MH, Tomsik P, Terchek J, Navracruz L, Reichsman A, Clark TC, et al. Keys to successful diabetes self-management for uninsured patients: social support, observational learning, and turning points: a safety net providers' strategic alliance study. Journal of the National Medical Association. 2011;103(3):257-64. |  |  |  |  | x |  |
| Maglalang DD, Yoo GJ, Ursua RA, Villanueva C, Chesla CA, Bender MS. "I DON'T HAVE TO EXPLAIN, PEOPLE UNDERSTAND": ACCEPTABILITY AND CULTURAL RELEVANCE OF A MOBILE HEALTH LIFESTYLE INTERVENTION FOR FILIPINOS WITH TYPE 2 DIABETES. Ethnicity & disease. 2017;27(2):143-54. |  |  |  |  | x |  |
| Maine A, Dickson A, Truesdale M, Brown M. An application of Bandura's ‘Four Sources of Self-Efficacy’ to the self-management of type 2 diabetes in people with intellectual disability: An inductive and deductive thematic analysis. Research in Developmental Disabilities. 2017;70:75-84. |  |  |  |  | x |  |
| Majeed-Ariss R, Jackson C, Knapp P, Cheater FM. British-Pakistani women's perspectives of diabetes self-management: the role of identity. Journal of clinical nursing. 2015;24(17-18):2571-80. |  |  |  |  | x |  |
| Majeed-Ariss R, Jackson C, Knapp P, Cheater FM. A systematic review of research into black and ethnic minority patients' views on self-management of type 2 diabetes. Health Expectations. 2015;18(5):625-42. |  |  |  |  | x |  |
| Makowski AC, Kofahl C. Benefit and Adherence of the Disease Management Program "Diabetes 2": A Comparison of Turkish Immigrants and German Natives with Diabetes. International journal of environmental research and public health. 2014;11(9):9724-39. |  |  |  |  | x |  |
| Manley D, Garth K, Byers D. Challenges in recruitment of African Americans with type 2 diabetes in rural settings. Kentucky nurse. 2012;60(4):5. | x |  |  |  |  |  |
| Mansyur CL, Rustveld LO, Nash SG, Jibaja-Weiss ML. Social factors and barriers to self-care adherence in Hispanic men and women with diabetes. Patient education and counseling. 2015;98(6):805-10. |  |  |  |  | x |  |
| Marcy TR, Britton ML, Harrison D. Identification of barriers to appropriate dietary behavior in low-income patients with type 2 diabetes mellitus. Diabetes Therapy. 2011;2(1):9-19. |  |  |  |  | x |  |
| Marquez B, Anderson A, Wing RR, West DS, Newton RL, Meacham M, et al. The relationship of social support with treatment adherence and weight loss in Latinos with type 2 diabetes. Obesity (Silver Spring, Md). 2016;24(3):568-75. |  |  |  |  | x |  |
| Marquez DX, Bustamante EE, Bock BC, Markenson G, Tovar A, Chasan-Taber L. Perspectives of Latina and non-Latina white women on barriers and facilitators to exercise in pregnancy. Women & health. 2009;49(6):505-21. |  |  |  |  | x |  |
| Martinez J, Powell J, Agne A, Scarinci I, Cherrington A. A focus group study of Mexican immigrant men's perceptions of weight and lifestyle. Public health nursing (Boston, Mass). 2012;29(6):490-8. |  |  |  |  | x |  |
| Martinez NG, Niznik CM, Yee LM. Optimizing postpartum care for the patient with gestational diabetes mellitus. American journal of obstetrics and gynecology. 2017;217(3):314-21. |  |  | x |  |  |  |
| Martis R, Brown J, McAra-Couper J, Crowther CA. Enablers and barriers for women with gestational diabetes mellitus to achieve optimal glycaemic control - a qualitative study using the theoretical domains framework. BMC pregnancy and childbirth. 2018;18. |  |  | x |  |  |  |
| Mathiesen AS, Thomsen T, Jensen T, Schiøtz C, Langberg H, Egerod I. The influence of diabetes distress on digital interventions for diabetes management in vulnerable people with type 2 diabetes: A qualitative study of patient perspectives. Journal of Clinical and Translational Endocrinology. 2017;9:41-7. |  |  | x |  |  |  |
| May KM, Rew L. Mexican American youths' and mothers' explanatory models of diabetes prevention. Journal for specialists in pediatric nursing: JSPN. 2010;15(1):6-15. |  |  | x |  |  |  |
| Mayberry LS, Berg CA, Harper KJ, Osborn CY. The Design, Usability, and Feasibility of a Family-Focused Diabetes Self-Care Support mHealth Intervention for Diverse, Low-Income Adults with Type 2 Diabetes. Journal of diabetes research. 2016. |  |  |  |  | x |  |
| Mayberry LS, Harper KJ, Osborn CY. Family behaviors and type 2 diabetes: What to target and how to address in interventions for adults with low socioeconomic status. Chronic illness. 2016;12(3):199-215. |  |  |  | x |  |  |
| McCarthy EA, Walker SP, Ugoni A, Lappas M, Leong O, Shub A. Self-weighing and simple dietary advice for overweight and obese pregnant women to reduce obstetric complications without impact on quality of life: a randomised controlled trial. Bjog-an International Journal of Obstetrics and Gynaecology. 2016;123(6):965-73. |  |  | x |  |  |  |
| McCloskey J. Promotores as partners in a community-based diabetes intervention program targeting Hispanics. Family & community health. 2009;32(1):48-57. |  | x |  |  |  |  |
| McCurley JL, Gutierrez AP, Gallo LC. Diabetes Prevention in U.S. Hispanic Adults: A Systematic Review of Culturally Tailored Interventions. American journal of preventive medicine. 2017;52(4):519-29. |  |  |  |  | x |  |
| McEwen MM, Murdaugh C. Partnering With Families to Refine and Expand a Diabetes Intervention for Mexican Americans. The Diabetes educator. 2014;40(4):488-95. |  |  |  |  | x |  |
| McLaughlin RA. Associations among health literacy levels and health outcomes in pregnant women with pregestational and gestational diabetes in an urban setting: University of Tennessee Health Science Center; 2009. |  |  |  | x |  |  |
| Mendelson SG, McNeese-Smith D, Koniak-Griffin D, Nyamathi A, Lu MC. A community-based parish nurse intervention program for Mexican American women with gestational diabetes. Journal of obstetric, gynecologic, and neonatal nursing : JOGNN. 2008;37(4):415-25. |  |  |  |  | x |  |
| Mersereau P, Williams J, Collier SA, Mulholland C, Turay K, Prue C. Barriers to managing diabetes during pregnancy: The perceptions of health care practitioners. Birth: Issues in Perinatal Care. 2011;38(2):142-9. |  | x |  |  |  |  |
| Metghalchi S, Rivera M, Beeson L, Firek A, De Leon M, Balcazar H, et al. Improved clinical outcomes using a culturally sensitive diabetes education program in a Hispanic population. The Diabetes educator. 2008;34(4):698-706. |  |  |  |  | x |  |
| Mian SI, Brauer PM. Dietary education tools for South Asians with diabetes. Canadian journal of dietetic practice and research: a publication of Dietitians of Canada = Revue canadienne de la pratique et de la recherche en dietetique : une publication des Dietetistes du Canada. 2009;70(1):28-35. |  |  |  |  | x |  |
| Mier N, Wang XH, Smith ML, Irizarry D, Trevino L, Alen M, et al. Factors Influencing Health Care Utilization in Older Hispanics with Diabetes along the Texas-Mexico Border. Population health management. 2012;15(3):149-56. |  |  |  |  | x |  |
| Mikell M. Exploring Factors Influencing Health Promoting Behaviors Among Latino Immigrants. Exploring Factors Influencing Health Promoting Behaviors Among Latino Immigrants. 2017:1-. | x |  |  |  |  |  |
| Millard AV, Graham MA, Wang XH, Mier N, Sanchez ER, Flores I, et al. Pilot of a Diabetes Primary Prevention Program in a Hard-to-Reach, Low-Income, Immigrant Hispanic Population. Journal of immigrant and minority health. 2011;13(5):906-13. |  |  |  |  | x |  |
| Miller ST, Akohoue SA, Brooks MA. Identification of patient-centered outcomes among African American women with type 2 diabetes. Diabetes research and clinical practice. 2014;106(3):487-90. |  |  |  | x |  |  |
| Miller ST, Beech BM. Rural healthcare providers question the practicality of motivational interviewing and report varied physical activity counseling experience. Patient education and counseling. 2009;76(2):279-82. |  | x |  |  |  |  |
| Miller ST, Marolen K. Physical activity-related experiences, counseling expectations, personal responsibility, and altruism among urban African American women with type 2 diabetes. The Diabetes educator. 2012;38(2):229-35. |  |  |  |  | x |  |
| Miller ST, Marolen KN, Beech BM. Perceptions of physical activity and motivational interviewing among rural African-American women with type 2 diabetes. Women's health issues: official publication of the Jacobs Institute of Women's Health. 2010;20(1):43-9. |  |  |  |  | x |  |
| Miskovich L. Interventions to improve the management of medically uninsured adult patients with type 2 diabetes mellitus in primary care, community-based settings. Western journal of nursing research. 2011;33(8):1112-3. |  |  |  |  | x |  |
| Mitchell JA, Hawkins J. Current approaches to support the psychosocial care of African American adults with diabetes: a brief review. Social work in public health. 2014;29(6):518-27. |  |  |  |  |  | x |
| Mitchell-Brown F, Nemeth L, Cartmell K, Newman S, Goto K. A Study of Hmong Immigrants’ Experience With Diabetes Education: A Community-Engaged Qualitative Study. Journal of Transcultural Nursing. 2017;28(6):540-9. |  |  |  |  | x |  |
| Moise RK, Conserve DF, Elewonibi B, Francis LA, BeLue R. Diabetes Knowledge, Management, and Prevention Among Haitian Immigrants in Philadelphia. The Diabetes educator. 2017;43(4):341-7. |  |  |  |  | x |  |
| Montgomery M, Johnson P, Ewell P. The Presence of Risk Factors for Type 2 Diabetes Mellitus in Underserved Preschool Children. The Nursing clinics of North America. 2015;50(3):585-94. |  |  | x |  |  |  |
| Moore SR. Cultural beliefs, meanings and preventive practices among African American women at risk for type II diabetes. Cultural Beliefs, Meanings & Preventive Practices Among African American Women at Risk for Type II Diabetes. 2016:1-. | x |  |  |  |  |  |
| Mora N, Golden SH. Understanding Cultural Influences on Dietary Habits in Asian, Middle Eastern, and Latino Patients with Type 2 Diabetes: A Review of Current Literature and Future Directions. Current diabetes reports. 2017;17(12):126. |  |  |  |  | x |  |
| Moreno G, Morales LS, de Jaimes FN, Tseng CH, Isiordia M, Noguera C, et al. Neighborhood Perceptions and Health-Related Outcomes Among Latinos with Diabetes from a Rural Agricultural Community. Journal of community health. 2014;39(6):1077-84. |  |  |  |  | x |  |
| Morishita M, Hattori S, Miyai N. Ability for Self-Care among Elderly Patients with Diabetes Mellitus and Its Association with Health Locus of Control and Social Support. Nihon eiseigaku zasshi Japanese journal of hygiene. 2017;72(1):77-86. |  |  |  |  | x |  |
| Morris NS, Maclean CD, Littenberg B. Change in health literacy over 2 years in older adults with diabetes. The Diabetes educator. 2013;39(5):638-46. |  |  | x |  |  |  |
| Moussa M, Sherrod D, Choi J. An e-health intervention for increasing diabetes knowledge in African Americans. International journal of nursing practice. 2013;19 Suppl 3:36-43. |  | x |  |  |  |  |
| Mulvaney SA, Mudasiru E, Schlundt DG, Baughman CL, Fleming M, VanderWoude A, et al. Self-management in type 2 diabetes - The adolescent perspective. Diabetes Educator. 2008;34(4):674-82. |  |  | x |  |  |  |
| Murfin J, Hulson O, Rajeswaran C. Patients' understanding is the key to effective communication. Practical Diabetes International. 2009;26(7):262. |  |  | x |  |  |  |
| Murrock CJ, Gary FA. A culturally-specific dance intervention to increase functional capacity in African American women. Journal of cultural diversity. 2008;15(4):168-73. |  | x |  |  |  |  |
| Murrock CJ, Higgins PA, Killion C. Dance and Peer Support to Improve Diabetes Outcomes in African American Women. Diabetes Educator. 2009;35(6):995-1003. |  |  |  |  | x |  |
| Murrock CJ, Taylor E, Marino D. Dietary challenges of managing type 2 diabetes in African-American women. Women & health. 2013;53(2):173-84. |  |  |  |  | x |  |
| Nam S, Janson SL, Stotts NA, Chesla C, Kroon L. Effect of culturally tailored diabetes education in ethnic minorities with type 2 diabetes: a meta-analysis. The Journal of cardiovascular nursing. 2012;27(6):505-18. |  |  |  | x |  |  |
| Nam S, Song H-J, Park S-Y, Song Y. Challenges of diabetes management in immigrant Korean Americans. The Diabetes educator. 2013;39(2):213-21. |  |  |  |  | x |  |
| Newlin K, Dyess SM, Allard E, Chase S, Melkus GD. A methodological review of faith-based health promotion literature: advancing the science to expand delivery of diabetes education to Black Americans. Journal of religion and health. 2012;51(4):1075-97. |  |  |  |  | x |  |
| Newlin Lew K, Arbauh N, Banach P, Melkus G. Diabetes: Christian worldview, medical distrust and self-management. Journal of religion and health. 2015;54(3):1157-72. |  | x |  |  |  |  |
| Newton P, Asimakopoulou K, Scambler S. Information seeking and use amongst people living with type 2 diabetes: An information continuum. International Journal of Health Promotion and Education. 2012;50(2):92-9. |  |  | x |  |  |  |
| Newton P, Asimakopoulou K, Scambler S. A Qualitative Exploration of Motivation to Self-Manage and Styles of Self-Management amongst People Living with Type 2 Diabetes. Journal of diabetes research. 2015. |  |  | x |  |  |  |
| Ngo-Metzger Q, Sorkin DH, Billimek J, Greenfield S, Kaplan SH. The effects of financial pressures on adherence and glucose control among racial/ethnically diverse patients with diabetes. Journal of general internal medicine. 2012;27(4):432-7. |  |  |  |  | x |  |
| Nguyen AL, Sepulveda E, Angulo M. "It Feels Good to Know That Someone Cares". Hispanic health care international: the official journal of the National Association of Hispanic Nurses. 2017;15(2):52-7. | x |  |  |  |  |  |
| Nicklas JM, Zera CA, Seely EW, Abdul-Rahim ZS, Rudloff ND, Levkoff SE. Identifying postpartum intervention approaches to prevent type 2 diabetes in women with a history of gestational diabetes. BMC pregnancy and childbirth. 2011;11. |  |  | x |  |  |  |
| Njeru JW, Patten CA, Hanza MM, Brockman TA, Ridgeway JL, Weis JA, et al. Stories for change: development of a diabetes digital storytelling intervention for refugees and immigrants to minnesota using qualitative methods. BMC public health. 2015;15:1311. |  |  |  |  | x |  |
| Ntiri DW, Stewart M. Transformative learning intervention: effect on functional health literacy and diabetes knowledge in older African Americans. Gerontology & geriatrics education. 2009;30(2):100-13. |  |  |  | x |  |  |
| Oakes AH, Garmo VS, Bone LR, Longo DR, Segal JB, Bridges JFP. Identifying and Prioritizing the Barriers and Facilitators to the Self-Management of Type 2 Diabetes Mellitus: A Community-Centered Approach. The patient. 2017;10(6):773-83. |  |  | x |  |  |  |
| O'Brien MJ, Moran MR, Tang JW, Vargas MC, Talen M, Zimmermann LJ, et al. Patient Perceptions About Prediabetes and Preferences for Diabetes Prevention. The Diabetes educator. 2016;42(6):667-77. |  |  | x |  |  |  |
| O'Brien MJ, Perez A, Alos VA, Whitaker RC, Ciolino JD, Mohr DC, et al. The feasibility, acceptability, and preliminary effectiveness of a Promotora-Led Diabetes Prevention Program (PL-DPP) in Latinas: a pilot study. The Diabetes educator. 2015;41(4):485-94. |  |  |  |  | x |  |
| O'Brien MJ, Shuman SJ, Barrios DM, Alos VA, Whitaker RC. A qualitative study of acculturation and diabetes risk among urban immigrant Latinas: implications for diabetes prevention efforts. The Diabetes educator. 2014;40(5):616-25. |  |  |  |  | x |  |
| Okrainec K, Booth GL, Hollands S, Bell CM. Impact of language barriers on complications and mortality among immigrants with diabetes: a population-based cohort study. Diabetes care. 2015;38(2):189-96. |  |  |  | x |  |  |
| Oliveros J, Yukihiro D, Segura PM, Lillington LM. Are Postpartum Women with Gestational Diabetes Mellitus Aware of Their Risk?...Proceedings of the 2015 AWHONN Convention. JOGNN: Journal of Obstetric, Gynecologic & Neonatal Nursing. 2015;44:S67-S. |  |  |  |  |  |  |
| Olson E, McAuley E. Impact of a brief intervention on self-regulation, self-efficacy and physical activity in older adults with type 2 diabetes. Journal of behavioral medicine. 2015;38(6):886-98. |  |  |  |  | x |  |
| O'Mara B, Gill GK, Babacan H, Donahoo D. Digital technology, diabetes and culturally and linguistically diverse communities: A case study with elderly women from the Vietnamese community. Health Education Journal. 2012;71(4):491-504. |  |  |  |  | x |  |
| Osborn CY, Amico KR, Cruz N, O'Connell AA, Perez-Escamilla R, Kalichman SC, et al. A brief culturally tailored intervention for Puerto Ricans with type 2 diabetes. Health education & behavior: the official publication of the Society for Public Health Education. 2010;37(6):849-62. |  |  |  |  | x |  |
| Osborn CY, Amico KR, Cruz N, Perez-Escamilla R, Kalichman SC, O'Connell AA, et al. Development and implementation of a culturally tailored diabetes intervention in primary care. Translational behavioral medicine. 2011;1(3):468-79. |  |  |  |  | x |  |
| Osman A, Curzio J. South Asian cultural concepts in diabetes. Nursing Times. 2012;108(10):28-32. |  |  |  |  | x |  |
| Osuna D, Barrera M, Jr., Strycker LA, Toobert DJ, Glasgow RE, Geno CR, et al. Methods for the cultural adaptation of a diabetes lifestyle intervention for Latinas: an illustrative project. Health promotion practice. 2011;12(3):341-8. |  |  |  |  | x |  |
| Otero-Sabogal R, Arretz D, Siebold S, Hallen E, Lee R, Ketchel A, et al. Physician-community health worker partnering to support diabetes self-management in primary care. Quality in primary care. 2010;18(6):363-72. |  |  |  |  | x |  |
| Oza-Frank R, Conrey E, Bouchard J, Shellhaas C, Weber MB. Healthcare Experiences of Low-Income Women with Prior Gestational Diabetes. Maternal and child health journal. 2018. |  |  |  |  | x |  |
| Parchman ML, Flannagan D, Ferrer RL, Matamoras M. Communication competence, self-care behaviors and glucose control in patients with type 2 diabetes. Patient education and counseling. 2009;77(1):55-9. |  |  | x |  |  |  |
| Park C, Nam S, Whittemore R. Incorporating Cultural Perspectives into Diabetes Self-Management Programs for East Asian Immigrants: A Mixed-Study Review. Journal of immigrant and minority health. 2016;18(2):454-67. |  |  |  |  | x |  |
| Parken H, Sturt J. Ongoing education in type 2 diabetes: The attitudes of hard-to-reach participants. Primary Health Care Research and Development. 2009;10(1):38-48. |  |  | x |  |  |  |
| Parker MM, Fernández A, Moffet HH, Grant RW, Torreblanca A, Karter AJ. Association of Patient-Physician Language Concordance and Glycemic Control for Limited-English Proficiency Latinos With Type 2 Diabetes. JAMA internal medicine. 2017;177(3):380-7. |  |  |  |  | x |  |
| Parsons J, Sparrow K, Ismail K, Hunt K, Rogers H, Forbes A. Experiences of gestational diabetes and gestational diabetes care: a focus group and interview study. BMC pregnancy and childbirth. 2018;18. |  |  | x |  |  |  |
| Patel N, Ferrer HB, Tyrer F, Wray P, Farooqi A, Davies MJ, et al. Barriers and Facilitators to Healthy Lifestyle Changes in Minority Ethnic Populations in the UK: a Narrative Review. Journal of racial and ethnic health disparities. 2017;4(6):1107-19. |  |  |  |  | x |  |
| Patel V, Iliffe S. An exploratory study into the health beliefs and behaviours of British Indians with type II diabetes. Primary Health Care Research and Development. 2017;18(1):97-103. |  |  |  |  | x |  |
| Patel V, Rajpathak S, Karasz A. Bangladeshi Immigrants in New York City: A Community Based Health Needs Assessment of a Hard to Reach Population. Journal of Immigrant & Minority Health. 2012;14(5):767-73. |  |  |  |  | x |  |
| Peek ME, Cargill A, Huang ES. Diabetes Health Disparities. Medical Care Research and Review. 2007;64(5_suppl):101S-56S. |  |  |  | x |  |  |
| Peeters B, Van Tongelen I, Duran Z, Yüksel G, Mehuys E, Willems S, et al. Understanding medication adherence among patients of Turkish descent with type 2 diabetes: a qualitative study. Ethnicity & health. 2015;20(1):87-105. |  |  |  | x |  |  |
| Penn L, Dombrowski SU, Sniehotta FF, White M. Participants' perspectives on making and maintaining behavioural changes in a lifestyle intervention for type 2 diabetes prevention: a qualitative study using the theory domain framework. BMJ open. 2013;3(6). |  |  | x |  |  |  |
| Penn L, Dombrowski SU, Sniehotta FF, White M. Perspectives of UK Pakistani women on their behaviour change to prevent type 2 diabetes: qualitative study using the theory domain framework. BMJ open. 2014;4(7):e004530. |  |  |  |  | x |  |
| Pesantes MA, Lazo-Porras M, Abu Dabrh AM, Avila-Ramirez JR, Caycho M, Villamonte GY, et al. Resilience in Vulnerable Populations With Type 2 Diabetes Mellitus and Hypertension: A Systematic Review and Meta-analysis. The Canadian journal of cardiology. 2015;31(9):1180-8. |  |  |  | x |  |  |
| Phelan S. Windows of Opportunity for Lifestyle Interventions to Prevent Gestational Diabetes Mellitus. American journal of perinatology. 2016;33(13):1291-9. |  |  | x |  |  |  |
| Philis-Tsimikas A, Fortmann AL, Dharkar-Surber S, Euyoque JA, Ruiz M, Schultz J, et al. Dulce Mothers: an intervention to reduce diabetes and cardiovascular risk in Latinas after gestational diabetes. Translational behavioral medicine. 2014;4(1):18-25. |  |  |  |  | x |  |
| Pilkington FB, Daiski I, Bryant T, Dinca-Panaitescu M, Dinca-Panaitescu S, Raphael D. The experience of living with diabetes for low-income Canadians. Canadian journal of diabetes. 2010;34(2):119-26. |  |  |  |  | x |  |
| Pilkington FB, Daiski I, Lines E, Bryant T, Raphael D, Dinca-Panaitescu M, et al. Type 2 diabetes in vulnerable populations: community healthcare providers' perspectives on health service needs and policy implications. Canadian journal of diabetes. 2011;35(5):503-11. |  | x |  |  |  |  |
| Pistulka GM, Winch PJ, Park H, Han HR, Kim MT. Maintaining an Outward Image: A Korean Immigrant's Life With Type 2 Diabetes Mellitus and Hypertension. Qualitative health research. 2012;22(6):825-34. |  |  |  | x |  |  |
| Piven EF. Activity and Occupational Demands of Type Two Diabetes: The Voice of Mexican-American Older Adults. Physical & Occupational Therapy in Geriatrics. 2015;33(1):34-52. |  |  |  |  |  |  |
| Piziak V. A pilot study of a pictorial bilingual nutrition education game to improve the consumption of healthful foods in a head start population. International journal of environmental research and public health. 2012;9(4):1319-25. |  |  | x |  |  |  |
| Piziak V. The development of a bilingual interactive video to improve physical activity and healthful eating in a head start population. International journal of environmental research and public health. 2014;11(12):13065-73. |  |  | x |  |  |  |
| Plasencia J, Hoerr S, Carolan M, Weatherspoon L. Acculturation and Self-Management Perceptions Among Mexican American Adults With Type 2 Diabetes. Family & community health. 2017;40(2):121-31. |  |  |  |  | x |  |
| Porteous HE, Palmer MA, Wilkinson SA. Informing maternity service development by surveying new mothers about preferences for nutrition education during their pregnancy in an area of social disadvantage. Women and birth: journal of the Australian College of Midwives. 2014;27(3):196-201. |  |  |  |  | x |  |
| Porter SJ, Chapman-Novakofski KM, Scherer JA. Your Guide to Diet and Diabetes: web-based diabetes education tailored to Hispanics. Journal of nutrition education and behavior. 2009;41(5):374-6. |  |  |  | x |  |  |
| Power ML, Wilson EK, Hogan SO, Loft JD, Williams JL, Mersereau PW, et al. Patterns of preconception, prenatal and postnatal care for diabetic women by obstetrician-gynecologists. The Journal of reproductive medicine. 2013;58(1-2):7-14. |  | x |  |  |  |  |
| Prezio EA, Cheng D, Balasubramanian BA, Shuval K, Kendzor DE, Culica D. Community Diabetes Education (CoDE) for uninsured Mexican Americans: a randomized controlled trial of a culturally tailored diabetes education and management program led by a community health worker. Diabetes research and clinical practice. 2013;100(1):19-28. |  |  |  |  | x |  |
| Purcell N, Cutchen L. Diabetes Self-Management Education for African Americans: Using the PEN-3 Model to Assess Needs. American Journal of Health Education. 2013;44(4):203-12. |  |  |  |  | x |  |
| Pyatak EA, Florindez D, Peters AL, Weigensberg MJ. "We Are All Gonna Get Diabetic These Days" The Impact of a Living Legacy of Type 2 Diabetes on Hispanic Young Adults' Diabetes Care. Diabetes Educator. 2014;40(5):648-58. |  |  |  |  | x |  |
| Raberg Kjollesdal MK, Hjellset VT, Bjorge B, Holmboe-Ottesen G, Wandel M. Perceptions of risk factors for diabetes among Norwegian-Pakistani women participating in a culturally adapted intervention. Ethnicity & health. 2011;16(3):279-97. |  |  |  |  | x |  |
| Råberg Kjøllesdal MK, Hjellset VT, Bjørge B, Holmboe-Ottesen G, Wandel M. Barriers to healthy eating among Norwegian-Pakistani women participating in a culturally adapted intervention. Scandinavian journal of public health. 2010;38(S5):52-9. |  |  |  |  | x |  |
| Rahim-Williams B. Beliefs, behaviors, and modifications of type 2 diabetes self-management among African American women. Journal of the National Medical Association. 2011;103(3):203-15. |  |  |  |  | x |  |
| Ramal E, Petersen AB, Ingram KM, Champlin AM. Factors that influence diabetes self-management in Hispanics living in low socioeconomic neighborhoods in San Bernardino, California. Journal of immigrant and minority health. 2012;14(6):1090-6. |  |  |  |  | x |  |
| Raphael D, Daiski I, Pilkington B, Bryant T, Dinca-Panaitescu M, Dinca-Panaitescu S. A toxic combination of poor social policies and programmes, unfair economic arrangements and bad politics: the experiences of poor Canadians with Type 2 diabetes. Critical Public Health. 2012;22(2):127-45. |  |  |  |  | x |  |
| Reinschmidt KM, Teufel-Shone NI, Bradford G, Drummond RL, Torres E, Redondo F, et al. Taking a broad approach to public health program adaptation: adapting a family-based diabetes education program. The journal of primary prevention. 2010;31(1-2):69-83. |  |  |  |  | x |  |
| Rendle KAS, May SG, Uy V, Tietbohl CK, Mangione CM, Frosch DL. Persistent barriers and strategic practices: Why (asking about) the everyday matters in diabetes care. The Diabetes educator. 2013;39(4):560-7. |  |  |  |  | x |  |
| Renzaho AM, Skouteris H, Oldroyd J. Preventing gestational diabetes mellitus among migrant women and reducing obesity and type 2 diabetes in their offspring: a call for culturally competent lifestyle interventions in pregnancy. Journal of the American Dietetic Association. 2010;110(12):1814-7. |  |  |  |  | x |  |
| Rhoads-Baeza ME, Reis J. An exploratory mixed method assessment of low income, pregnant Hispanic women's understanding of gestational diabetes and dietary change. Health Education Journal. 2012;71(1):80-9. |  |  |  |  | x |  |
| Ricci-Cabello I, Ruiz-Perez I, Rojas-Garcia A, Pastor G, Rodriguez-Barranco M, Goncalves DC. Characteristics and effectiveness of diabetes self-management educational programs targeted to racial/ethnic minority groups: a systematic review, meta-analysis and meta-regression. BMC endocrine disorders. 2014;14:60. |  |  |  |  | x |  |
| Richardson BS, Willig AL, Agne AA, Cherrington AL. Diabetes connect: African American women’s perceptions of the community health worker model for diabetes care. Journal of Community Health: The Publication for Health Promotion and Disease Prevention. 2015;40(5):905-11. |  |  |  |  | x |  |
| Rogers EA, Fine S, Handley MA, Davis H, Kass J, Schillinger D. Development and early implementation of the bigger picture, a youth-targeted public health literacy campaign to prevent type 2 diabetes. Journal of health communication. 2014;19 Suppl 2:144-60. |  |  | x |  |  |  |
| Rogers EA, Fine SC, Handley MA, Davis HB, Kass J, Schillinger D. Engaging Minority Youth in Diabetes Prevention Efforts Through a Participatory, Spoken-Word Social Marketing Campaign. American journal of health promotion : AJHP. 2017;31(4):336-9. |  |  | x |  |  |  |
| Rogers EA, Hessler DM, Bodenheimer TS, Ghorob A, Vittinghoff E, Thom DH. Diabetes peer coaching: do "better patients" make better coaches? The Diabetes educator. 2014;40(1):107-15. |  |  |  |  | x |  |
| Rosal MC, Borg A, Bodenlos JS, Tellez T, Ockene IS. Awareness of diabetes risk factors and prevention strategies among a sample of low-income Latinos with no known diagnosis of diabetes. The Diabetes educator. 2011;37(1):47-55. |  |  |  |  | x |  |
| Rose VK, Harris MF. Experiences of self-management support from GPs among Australian ethnically diverse diabetes patients: A qualitative study. Psychology, health & medicine. 2015;20(1):114-20. |  |  |  | x |  |  |
| Ruddock JS, Poindexter M, Gary-Webb TL, Walker EA, Davis NJ. Innovative strategies to improve diabetes outcomes in disadvantaged populations. Diabetic medicine : a journal of the British Diabetic Association. 2016;33(6):723-33. |  |  |  |  | x |  |
| Samuel-Hodge CD, Holder-Cooper JC, Gizlice Z, Davis G, Steele SP, Keyserling TC, et al. Family PArtners in Lifestyle Support (PALS): Family-Based Weight Loss for African American Adults with Type 2 Diabetes. Obesity. 2017;25(1):45-55. |  |  |  |  | x |  |
| Sanders LM, Shaw JS, Guez G, Baur C, Rudd R. Health literacy and child health promotion: implications for research, clinical care, and public policy. Pediatrics. 2009;124 Suppl 3:S306-14. |  |  | x |  |  |  |
| Sattin RW, Williams LB, Dias J, Garvin JT, Marion L, Joshua TV, et al. Community Trial of a Faith-Based Lifestyle Intervention to Prevent Diabetes Among African-Americans. Journal of community health. 2016;41(1):87-96. |  |  |  |  | x |  |
| Savage C, Xu Y, Richmond MM, Corbin A, Falciglia M, Gillespie G. A Pilot Study: Retention of Adults Experiencing Homelessness and Feasibility of a CDSM Diabetes Program. Journal of community health nursing. 2014;31(4):238-48. |  |  |  |  | x |  |
| Sawyer MT, Deines CK. Missing the mark with Latina women with type 2 diabetes: implications for educators. The Diabetes educator. 2013;39(5):671-8. |  |  |  |  | x |  |
| Sayah FA, Majumdar SR, Egede LE, Johnson JA. Associations between health literacy and health outcomes in a predominantly low-income African American population with type 2 diabetes. Journal of health communication. 2015;20(5):581-8. |  | x |  |  |  |  |
| Schillinger D, Tran J, Fine S. Do Low Income Youth of Color See "The Bigger Picture" When Discussing Type 2 Diabetes: A Qualitative Evaluation of a Public Health Literacy Campaign. International journal of environmental research and public health. 2018;15(5). |  |  | x |  |  |  |
| Scollan-Koliopoulos M, Rapp KJ, Bleich D. Afrocentric Cultural Values and Beliefs Movement Beyond the Race and Ethnicity Proxy to Understand Views of Diabetes. Diabetes Educator. 2012;38(4):488-98. |  |  | x |  |  |  |
| Seale C, Rivas C, Al-Sarraj H, Webb S, Kelly M. Moral mediation in interpreted health care consultations. Social science & medicine (1982). 2013;98:141-8. |  |  |  |  | x |  |
| Shacter HE, Shea JA, Akhabue E, Sablani N, Long JA. A qualitative evaluation of racial disparities in glucose control. Ethnicity & disease. 2009;19(2):121-7. |  |  |  |  | x |  |
| Shah M, Kaselitz E, Heisler M. The Role of Community Health Workers in Diabetes: Update on Current Literature. Current diabetes reports. 2013;13(2):163-71. |  | x |  |  |  |  |
| Shah MK, Kieffer EC, Choi H, Schumann C, Heisler M. Mediators and Moderators of the Effectiveness of a Community Health Worker Intervention That Improved Dietary Outcomes in Pregnant Latino Women. Health Education & Behavior. 2015;42(5):593-603. |  |  |  |  | x |  |
| Shahady EJ. Barriers to effective diabetes care: How to recognize and overcome. Consultant. 2009;49(8). |  |  |  |  |  |  |
| Shaibi GQ, Konopken Y, Hoppin E, Keller CS, Ortega R, Castro FG. Effects of a culturally grounded community-based diabetes prevention program for obese Latino adolescents. The Diabetes educator. 2012;38(4):504-12. |  |  | x |  |  |  |
| Shaibi GQ, Konopken YP, Nagle-Williams A, McClain DD, Castro FG, Keller CS. Diabetes Prevention for Latino Youth: Unraveling the Intervention "Black Box". Health promotion practice. 2015;16(6):916-24. |  |  | x |  |  |  |
| Shrestha A, Karmacharya BM, Khudyakov P, Weber MB, Spiegelman D. Dietary interventions to prevent and manage diabetes in worksite settings: a meta-analysis. Journal of Occupational Health. 2018;60(1):31-45. |  |  | x |  |  |  |
| Shultz JA, Corbett CF, Allen CB. Slavic Women's Understanding of Diabetes Dietary Self-Management and Reported Dietary Behaviors. Journal of immigrant and minority health. 2009;11(5):400-5. |  |  |  |  | x |  |
| Singh H, Cinnirella M, Bradley C. Support systems for and barriers to diabetes management in South Asians and Whites in the UK: Qualitative study of patients' perspectives. BMJ open. 2012;2(6). |  |  |  |  | x |  |
| Sixta CS, Ostwald S. Strategies for implementing a promotores-led diabetes self-management program into a clinic structure. The Diabetes educator. 2008;34(2):285-98. |  |  |  |  | x |  |
| Smith-Miller CA, Berry DC, Miller CT. Diabetes affects everything: Type 2 diabetes self-management among Spanish-speaking hispanic immigrants. Research in nursing & health. 2017;40(6):541-54. |  |  |  |  | x |  |
| Sohal PS. Prevention and management of diabetes in South Asians. Canadian journal of diabetes. 2008;32(3):206-10. |  |  |  |  | x |  |
| Soltero EG, Konopken YP, Olson ML, Keller CS, Castro FG, Williams AN, et al. Preventing diabetes in obese Latino youth with prediabetes: a study protocol for a randomized controlled trial. BMC public health. 2017;17(1):261. |  |  | x |  |  |  |
| Song H-J, Han H-R, Lee J-E, Kim J, Kim KB, Nguyen T, et al. Translating current dietary guidelines into a culturally tailored nutrition education program for Korean American immigrants with type 2 diabetes. The Diabetes educator. 2010;36(5):752-61. |  |  |  |  | x |  |
| Song Y, Song HJ, Han HR, Park SY, Nam S, Kim MT. Unmet Needs for Social Support and Effects on Diabetes Self-care Activities in Korean Americans With Type 2 Diabetes. Diabetes Educator. 2012;38(1):77-85. |  |  |  |  | x |  |
| Sorkin DH, Biegler KA, Peyreda M, Kilgore D, Dow E, Ngo-Metzger Q. Unidas por la Vida (United for Life): implementing a culturally-tailored, community-based, family-oriented lifestyle intervention. Journal of health care for the poor and underserved. 2013;24(2 Suppl):116-38. |  |  |  | x |  |  |
| Soto SC, Louie SY, Cherrington AL, Parada H, Horton LA, Ayala GX. An ecological perspective on diabetes self-care support, self-management behaviors, and hemoglobin A1C among Latinos. The Diabetes educator. 2015;41(2):214-23. |  |  |  |  | x |  |
| Stone MA, Patel N, Daly H, Martin-Stacey L, Amin S, Carey M, et al. Using qualitative research methods to inform the development of a modified version of a patient education module for non-English speakers with type 2 diabetes: experience from an action research project in two South Asian populations in the UK. Diversity in Health & Social Care. 2008;5(3):199-206. |  |  |  |  | x |  |
| Sullivan LV, Hicks P, Salazar G, Robinson CK. Patient beliefs and sense of control among Spanish-speaking patients with diabetes in northeast Colorado. Journal of immigrant and minority health. 2010;12(3):384-9. |  |  |  | x | x |  |
| Sumlin LL. Culture and Food Practices of African-American Women with Type 2 Diabetes: University of Texas at Austin; 2014. |  |  |  | x |  |  |
| Szczepura A. Nutrition in an ethnically diverse society: what are some of the key challenges? The Proceedings of the Nutrition Society. 2011;70(2):252-62. |  |  |  | x |  |  |
| Tan CCL, Cheng KKF, Sum CF, Shew JSH, Holydard E, Wang W. Perceptions of Diabetes Self-Care Management Among Older Singaporeans With Type 2 Diabetes: A Qualitative Study. The journal of nursing research : JNR. 2017. | x |  |  |  |  |  |
| Tang J, Foster K, Pumarino J, Ackermann R, Peaceman A, Cameron K. Perspectives on Prevention of Type 2 Diabetes After Gestational Diabetes: A Qualitative Study of Hispanic, African-American and White Women. Maternal & Child Health Journal. 2015;19(7):1526-34. |  |  |  |  | x |  |
| Taylor J, Cottrell C, Chatterton H, Hill J, Hughes R, Wohlgemuth C, et al. Identifying risk and preventing progression to Type 2 diabetes in vulnerable and disadvantaged adults: a pragmatic review. Diabetic Medicine. 2013;30(1):16-25. |  |  | x |  |  |  |
| Thompson C, Meeuwisse I, Dahlke R, Drummond N. Group medical visits in primary care for patients with diabetes and low socioeconomic status: users' perspectives and lessons for practitioners. Canadian journal of diabetes. 2014;38(3):198-204. |  |  |  |  | x |  |
| Tomlin A, Asimakopoulou K. Supporting behaviour change in older people with type 2 diabetes. British journal of community nursing. 2014;19(1):22-7. |  |  |  | x |  |  |
| Tucker CM, Smith TM, Arthur TM, Wall W. Obesity and related chronic health conditions as predictors of motivation to engage in healthy eating behaviors among Black adults. Journal of racial and ethnic health disparities. 2014;1(2):102-9. |  |  |  | x |  |  |
| Valdez RS, Brennan PF. Embracing complexity: Rethinking culturally informed design in human factors/ergonomics and consumer health informatics. International Journal of Human-Computer Interaction. 2017;33(4):322-32. |  | x |  |  |  |  |
| Valen MS, Narayan S, Wedeking L. An innovative approach to diabetes education for a Hispanic population utilizing community health workers. Journal of cultural diversity. 2012;19(1):10-7. |  |  |  |  | x |  |
| van Esch SCM, Cornel MC, Geelhoed-Duijvestijn PHLM, Snoek FJ. Family communication as strategy in diabetes prevention: An observational study in families with Dutch and Surinamese South-Asian ancestry. Patient education and counseling. 2012;87(1):23-9. |  |  |  | x |  |  |
| Van Son CR. Developing Culturally Targeted Diabetes Educational Materials for Older Russian-Speaking Immigrants. The Diabetes educator. 2014;40(4):418-26. |  |  |  |  | x |  |
| Venditti EM. Behavioral lifestyle interventions for the primary prevention of type 2 diabetes and translation to Hispanic/Latino communities in the United States and Mexico. Nutrition reviews. 2017;75(suppl 1):85-93. |  |  |  |  |  | x |
| Venditti EM, Giles C, Firrell LS, Zeveloff AD, Hirst K, Marcus MD. Interactive learning activities for the middle school classroom to promote healthy energy balance and decrease diabetes risk in the HEALTHY primary prevention trial. Health promotion practice. 2014;15(1):55-62. |  |  | x |  |  |  |
| Vincent D. Culturally tailored education to promote lifestyle change in Mexican Americans with type 2 diabetes. Journal of the American Academy of Nurse Practitioners. 2009;21(9):520-7. |  |  |  |  | x |  |
| Vischer UM, Bauduceau B, Bourdel-Marchasson I, Blickle JF, Constans T, Fagot-Campagna A, et al. A call to incorporate the prevention and treatment of geriatric disorders in the management of diabetes in the elderly. Diabetes & metabolism. 2009;35(3):168-77. |  | x |  |  |  |  |
| Vissenberg C, Nierkens V, Uitewaal PJM, Geraci D, Middelkoop BJC, Nijpels G, et al. The DISC (Diabetes in Social Context) Study-evaluation of a culturally sensitive social network intervention for diabetic patients in lower socioeconomic groups: a study protocol. BMC public health. 2012;12. |  |  |  |  | x |  |
| Vissenberg C, Nierkens V, Uitewaal PJM, Middelkoop BJC, Stronks K. Recruitment and retention in a 10-month social network-based intervention promoting diabetes self-management in socioeconomically deprived patients: A qualitative process evaluation. BMJ open. 2017;7(7). |  |  |  |  | x |  |
| Vivian EM, Becker TL, Carrel AL. Weight perceptions of parents with children at risk for diabetes. BMC research notes. 2012;5:47. |  |  | x |  |  |  |
| Vivian EM, Carrel AL, Becker T. Identifying children at risk for type 2 diabetes in underserved communities. The Diabetes educator. 2011;37(4):519-27. |  |  | x |  |  |  |
| Wagner J, Berthold SM, Buckley T, Kong S, Kuoch T, Scully M. Diabetes among refugee populations: what newly arriving refugees can learn from resettled Cambodians. Current diabetes reports. 2015;15(8):56. |  |  |  |  | x |  |
| Wagner JA, Osborn CY, Mendenhall EA, Budris LM, Belay S, Tennen HA. Beliefs about racism and health among African American women with diabetes: a qualitative study. Journal of the National Medical Association. 2011;103(3):224-32. |  |  |  | x |  |  |
| Wagnild G, MacCart JG, Mitchell S, Tyabah K, Leenknecht C, Meszaros JF. A telecommunications intervention for frontier patients with diabetes. Telemedicine journal and e-health: the official journal of the American Telemedicine Association. 2008;14(8):793-800. |  |  | x |  |  |  |
| Wah YYE, McGill M, Wong J, Ross GP, Harding AJ, Krass I. Self-management of gestational diabetes among Chinese migrants: A qualitative study. Women and Birth. 2018. | x |  |  |  |  |  |
| Walker KC, Valentiner LS, Langberg H. Motivational factors for initiating, implementing, and maintaining physical activity behavior following a rehabilitation program for patients with type 2 diabetes: A longitudinal, qualitative, interview study. Patient preference and adherence. 2018;12:145-52. |  |  | x |  |  |  |
| Walpole B, Dettmer E, Morrongiello B, McCrindle B, Hamilton J. Motivational Interviewing as an intervention to increase adolescent self-efficacy and promote weight loss: Methodology and design. BMC public health. 2011;11. |  |  | x |  |  |  |
| Wang ML, Gallivan L, Lemon SC, Borg A, Ramirez J, Figueroa B, et al. Navigating to health: Evaluation of a community health center patient navigation program. Preventive Medicine Reports. 2015;2:664-8. |  |  |  |  | x |  |
| Wang X, Ghaddar S, Brown C, Pagan JA, Balboa M. Alliance for a Healthy Border: factors related to weight reduction and glycemic success. Population health management. 2012;15(2):90-100. |  |  |  |  | x |  |
| Wang Y, Chuang L, Bateman WB. Focus group study assessing self-management skills of Chinese Americans with type 2 diabetes mellitus. Journal of immigrant and minority health. 2012;14(5):869-74. |  |  |  |  | x |  |
| Wang-Letzkus MF, Washington G, Calvillo ER, Anderson NLR. Using Culturally Competent Community-Based Participatory Research With Older Diabetic Chinese Americans: Lessons Learned. Journal of Transcultural Nursing. 2012;23(3):255-61. |  |  |  |  | x |  |
| Watkins YJ, Quinn LT, Ruggiero L, Quinn MT, Choi YK. Spiritual and Religious Beliefs and Practices and Social Support's Relationship to Diabetes Self-Care Activities in African Americans. Diabetes Educator. 2013;39(2):231-9. |  |  |  |  | x |  |
| Weigensberg MJ, Lane CJ, Avila Q, Konersman K, Ventura E, Adam T, et al. Imagine HEALTH: results from a randomized pilot lifestyle intervention for obese Latino adolescents using Interactive Guided ImagerySM. BMC complementary and alternative medicine. 2014;14:28. |  |  | x |  |  |  |
| Weiler DM, Crist JD. Diabetes self-management in a Latino social environment. The Diabetes educator. 2009;35(2):285-92. |  |  |  |  | x |  |
| Weitzman PF, Caballero AE, Millan-Ferro A, Becker AE, Levkoff SE. Bodily aesthetic ideals among Latinas with type 2 diabetes: implications for treatment adherence, access, and outcomes. The Diabetes educator. 2013;39(6):856-63. |  |  |  |  | x |  |
| Wenger A, Mischke C. ["A Little Bit of Switzerland, a Little Bit of Kosovo". Swiss Immigrants from Former Yugoslavia with Type 2 Diabetes. A Qualitative Study' in Analogy to Grounded Theory]. Gesundheitswesen (Bundesverband der Arzte des Offentlichen Gesundheitsdienstes (Germany)). 2015;77(10):736-41. |  |  |  | x |  |  |
| Wofford L, Froeber D, Clinton B, Ruchman E. Free afterschool program for at-risk African American children: findings and lessons. Family & community health. 2013;36(4):299-310. | x |  |  |  |  |  |
| Yates T, Davies M, Khunti K. Preventing type 2 diabetes: can we make the evidence work? Postgraduate medical journal. 2009;85(1007):475-80. |  |  | x |  |  |  |
| Yeboah-Korang A, Kleppinger A, Fortinsky RH. Racial and Ethnic Group Variations in Service Use in a National Sample of Medicare Home Health Care Patients with Type 2 Diabetes Mellitus. Journal of the American Geriatrics Society. 2011;59(6):1123-9. |  |  |  |  | x |  |
| Yee LM, McGuire JM, Taylor SM, Niznik CM, Simon MA. Social and environmental barriers to nutrition therapy for diabetes management among underserved pregnant women: A qualitative analysis. Journal of nutrition education and behavior. 2016;48(3):170-80. |  |  |  |  | x |  |
| Yee LM, McGuire JM, Taylor SM, Niznik CM, Simon MA. Factors promoting diabetes self-care among low-income, minority pregnant women. Journal of Perinatology. 2016;36(1):13-8. |  |  |  |  | x |  |
| Yee LM, Niznik CM, Simon MA. Examining the Role of Health Literacy in Optimizing the Care of Pregnant Women with Diabetes. American journal of perinatology. 2016;33(13):1242-9. |  |  | x |  |  |  |
| Yeh MC, Heo M, Suchday S, Wong A, Poon E, Liu G, et al. Translation of the Diabetes Prevention Program for diabetes risk reduction in Chinese immigrants in New York City. Diabetic medicine: a journal of the British Diabetic Association. 2016;33(4):547-51. |  |  |  |  | x |  |
| Yeoh S, Furler J. Perceptions of Health and Diabetes in a Melbourne South Sudanese Community. Journal of Immigrant & Minority Health. 2011;13(5):914-8. |  |  |  |  | x |  |
| Yilmaz-Aslan Y, Brzoska P, Bluhm M, Aslan A, Razum O. Illness perceptions in Turkish migrants with diabetes: a qualitative study. Chronic illness. 2014;10(2):107-21. |  |  |  |  | x |  |
| Yoo HJ, Park MS, Kim TN, Yang SJ, Cho GJ, Hwang TG, et al. A Ubiquitous Chronic Disease Care system using cellular phones and the internet. Diabetic medicine: a journal of the British Diabetic Association. 2009;26(6):628-35. |  |  |  |  |  | x |
| Young SA, Azam LS, Meurer JR, Hill RS, Chen C. The Influence of Patient and Provider Communication on Diabetes Care Delivery. Journal of Ambulatory Care Management. 2016;39(3):272-8. |  |  | x |  |  |  |
| Yu CH, Parsons JA, Hall S, Newton D, Jovicic A, Lottridge D, et al. User-centered design of a web-based self-management site for individuals with type 2 diabetes - providing a sense of control and community. BMC medical informatics and decision making. 2014;14:60. |  |  | x |  |  |  |
| Zeh P, Sandhu HK, Cannaby AM, Sturt JA. The impact of culturally competent diabetes care interventions for improving diabetes-related outcomes in ethnic minority groups: a systematic review. Diabetic medicine : a journal of the British Diabetic Association. 2012;29(10):1237-52. |  |  |  | x |  |  |
| Zeh P, Sandhu HK, Cannaby AM, Warwick J, Sturt JA. Exploring culturally competent primary care diabetes services: a single-city survey. Diabetic medicine : a journal of the British Diabetic Association. 2016;33(6):786-93. |  | x |  |  |  |  |
| Zeng B, Sun WJ, Gary RA, Li CW, Liu TT. Towards a Conceptual Model of Diabetes Self-Management among Chinese Immigrants in the United States. International journal of environmental research and public health. 2014;11(7):6727-42. |  |  |  |  | x |  |
| Zhang X, Beckles GL, Bullard KM, Gregg EW, Albright AL, Barker L, et al. Access to health care and undiagnosed diabetes along the United States-Mexico border. Revista panamericana de salud publica = Pan American journal of public health. 2010;28(3):182-9. |  |  |  | x |  |  |
| Zhang X, Geiss LS, Cheng YJ, Beckles GL, Gregg EW, Kahn HS. The missed patient with diabetes: how access to health care affects the detection of diabetes. Diabetes care. 2008;31(9):1748-53. |  |  |  | x |  |  |
| Zolfaghari M, Mousavifar SA, Haghani H. Mobile phone text messaging and Telephone follow-up in type 2 diabetic patients for 3 months: A comparative study. Journal of diabetes and metabolic disorders. 2012;11(1). |  |  |  |  |  | x |
| Zulfiqar T, Lithander FE, Banwell C, Young R, Boisseau L, Ingle M, et al. Barriers to a healthy lifestyle post gestational-diabetes: An Australian qualitative study. Women and birth: journal of the Australian College of Midwives. 2017;30(4):319-24. |  |  | x |  |  |  |
| Zulfiqar T, Nolan CJ, Banwell C, Young R, Boisseau L, Ingle M, et al. Barriers to a healthy lifestyle for three- to four-year-old children of Australian-born and overseas-born mothers with post-gestational diabetes: An Australian qualitative study. Journal of child health care: for professionals working with children in the hospital and community. 2018:1367493518759240. |  |  | x |  |  |  |
| **Full text screening of grey literature** | **nft** | **P1** | **P2** | **Concept 1** | **Concept 2** | **Context** |
| Fischer HH, Fischer IP, Pereira RI, Furniss AL, Rozwadowski JM, Moore SL, et al. Text Message Support for Weight Loss in Patients With Prediabetes: A Randomized Clinical Trial. Diabetes care. 2016;39(8):1364-70. |  |  |  |  | x |  |
| Organization WH. Global report on diabetes: World Health Organization; 2016. |  |  | x |  |  |  |
| Seligman HK, Schillinger D. Food Insecurity and Diabetes Prevention and Control in California: UCSF Center for Vulnerable Populations; 2010. |  |  | x |  |  |  |
| **Full text screening of studies identified by reference check** | **nft** | **P1** | **P2** | **Concept 1** | **Concept 2** | **Context** |
| "Devia C, Ruddock C, Golub M, et al. Addressing obesity in underserved communities through a faith-based nutrition and fitness program. In: Brennan V, Kumanyika S, Zambrana R, eds. Obesity Interventions in Underserved U.S. Communities. Baltimore, MD: Johns Hopkins University Press. In press, 2014." | x |  |  |  |  |  |
| Dean, D. A., Griffith, D. M., McKissic, S. A., Cornish, E. K., & Johnson-Lawrence, V. (2016). Men on the Move– Nashville: Feasibility and acceptability of a technologyenhanced physical activity pilot intervention for overweight and obese middle and older age African American men. American Journal of Men’s Health. doi:1557988316644174 |  | x |  |  |  |  |
| Jaber LA, et al. Feasibility of group lifestyle intervention for diabetes prevention in Arab Americans. Diabetes Res Clin Pract. 2011;91(3):307–15. |  |  |  |  | x |  |
| Kanaya AM, Santoyo-Olsson J, Gregorich S, Grossman M, Moore T, Stewart AL. The Live Well, Be Well Study: a community-based, translational lifestyle program to lower diabetes risk factors in ethnic minority and lower-socioeconomic status adults. Am J Public Health. 2012;102:1551-1558. |  |  |  |  | x |  |
| Moredich CA, Kessler TA. Physical activity and nutritional weight loss interventions in obese, low-income women: an integrative review. Journal of midwifery & women's health. 2014;59(4):380–7. |  | x |  |  |  |  |
| Patel, M., Phillips-Caesar, E. and Boutin-Foster, C. (2011) Barriers to lifestyle behavioral change in migrant South Asian populations. Journal of Immigrant and Minority Health, 14, 774–785. |  |  |  |  | x |  |
| Pinelli, N. R., Brown, M. B., Herman, W. H. and Jaber, L. A. (2011) Family support is associated with success in achieving weight loss in a group lifestyle intervention for diabetes prevention in Arab Americans. Ethnicity & Disease, 21, 480–484. |  |  |  |  | x |  |
| Renzaho, A. M., McCabe, M. and Swinburn, B. (2012) Intergenerational differences in food, physical activity, and body size perceptions among African migrants. Qualitative Health Research, 22, 740–754. |  |  |  |  | x |  |
| Renzaho, A. M., Mellor, D., Boulton, K. and Swinburn, B. (2010) Effectiveness of prevention programmes for obesity and chronic diseases among immigrants to developed countries—a systematic review. Public Health Nutrition, 13, 438–450. |  |  |  |  | x |  |
| Walker LO, Sterling BS, Latimer L, Kim SH, Garcia AA, Fowles ER. Ethnicspecific weight-loss interventions for low-income postpartum women: findings and lessons. West J Nurs Res. 2012;34(5):654–76. |  | x |  |  |  |  |
| **Update full text screening** | **nft** | **P1** | **P2** | **Concept 1** | **Concept 2** | **Context** |
| Abuelmagd W, Hakonsen H, Mahmood KQU, Taghizadeh N, Toverud EL. Living with Diabetes: Personal Interviews with Pakistani Women in Norway. Journal of Immigrant and Minority Health. 2018;20(4):848-53. |  |  |  | x |  |  |
| Aguayo-Mazzucato C, Diaque P, Hernandez S, Rosas S, Kostic A, Caballero AE. Understanding the growing epidemic of type 2 diabetes in the Hispanic population living in the United States. Diabetes Metab Res Rev. 2019;35(2):e3097. |  |  |  |  | x |  |
| Almansour HA, Chaar B, Saini B. Perspectives and experiences of patients with type 2 diabetes observing the Ramadan fast. Ethnicity & Health. 2018;23(4):380-96. |  |  |  | x |  |  |
| Amirehsani KA, Hu J, Wallace DC, Silva ZA, Dick S. Hispanic Families' Action Plans for a Healthier Lifestyle for Diabetes Management. Diabetes Educator. 2019;45(1):87-95. |  |  |  | x |  |  |
| Apostolopoulos Y, Lemke MK, Hosseinichimeh N, Harvey IS, Lich KH, Brown J. Embracing Causal Complexity in Health Disparities: Metabolic Syndemics and Structural Prevention in Rural Minority Communities. Prev Sci. 2018;19(8):1019-29. |  |  |  |  | x |  |
| Aweko J, De Man J, Absetz P, Ostenson CG, Peterson SS, Alvesson HM, et al. Patient and Provider Dilemmas of Type 2 Diabetes Self-Management: A Qualitative Study in Socioeconomically Disadvantaged Communities in Stockholm. International Journal of Environmental Research and Public Health. 2018;15(9). |  |  |  |  | x |  |
| Azam L, Meurer J, Nelson D, Asan O, Flynn K, Knudson P, et al. Cultural and Social Challenges of Diabetes Self-Management Education Through Physicians' Voices. Wmj. 2018;117(5):219-23. |  | x |  |  |  |  |
| Bech LK, Jacobsen CB, Mathiesen AS, Thomsen T. Preferring to manage by myself: A qualitative study of the perspectives of hardly reached people with type 2 diabetes on social support for diabetes management. Journal of Clinical Nursing. 2019;28(9-10):1889-98. |  |  | x |  |  |  |
| Chang C, Khurana S, Strodel R, Camp A, Magenheimer E, Hawley N. Perceived Barriers to Physical Activity Among Low-Income Latina Women at Risk for Type 2 Diabetes. Diabetes Educator. 2018;44(5):444-53. |  |  |  |  | x |  |
| Chesla CA, Chun KM, Kwan CML. Biculturalism in couple support for diabetes care in US-born Chinese Americans. Research in Nursing & Health. 2019;42(1):39-47. |  |  |  | x |  |  |
| Dayyani I, Terkildsen Maindal H, Rowlands G, Lou S. A qualitative study about the experiences of ethnic minority pregnant women with gestational diabetes. Scand J Caring Sci. 2019. |  |  |  |  | x |  |
| Felix H, Rowland B, Long CR, Narcisse M-R, Piel M, Goulden PA, et al. Diabetes self-care behaviors among Marshallese adults living in the United States. Journal of Immigrant and Minority Health. 2018;20(6):1500-7. |  |  |  |  | x |  |
| Felix HC, Narcisse MR, Long CR, English E, Haggard-Duff L, Purvis RS, et al. The Effect of Family Diabetes Self-management Education on Self-care Behaviors of Marshallese Adults with Type 2 Diabetes. American Journal of Health Behavior. 2019;43(3):490-7. |  |  |  |  | x |  |
| Goff LM, Moore AP, Rivas C, Harding S. Healthy Eating and Active Lifestyles for Diabetes (HEAL-D): study protocol for the design and feasibility trial, with process evaluation, of a culturally tailored diabetes self-management programme for African-Caribbean communities. Bmj Open. 2019;9(2). |  |  |  |  | x |  |
| Gubrium A, Leckenby D, Harvey MW, Marcus BH, Rosal MC, Chasan-Taber L. Perspectives of health educators and interviewers in a randomized controlled trial of a postpartum diabetes prevention program for Latinas: a qualitative assessment. BMC Health Serv Res. 2019;19(1):357. |  | x |  |  |  |  |
| Herrick CJ, Keller MR, Trolard AM, Cooper BP, Olsen MA, Colditz GA. Postpartum diabetes screening among low income women with gestational diabetes in Missouri 2010-2015. BMC Public Health. 2019;19(1):148. |  |  |  |  | x |  |
| Hjelm K, Bard K, Apelqvist J. A qualitative study of developing beliefs about health, illness and healthcare in migrant African women with gestational diabetes living in Sweden. Bmc Womens Health. 2018;18. |  |  |  |  | x |  |
| Islam NS, Wyatt LC, Taher MD, Riley L, Tandon SD, Tanner M, et al. A culturally tailored community health worker intervention leads to improvement in patient-centered outcomes for immigrant patients with type 2 diabetes. Clinical Diabetes. 2018;36(2):100-11. |  |  |  | x |  |  |
| Jacobson LT, Zackula R, Redmond ML, Duong J, Collins TC. Pioneer baby: suggestions for pre- and postnatal health promotion programs from rural English and Spanish-speaking pregnant and postpartum women. Journal of Behavioral Medicine. 2018;41(5):653-67. |  |  | x |  |  |  |
| Kim MY, Lee EJ. Factors Affecting Self-Care Behavior Levels among Elderly Patients with Type 2 Diabetes: A Quantile Regression Approach. Medicina (Kaunas). 2019;55(7). |  |  |  |  |  | x |
| Maine A, Brown MJ, Dickson A, Truesdale M. An evaluation of mainstream type 2 diabetes educational programmes in relation to the needs of people with intellectual disabilities: A systematic review of the literature. J Appl Res Intellect Disabil. 2019;32(2):256-79. |  |  |  |  | x |  |
| Maine A, Brown MJ, Dickson A, Truesdale M. Pilot feasibility study of the Walking Away from Diabetes programme for adults with intellectual disabilities in two further education colleges: Process evaluation findings. Journal of Applied Research in Intellectual Disabilities. 2019;32(5):1034-46. |  |  |  |  | x |  |
| Maneze D, Ramjan L, DiGiacomo M, Everett B, Davidson PM, Salamonson Y. Negotiating health and chronic illness in Filipino-Australians: a qualitative study with implications for health promotion. Ethnicity & Health. 2018;23(6):611-28. |  | x |  |  |  |  |
| Manios Y, Androutsos O, Lambrinou CP, Cardon G, Lindstrom J, Annemans L, et al. A school- and community-based intervention to promote healthy lifestyle and prevent type 2 diabetes in vulnerable families across Europe: design and implementation of the Feel4Diabetes-study. Public Health Nutr. 2018;21(17):3281-90. |  |  |  |  |  | x |
| McEwen MM, Pasvogel A, Elizondo-Pereo R, Meester I, Vargas-Villarreal J, Gonzalez-Salazar F. Diabetes Self-Management Behaviors, Health Care Access, and Health Perception in Mexico-US Border States. Diabetes Educ. 2019;45(2):164-73. |  |  |  |  |  | x |
| Ndwiga DW, McBride KA, Simmons D, MacMillan F. Diabetes, its risk factors and readiness to change lifestyle behaviours among Australian Samoans living in Sydney: Baseline data for church-wide interventions. Health Promotion Journal of Australia. |  |  |  | x |  |  |
| Okoro FO, Veri S, Davis V. Culturally Appropriate Peer-Led Behavior Support Program for African Americans With Type 2 Diabetes. Front Public Health. 2018;6:340. |  |  |  |  | x |  |
| Pan W, Ge SQ, Xu Y, Toobert D. Cross-Validating a Structural Model of Factors Influencing Diabetes Self-Management in Chinese Americans with Type 2 Diabetes. Journal of Transcultural Nursing. 2019;30(2):163-72. |  |  |  |  | x |  |
| Saunders T. Type 2 Diabetes Self-Management Barriers in Older Adults An Integrative Review of the Qualitative Literature. Journal of Gerontological Nursing. 2019;45(3):43-54. |  |  |  |  | x |  |
| Scarton L, Velazquez I, O'Neal LJ, Iyerm S, Cannady T, Choate A, et al. Developing a culturally tailored multigenerational intervention to prevent and manage type 2 diabetes in American Indian families living in rural settings: Findings from a focus group study. Research in Nursing & Health. 2019;42(3):226-33. |  | x |  |  |  |  |
| Sherman LD, Fawole T. "The More I Do, the Better I'll Be": The Treatment Preferences of Type 2 Diabetes Among African American Men. American Journal of Mens Health. 2018;12(4):779-87. |  |  |  | x |  |  |
| Sherman LD, Williams JS. Perspectives of Fear as a Barrier to Self-Management in Non-Hispanic Black Men With Type 2 Diabetes. Health Education & Behavior. 2018;45(6):987-96. |  |  |  | x |  |  |
| Shiyanbola OO, Ward E, Brown C. Sociocultural Influences on African Americans' Representations of Type 2 Diabetes: A Qualitative Study. Ethnicity & Disease. 2018;28(1):25-32. |  |  |  | x |  |  |
| Shiyanbola OO, Ward EC, Brown CM. Utilizing the common sense model to explore African Americans' perception of type 2 diabetes: A qualitative study. PLoS One. 2018;13(11):e0207692. |  |  |  | x |  |  |
| Siad FM, Fang XY, Santana MJ, Butalia S, Hebert MA, Rabi DM. Understanding the Experiences of East African Immigrant Women With Gestational Diabetes Mellitus. Can J Diabetes. 2018;42(6):632-8. |  |  |  | x |  |  |
| Sung KW, Kang HS, Nam JR, Park MK, Park JH. [The Effects of a Health Mentoring Program in Community-dwelling Vulnerable Elderly Individuals with Diabetes]. J Korean Acad Nurs. 2018;48(2):182-94. |  |  |  |  |  | x |
| Tan CCL, Cheng KKF, Sum CF, Shew JSH, Holydard E, Wang WR. Perceptions of Diabetes Self-Care Management Among Older Singaporeans With Type 2 Diabetes: A Qualitative Study. Journal of Nursing Research. 2018;26(4):242-9. |  |  | x |  |  |  |
| Terragni L, Beune E, Stronks K, Davidson E, Qureshi S, Kumar B, et al. Developing culturally adapted lifestyle interventions for South Asian migrant populations: a qualitative study of the key success factors and main challenges. Public Health. 2018;161:50-8. |  |  |  | x |  |  |
| Van Stappen V, Latomme J, Cardon G, De Bourdeaudhuij I, Lateva M, Chakarova N, et al. Barriers from Multiple Perspectives Towards Physical Activity, Sedentary Behaviour, Physical Activity and Dietary Habits When Living in Low Socio-Economic Areas in Europe. The Feel4Diabetes Study. Int J Environ Res Public Health. 2018;15(12). |  | x |  |  |  |  |
| Wah YYE, McGill M, Wong J, Ross GP, Harding AJ, Krass I. Self-management of gestational diabetes among Chinese migrants: A qualitative study. Women and Birth. 2019;32(1):E17-E23. |  |  |  | x |  |  |
| Waller K, Furber S, Bauman A, Allman-Farinelli M, van den Dolder P, Hayes A, et al. DTEXT - text messaging intervention to improve outcomes of people with type 2 diabetes: protocol for randomised controlled trial and cost-effectiveness analysis. Bmc Public Health. 2019;19. |  |  | x |  |  |  |
| Zhang ZS, Monro J, Venn BJ. Development and Evaluation of an Internet-Based Diabetes Nutrition Education Resource. Nutrients. 2019;11(6). |  |  | x |  |  |  |

Nft: no full text available; P1: Diabetes mellitus type 2 or gestation diabetes; P2: Vulnerable groups; Concept 1: Prevention; Concept 2: Barriers and facilitating factors; Context: >2008; WHO stratum A
