# Supplementary material for: Communication strategies in the prevention of type 2 diabetes and gestational diabetes in vulnerable groups: a scoping review
Source: Syst Rev. 2021 Nov 24;10:301. doi: 10.1186/s13643-021-01846-8 (PMC8611985; doi:10.1186/s13643-021-01846-8)
Supplement: Supplementary file 4 — Additional file 4 : Supplement 4. List of WHO stratum A countries. [file 13643_2021_1846_MOESM4_ESM.docx]

Supplement 4 Table 8: List of WHO stratum a countries

| Regions | States |
| --- | --- |
| Americas | Canada, Cuba, United States of America |
| Europe | Andorra, Austria, Belgium, Croatia, Cyprus, Czech Republic, Denmark, Finland, France, Germany, Greece, Iceland, Ireland, Israel, Italy, Luxembourg, Malta, Monaco, Netherlands, Norway, Portugal, San Marino, Slovenia, Spain, Sweden, Switzerland, United Kingdom |
| Western Pacific | Australia, Brunei Darussalam, Japan, New Zealand, Singapore |
